# The Influence of 150-Cavity Binders on the Dynamics of Influenza A Neuraminidases as Revealed by Molecular Dynamics Simulations and Combined Clustering

**DOI:** 10.1371/journal.pone.0059873

**Published:** 2013-03-27

**Authors:** Kyle T. Greenway, Eric B. LeGresley, B. Mario Pinto

**Affiliations:** Department of Chemistry, Simon Fraser University, Burnaby, British Columbia, Canada; Oak Ridge National Laboratory, United States of America

## Abstract

Neuraminidase inhibitors are the main pharmaceutical agents employed for treatments of influenza infections. The neuraminidase structures typically exhibit a 150-cavity, an exposed pocket that is adjacent to the catalytic site. This site offers promising additional contact points for improving potency of existing pharmaceuticals, as well as generating entirely new candidate inhibitors. Several inhibitors based on known compounds and designed to interact with 150-cavity residues have been reported. However, the dynamics of any of these inhibitors remains unstudied and their viability remains unknown. This work reports the outcome of long-term, all-atom molecular dynamics simulations of four such inhibitors, along with three standard inhibitors for comparison. Each is studied in complex with four representative neuraminidase structures, which are also simulated in the absence of ligands for comparison, resulting in a total simulation time of 9.6µs. Our results demonstrate that standard inhibitors characteristically reduce the mobility of these dynamic proteins, while the 150-binders do not, instead giving rise to many unique conformations. We further describe an improved RMSD-based clustering technique that isolates these conformations – the structures of which are provided to facilitate future molecular docking studies – and reveals their interdependence. We find that this approach confers many advantages over previously described techniques, and the implications for rational drug design are discussed.

## Introduction

Influenza A and B viruses are responsible for the respiratory disease commonly referred to as ‘the flu’, with infections ranging from epidemics to pandemics and symptoms ranging from mild to life-threatening. Inhibitors of viral neuraminidase (NA) have been the mainstays of pharmaceutical treatment of influenza since their initial introduction in 1999 [1]. Influenza NA is a tetrameric exoglycohydrolase affixed to the viral membrane, which facilitates viral proliferation by cleaving terminal sialic acid linkages on the host cell to effect release of viral progeny. There are nine known serotypes of NA (N1–N9) found in influenza A. These types are further categorized into two groups based on phylogenetic analysis; group-1: N1, N4, N5, N8, and group-2: N2, N3, N6, N7, N9 [2]. All known NA possess highly conserved active site residues and conformations, although crystallography has demonstrated that group-1 NA generally exhibit a cavity adjacent to the main sialic-acid-binding site that is not apparent in group-2 NA crystal structures (CS). This cavity is known as the 150-cavity as its accessibility is limited by a mobile loop composed of residues 147–152, similarly known as the 150-loop. The 150-loop has been crystallized in ‘open’ and ‘closed’ conformations [2], [3], which provide snapshots along a conformational itinerary.

The successful design of the two FDA-approved NA inhibitors, zanamivir (Relenza) and oseltamivir (Tamiflu), can be attributed in part to the conserved active site residues, the relative stability of group-2 NA, and the lack of significant changes to the active site upon ligand binding [1]. This is evidenced by the fact that the rational structure-based design of these inhibitors was based exclusively on the group-2 NA active site [1], predating discovery of the 150-cavity in group-1 NA by some years [2]. Despite their success, these inhibitors have limitations. Specifically, zanamivir suffers from high polarity [1], oseltamivir is highly vulnerable to inactivation due to viral mutation [4], and both exhibit mixed clinical efficacy [5]. One recent strategy for simultaneously improving the potency, lipophilicity, and capacity to resist mutations of these compounds has been to attach groups, usually hydrophobic, to a similar ring framework in order to form additional points of contact within the 150-cavity [2]. Several such compounds, known as 150-binders in this work, have been synthesized [6]–[9] and many more have been proposed (see [10] for a recent review). Some of these inhibitors have been shown by X-ray crystallography to successfully enter the 150-cavity and affect the orientation of the 150-loop [6], and others have demonstrated powerful inhibitory activity in cellular assays [8], [9] and, recently, *in vivo* [8]. In contrast to zanamivir and oseltamivir, 150-binders are designed to interact primarily with the loop-open NA conformations. This binding mode is promising in that the group-1 loop-open NA conformation is thought to be lower in energy than the loop-closed conformation [11], at least in group-1 NA, but it necessitates targeting portions of neuraminidase that are far more dynamic than the active site, requiring a more sophisticated understanding of NA-ligand dynamics. Crystallography is likely to be less informative in this process as multiple pieces of evidence have demonstrated the ambiguity of the static structures of these dynamic systems. Specifically, adjusting crystallization conditions can result in different structures for identical systems [2], and it has been shown recently that even strong binders can adopt distinct conformations in different CS [12]. Further, MD simulations have revealed features previously unobserved by crystallography, such as populated conformations in which the 150-loop is open more widely than seen in any CS [13], and that the 150-loop is predominantly open in 2009 pandemic N1 and occasionally open in N2 simulations [11], despite their CS exhibiting only loop-closed conformations [2]. Moreover, as we demonstrate in this work, oseltamivir and zanamivir generally reduce the mobility of various viral neuraminidases, while 150-binders typically exert the opposite effect, giving rise to conformations that are not seen in simulations or CS of neuraminidase in complex with standard binders. These factors necessitate a more complex approach that examines the interdependency of enzyme and ligand dynamics.

**Figure 1:**
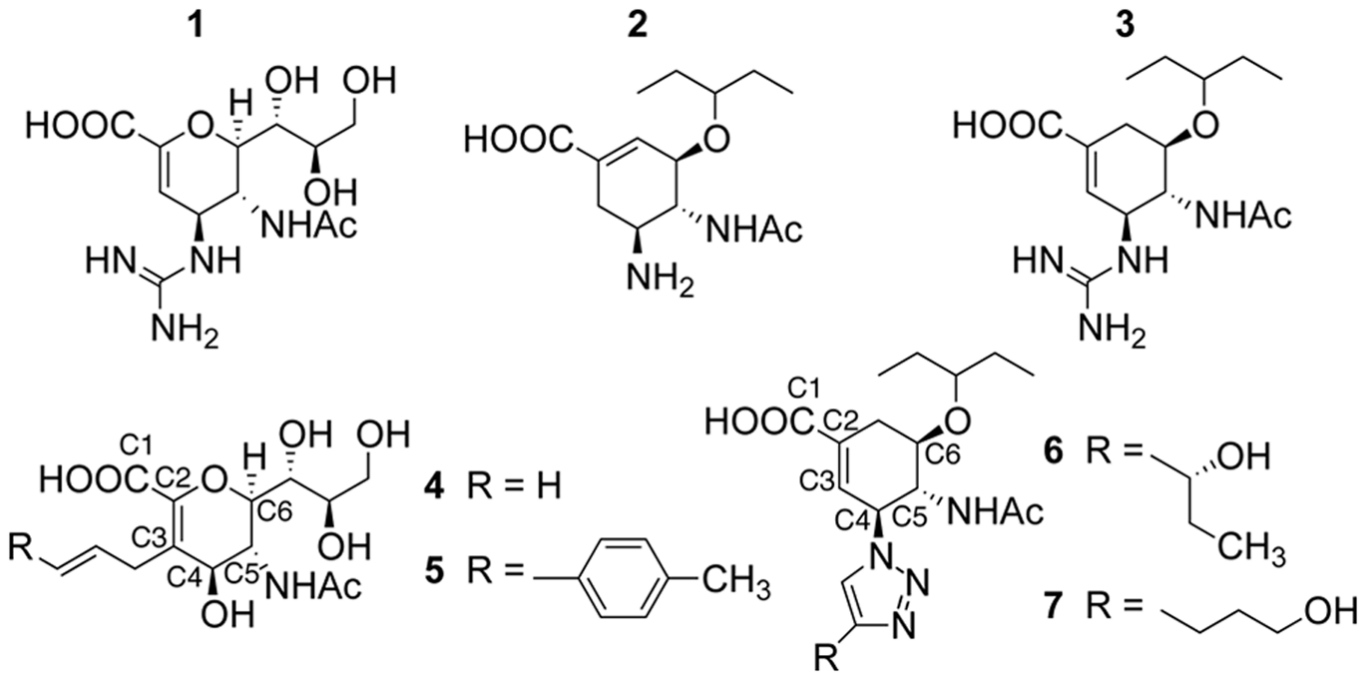
Compounds of interest.

**Figure 2:**
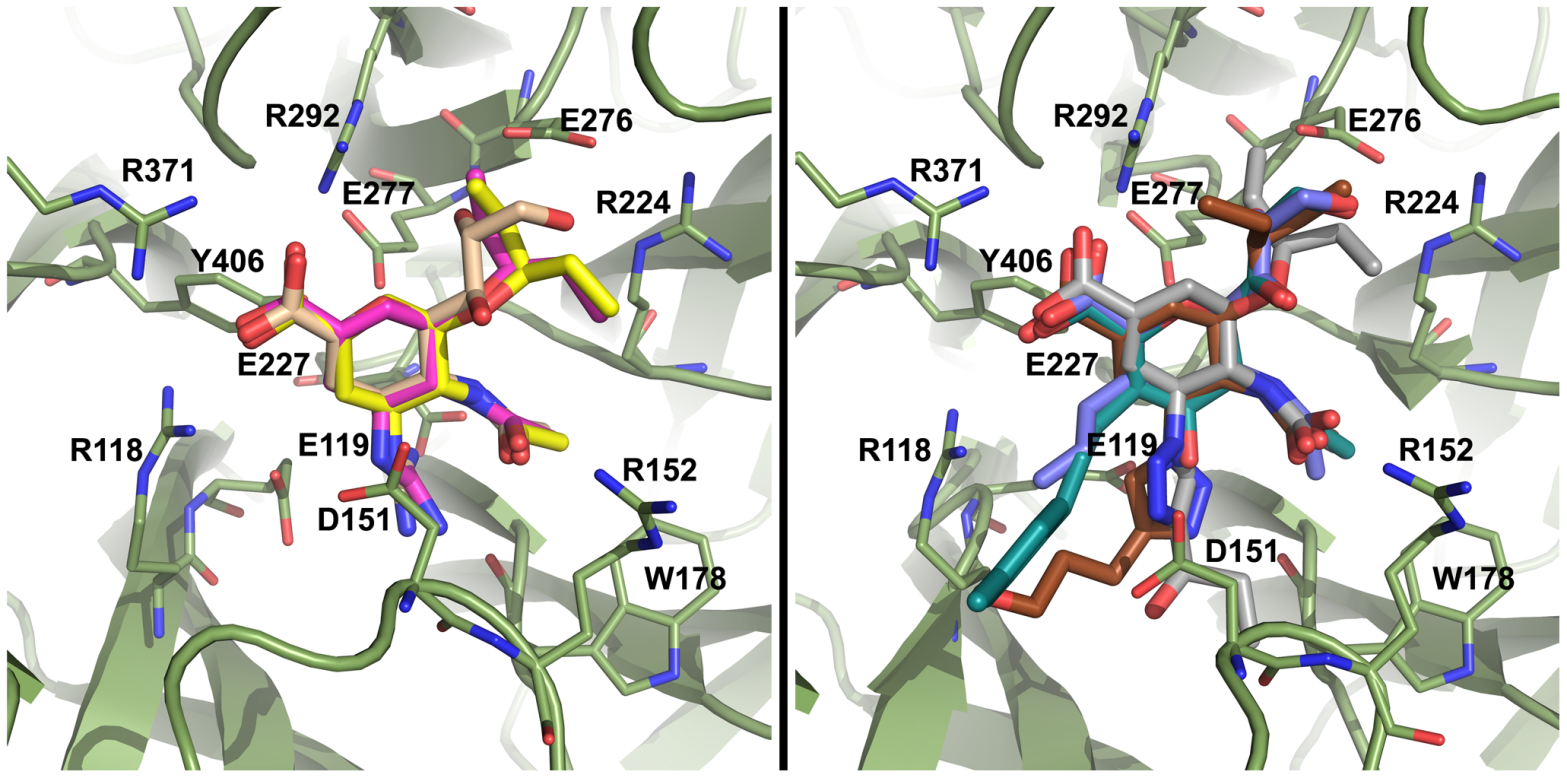
Neuraminidase active site, N8_closed_ and N8_open_. Active site of N8_closed_ (left) with standard binders **1–3** and N8_open_ (right) with 150-binders **4–7**. Compound **1** is shown in beige, **2** in yellow, **3** in purple, **4** in light blue, **5** in teal, **6** in grey, and **7** in brown. Key residues are labeled.

In this work, we seek to study such dynamics, utilizing long-term MD simulations of a variety of NA, uncomplexed (apo) and in complex with seven different inhibitors (holo), shown in Figure 1. The ligands selected include the two commercial inhibitors, zanamivir **1** and oseltamivir carboxylate **2**, as well as a similar inhibitor currently undergoing further study: the double-bond isomer and guanidine derivative of oseltamivir **3** [7]. These compounds, which we collectively refer to as “standard inhibitors”, serve both as controls and points of comparison to previous computational and experimental studies. Additionally, four 150-binders have been selected: two (**4** and **5**) featuring an alkene-linked sidechain attached at C3 [6], and two (**6** and **7**) featuring a triazole-linked sidechain attached at C4 [7]. Figure 2 shows the CS conformations of these ligands, with **1**, **2**, **4**, and **5** from the PDB structures 2HTQ, 2HT8, 309J, and 309 K respectively, and **3**, **6**, and **7** from unpublished data. In all cases, these binding modes of the ligands are similar. Inhibitors **4–7** have been studied experimentally, and other proposed 150-binders have been studied by molecular docking [14]–[16] and brief MD simulations [17], [18]. However, there have been no reports on the complex ligand-enzyme dynamics exhibited during long-term MD simulations of 150-binders.

Four neuraminidase CS that represent the diversity of known NA structures were chosen for this work. These are an N2 structure (PDB code 2AEP and referred to as N2), the 2009 pandemic H1N1 structure (PDB code 3NSS and referred to as N1_09_), and the loop-closed and loop-open CS of N8 as crystallized with **2** (PDB codes 2HT7 and 2HT8, and referred to as N8_closed_ and N8_open_, respectively.) N2 was selected as representative of group-2 NA, which is known to be relatively static and exhibit a high tendency towards loop-closed conformations [19]. N1_09_ was chosen as an atypical group-1 NA that is less dynamic and more prone to a loop-closed orientation than is expected for this group [3]. N8 was selected as a typical group-1 neuraminidase with CS available for both the closed and open conformations. These structures are useful as the N8_closed_ structure highly resembles both N2 and N1_09_ in conformation, facilitating comparison between their dynamics, while comparisons between N8_open_ and N8_closed_ offer insights into the effects of loop-closed versus loop-open conformations and the importance of starting-structures in MD simulations [2].

Simulations of all four NA structures were conducted with all seven ligands, as well as without any ligand, for three separately equilibrated runs of 100 ns in length, resulting in a total simulation time of 9.6 µs. The questions we set out to investigate in this study are numerous. We sought to explore 1) the most populated conformations of each ligand and each neuraminidase, as well as their interdependency, 2) the active site residues’ stability or flexibility in response to the various 150-binders, and the implications for drug design, and 3) the commonalties among and between the various ligands, specifically in regards to **3**, which shares features with each **1** and **2**, i.e. the pentyloxy chain of **2** and the guanidine moiety of **1**.

We further demonstrate an improvement on the RMSD-based clustering techniques that have proven valuable in numerous MD studies, including multiple studies of influenza NA [11], [16]. Standard clustering techniques were unsuitable for this study due to the extensive simulation time, the flexibility of NA and ligands observed, and the variety of NA and ligands included. Standard clustering of each ligand-enzyme combination would result in 28 separate groups of clusters, with each structure in each group specific to that particular NA-ligand combination and therefore not necessarily comparable to any other cluster structure. Instead, we have extracted and concatenated the enzyme coordinates from each run prior to clustering (see Materials and Methods) in a process we refer to as “combined clustering”. The result is just four separate groups of clusters that are common within all simulations of a given enzyme, thereby facilitating comparisons. The ligands are then separately clustered, separating the dynamics of the ligands from those of the enzyme.

Owing to our primary interest in the important interactions, this clustering was performed on the key residues of the active site as shown in Figure 2, which are conserved across all NA employed in this study. We excluded the 150-loop residues 147–150 as these have been shown to be energetically unimportant for standard inhibitors and fluctuate significantly [4]. We have additionally employed a technique described in previous studies of measuring the cavity-width in each simulation [4], [11]. The width measurements were correlated with the distances between ligand and enzyme’s center of mass (COM) and their utility is compared to RMSD-based clustering.

## Results

### Cavity-width and Loop Dynamics

Measurements of 150-cavity-widths, given in Table 1, generally reflect conclusions from previous reports [4], [11]. Specifically, N2 is consistently in a closed-cavity state and shows little influence from ligands. N1_09_ is more dynamic, although it exhibits a closed-cavity for 70% of all simulations and only slightly more in the apo simulation. As expected, this is less flexible than N8; the comparable N8_closed_ remains closed for only 43% of the simulations. The same N8 systems starting from an open position consistently adopt more open conformations, spending an average of only 8% of the simulation with a closed 150-cavity. This indicates that the 20 ns of simulation time that was removed from the start of each production run is insufficient to completely overcome the bias of the loop starting position. The question of starting-conformation bias is discussed in detail below.

**Table 1:** Loop-closed populations based on cavity-width.

	Loop-Closed Population			
Ligand	N2	N1_09_	N8_closed_	N8_open_
1	93%	75%	53%	29%
2	100%	97%	21%	1%
3	71%	76%	38%	12%
4	77%	35%	17%	12%
5	100%	87%	36%	1%
6	100%	17%	67%	2%
7	100%	100%	70%	0%
Apo	100%	74%	42%	4%
Average	93%	70%	43%	8%
Std. Dev.	12%	29%	20%	10%

**Figure 3:**
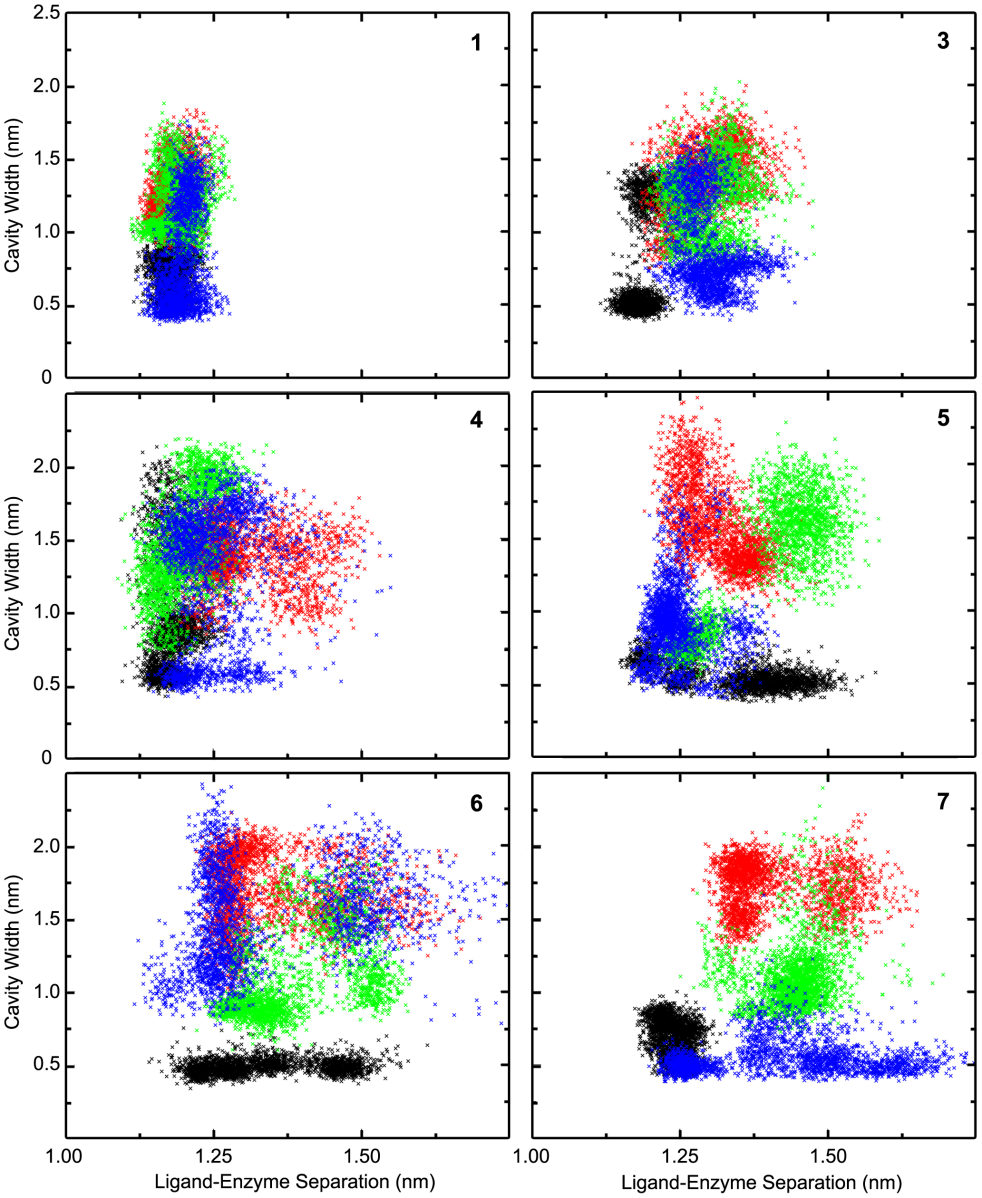
Comparison of cavity-width and ligand distance from active site. Compound numbers are indicated within the frame, and neuraminidases are as follows: N2 shown in black, N1_09_ in blue, N8_closed_ in green, and N8_open_ in red. Compound **2** shows a profile that is extremely similar to **1** and can be found in Supporting Information.

Figure 3 displays the relationship between the cavity-width and the ligand’s position by comparing the distance between the ligand and the enzyme’s respective center of masses to the cavity-width for that frame. The plot of compound **1** illustrates the potency and stability of the standard binders, which are extremely static within the enzyme’s active site and do not significantly alter the 150-loop’s position relative to the apo enzyme runs. Compound **2** is similar (Figure S1), while the 150-binders demonstrate a variety of conformations within and outside the active-site, as reflected in their COM distances.

Notably, the cavity-open conformation that is not seen in the apo-N2 simulations is observed when N2 is complexed with **1**, as well with ligands **3** and **4**. In all three cases, this takes place in one of the three triplicate runs where the D147–H150 interaction is lost, as previously reported [11], and only regained in the case of **1**. The inconsistent appearance of an open 150-cavity for N2, never occurring in more than one of the triplicate runs for a given complex, is likely due to a lack of sampling. There is no clear reason why ligands **5–7** would not similarly induce the 150-cavity to open, or why apo-N2 is not observed with an open conformation, as has been previously reported [11].

For the group-1 NA simulations, the results apparently contradict the hypothesis that ligands facilitate loop-closure and 150-binders inhibit loop-closure [6]. For example, ligands **6** and **7** in N8_closed_ simulations are better able to encourage loop-open conformations than even the standard binders. The simulations of the remaining N8_closed_ complexes are similar to the enzyme-only state. N8_open_ simulations are similar between all systems (Table 1); only **1** is able to significantly induce loop closure.

**Figure 4:**
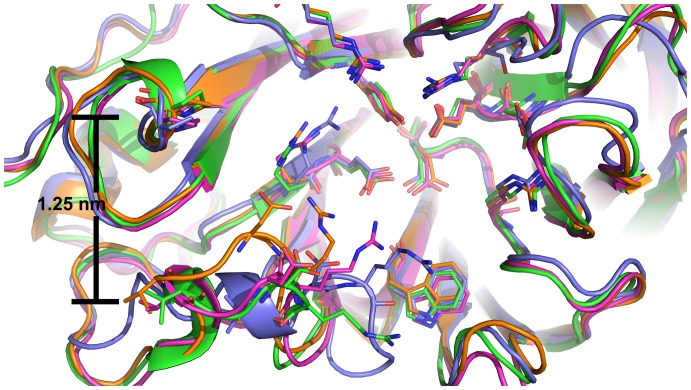
Independence of cavity-width and key residue conformations. Superposition of four MD snapshots of N8_open_-**6** that exhibit a cavity-width of 1.25 nm but differ significantly in conformations of key active site residues, shown as sticks.

Overall, there is no clear trend between ligand positions and cavity-widths and inconsistent relationships between cavity-widths of different NA and ligands. This is at least partly due to the numerous and distinct loop conformations observed for the 150-loop that nevertheless exhibit the same cavity-width, as shown in Figure 4, especially in simulations with 150-binders. Determining which qualify as loop-closed versus loop-open is accordingly ambiguous, as no single distance measurement adequately encapsulates the highly diverse variety of structures observed. It is therefore not possible to determine whether insufficient sampling or measurement uncertainty is responsible for the lack of clear trends based on cavity-widths. In contrast, the clustering analysis described in the following section offers a clearer picture.

### Active Site and Ligand Combined Clustering

**Figure 5:**
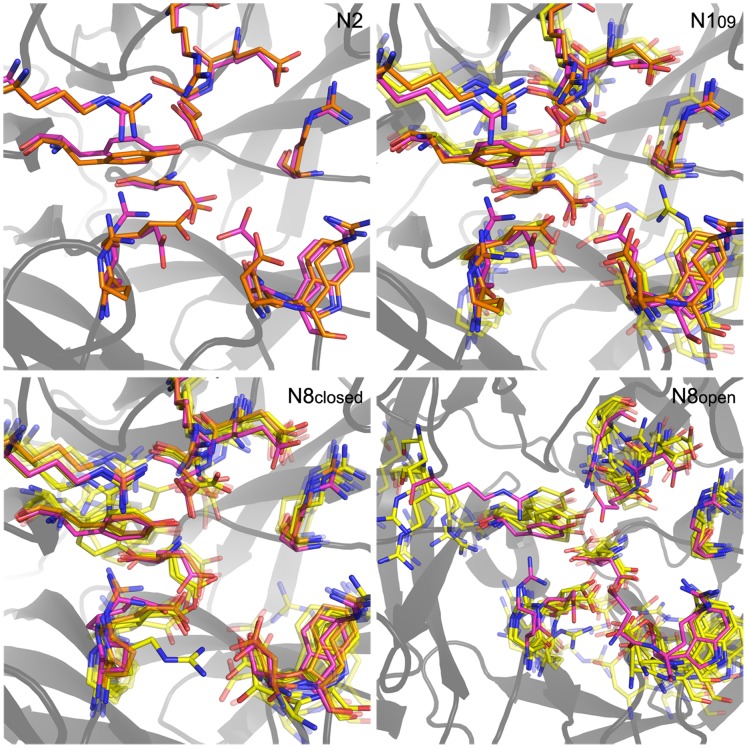
Superimposed active site clusters of all enzymes without ligands. Cluster conformations are depicted in yellow except for the C1 conformations of each enzyme, which are depicted in orange, and the CS conformations, which are depicted in purple. The transparency of each conformation is inversely proportional to its population.

Combined clustering greatly facilitates comparisons between runs of the same enzyme with different ligands. For example, with this method, cluster 1 (C1) for the apo-H3N2 run is identical to C1 for all H3N2-ligand runs (though distinct from C1 of the H1N1 runs). Clusters are numbered sequentially in decreasing order of population over all simulations of that particular enzyme. For all enzymes, the most populated cluster C1 closely resembles the CS, with two minor observed differences. E119 is somewhat twisted from its CS orientation in all enzymes, and R118 is somewhat more recessed in N1_09_ and N8_open_ than in the corresponding CS. These features are evident in Figure 5, which depicts the clusters of the apo-NA simulations for all four enzymes along with the relevant CS for comparison. This illustrates the conformation of each enzyme’s C1 structure, its similarity to the original CS, as well as the populated alternative conformations. Notably, apo-N8_open_ does not spend a significant amount of time in C1, mostly due to R371’s frequent deviation from its CS conformation.

**Figure 6:**
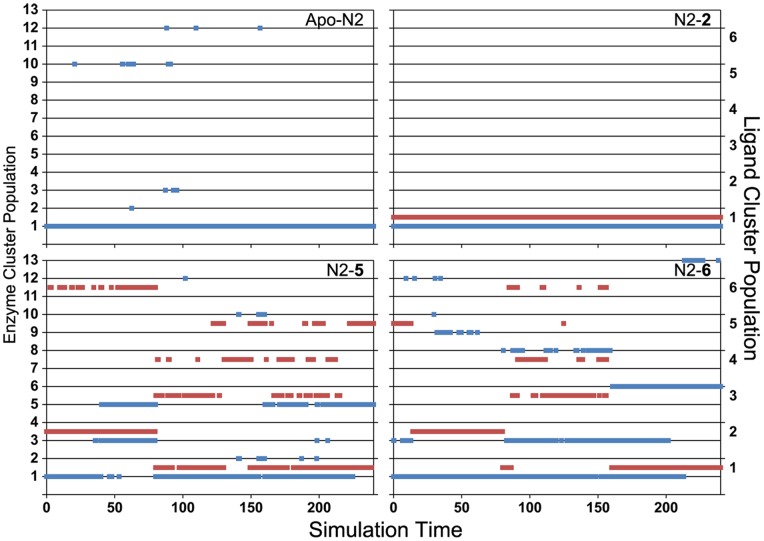
Cluster populations for selected N2 runs over time. Enzyme cluster populations are shown in blue, aligned to the left ordinate scale, and ligand cluster populations are shown in red, aligned to the right ordinate scale.

This approach necessitates clustering ligands separately, which confers the added benefit of separating ligand motions from enzyme motions and thereby facilitating evaluation of their interdependency. By comparing cluster populations, for example, it is possible to determine how a ligand’s various poses are reflected in the conformation of the enzyme. In the text, the results are summarized as follows: enzyme clusters are referred to as C# while ligand clusters are referred to as L#. This analysis results in plots of the conformation of a given enzyme and the conformations of the complexed ligand over time. To illustrate the utility of these results, an example of the resultant plots for four N2 systems is shown in Figure 6 while the others are provided as Text S1.

As can be seen from Figure 6, the stability of apo-N2 is immediately obvious: the enzyme does not significantly deviate from its CS-like conformation (C1). In complex with **2**, N2 demonstrates similar rigidity while the ligand remains consistently in its own CS-like pose (L1). 150-Binders **5** and **6**, by contrast, exhibit multiple conformations and also induce N2 into adopting several alternative conformations. Details of these clusters are given in the following sections, but several features bear noting as they exemplify the versatility of this approach. For one, a particular conformation (C3) is not seen in the apo-N2 simulation or simulations in complex with standard ligands, but is common to both N2–**5** and N2–**7** simulations. Further, there are several clear correlations between enzyme and ligand conformations; for example, N2–**5** exhibits a ligand transition from L2 to L6 while the enzyme transitions from C1 to C3/C5. Determining how ligand conformations interact with enzyme conformations is thus straightforward.

For the sake of brevity, in the following results sections only the clustering results specific to ligands **1, 2, 3, 5,** and **6** will be discussed, which were chosen as representative of all seven ligands. The results for the remaining two ligands are included in Text S2. Only images necessary to highlight important insights are presented below while images of all significant enzyme and ligand clusters are provided as Text S3 and Text S4, respectively. In text, all descriptions of NA and ligand motions are accompanied by the relevant cluster label(s) and percentages in parentheses, which indicate the populations of the specific cluster(s). While each cluster is unique by definition, some motions lead to an excess of clusters that do not differ significantly. For example, when a 150-binder’s sidechain exits the active site and oscillates in solution, several clusters may arise that do not differ in terms of important conformations and interactions. Such groups of clusters are typically described together within the same parentheses. Similarly, when an important motion of an amino acid is common between several clusters, such as R371 withdrawing, these clusters are listed and their populations summed.

### Active Site Conformations in Apo-NA Simulations

The following descriptions correspond to Figure 5. Apo-N2 is extremely static in simulation, as expected, remaining almost entirely in its CS conformation (C1; 99%). By comparison, N1_09_ is far more mobile with significant populations in five clusters and a low C1 population (C1; 34%). These additional conformations stem primarily from changes in the two active site 150-loop residues; D151 can recede (C3; 8%) or advance (C14; 9%); R152 can advance (C5, C14; 39%), fold down onto itself (C18; 4%) or withdraw (C10; 12%). Additionally, Y406 fluctuates significantly and can swing down toward E119 for a stable conformation (C10; 12%).

Apo-N8 is also quite dynamic, exhibiting nine populated conformations in simulations starting from loop-closed and loop-open conformations. In both cases, the residues that contribute to distinct conformations are R118, D151, R152, R292, R371, and Y406. For the N8_closed_ simulations, the CS conformation is rarely adopted (C1; 15%). Instead, R118 is often withdrawn (C4, C5, C9, C17; 42%) but occasionally swings toward D151 (C2; 24%). R371 swings down away from the active site (C4, C6, C15; 14%). R292 frequently moves toward E276 and interacts there (C5, C6, C9, C17; 34%). D151 occasionally recesses (C6, C9, C12, C17; 19%), mutually moves toward R118 (C2; 24%), or extends toward the active site (C15; 3%). R152 generally only fluctuates in place, rarely extending toward the active site (C17; 3%). Y406 is relatively constant, though occasionally extends up toward R292 (10%) or down to R118 (C12; 5%). In N8_open_ simulations, the most populated clusters for the native enzyme differ significantly from the CS, as can be seen in Figure 5, with 0% population of C1. R371 typically swings far from the active site (C12; 12%), D151 and R152 together swing far away from the active site due to backbone loop movements (C18, C23, C26; 15%), and E276 can swing toward R292 (C18, C21; 13%).

### Conformations of NA Active Site and Complexed Standard Binders 1 to 3

**Figure 7:**
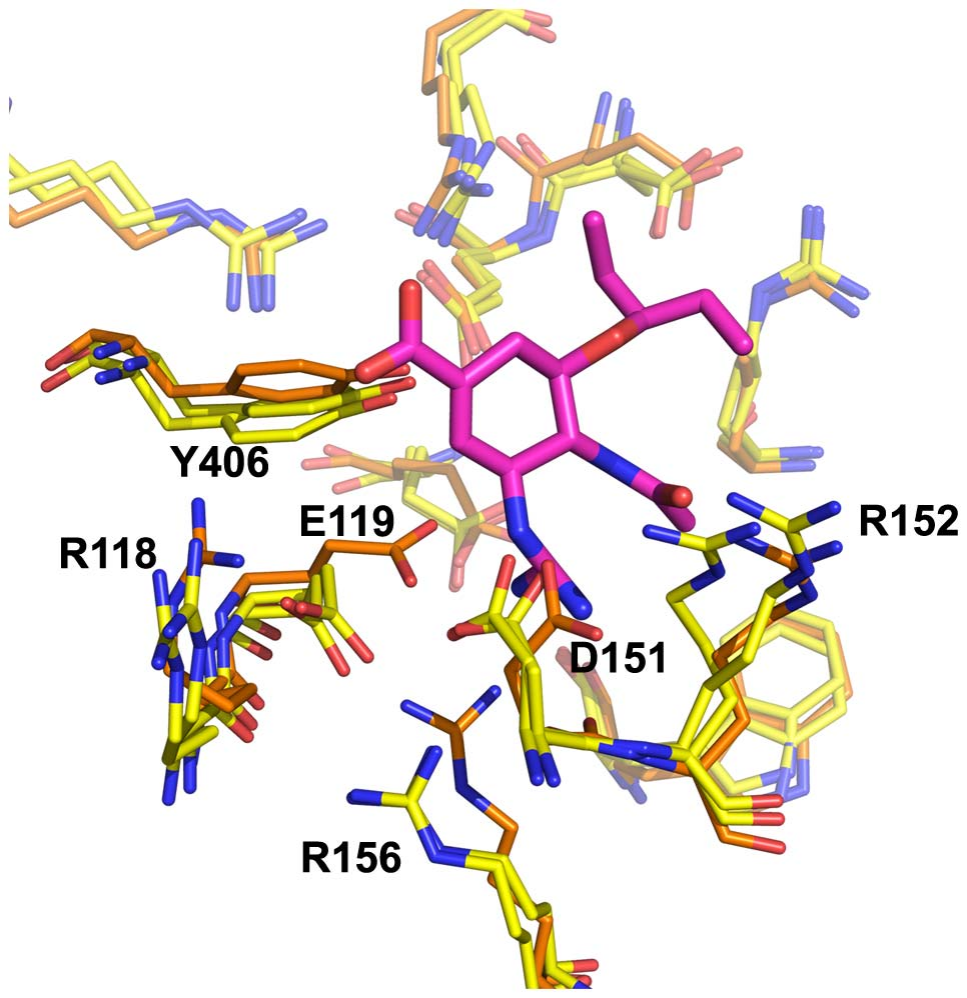
N2-1 alternate enzyme poses. Shown are the C1, C3, C8 of N2 with L1 of **3**. The color scheme is identical to Figure 5.

The N2-**1**, N2-**2**, and the N2–**3** simulations exhibit similar NA stability to the apo simulations; only C1 is highly populated for all three. The ligands also do not stray significantly from the CS poses with 100% populations in L1 for **1** and **2**, and 97% for **3**. However, in one of the triplicate runs with **1**, the ligand’s guanidinium moiety encourages R156 to withdraw, causing E119 to follow and bend away from the active site. R118 simultaneously withdraws somewhat as D151 bends toward the guanidinium as well, and R152 swings toward the ligand’s amide (C3, C8; 13%), as shown in Figure 7. Similar changes are evident in most simulations with the guanidinium-containing ligands, **1** and **3**.

With N1_09_, the standard binders greatly reduce the mobility seen in the apo simulation, yielding C1 populations increased from 34% to 96%, 100%, and 93% for **1**, **2**, and **3**, respectively. The conformation of **2** is essentially static, exhibiting a crystal-structure pose for 94% of the simulation (L1) with a slight twist of its pentyloxy chain for 4% of the simulation (L2). Similarly, **1** occupies its L1 conformation for 84% of the simulation, with the glycerol chain twisting for 15% of the simulation (L2) toward R292 rather than R224. No other contacts are lost in this pose. R152 also rarely swings out of the active site (C17; 3%).

**Figure 8:**
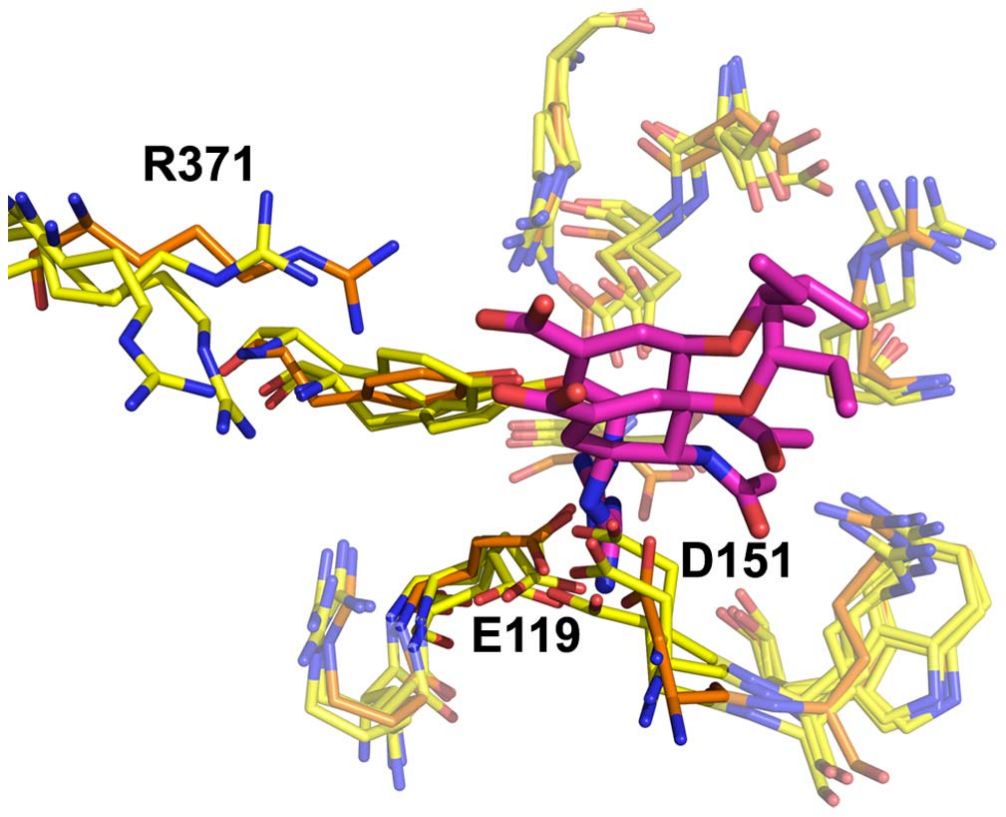
N1_09_-3 dominant pose and its destabilization. Shown is C1, C15, C25 and C28 of N1_09_ and L1 and L5 of **3**. The color scheme is identical to Figure 5.

In contrast, **3** seldom occupies its most CS-like conformation in complex with N1_09_ (L3; 8%). In its dominant cluster (L1; 70%), the guanidinium group moves closer to the center of the active site, greatly reducing the distances to electronegative Y406, E277, and the alpha-carbonyl of W178. This pose, shown in Figure 8, does not significantly alter the carboxylate-arginine interactions and the enzyme typical remains in C1 (93%). However, the enzyme and ligand frequently undergo a simultaneous transition where R371 withdraws (C15, C25, C28; 7%) and the ligand destabilizes somewhat (L2, L5; 9%), as shown in Figure 8. This conformation is similar to L1 and L3 though the carboxylate is now outside the active site. This behavior is likely not seen in the N2–**3** simulations due to the greater rigidity of R371 and is the only example of mobility of R371 in N1_09_ simulations, suggesting that the ligand exerts a significant effect on R371.

In simulations of N8 with the commercial ligands **1** and **2**, enzyme motions are again dramatically reduced in comparison to the apo simulations. This results in increased C1 populations from 15% of apo-N8_closed_ to 100% for both N8_closed_-**1** and N8_closed_-**2**, and from 0% in apo-N8_open_ to 82% and 89% for N8_open_-**1** and N8_open_-**2** C1 populations, respectively. In all cases, the ligand rarely deviates from the CS-like pose L1, except for minor rotations of the pentyloxy/glycerol groups. The N8_open_ **-1** enzyme also occasionally occupies two similar clusters in which R118 and D151 are somewhat extended toward the ligand and E276 has withdrawn from R224 (C6, C24; 17%). The N8_open_ **-2** runs exhibit one major enzyme motion, which is R118 swinging down toward D151, with the latter moving toward it as well (C8, C36; 10%).

**Figure 9:**
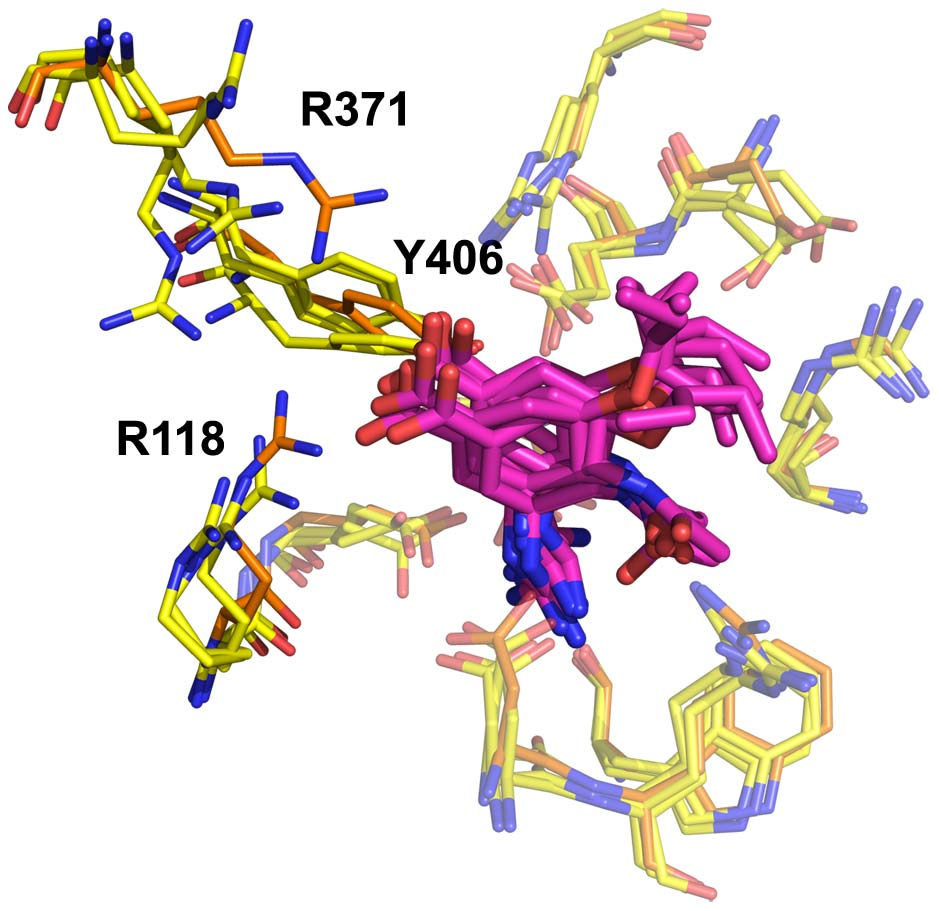
N8_closed_-3 ligand oscillations and R371 mobility. Shown is C1, C4, C14 and C18 of N8_closed_ and L1, L3, L4, L6, L8, and L9 of **3**. The color scheme is identical to Figure 5.

In contrast to **1** and **2**, significant enzyme dynamics are again observed in the N8_closed_-**3** simulations (C1; 66%) where motions in R118, R371, and R292 give rise to five populated enzyme clusters. R118 frequently recedes to resemble a type-2 CS (C4, C14, C18, 21%), twice as often as seen in the apo-N8_closed_ simulation, while R371 swings away from the ligand (Figure 9). These swings also occur without further changes to the active site (C6; 11%) and are similar to the movements observed with N1_09_-**3** (Figure 8), further supporting the destabilizing effects of **3** on R371. Simultaneously, **3** fluctuates within the active site and its carboxylate group frequently drifts away from R371, encouraging R118 to recess and allowing R371 to swing freely (L1, L3, L4, L6, L8, L9; 72%). Without the strong R371 interactions, the ligand’s carboxylate can then readily swing out of the active site (L2, L5, L7; 25%). Nevertheless, the enzyme is significantly more CS-like than the native enzyme overall, suggesting that **3** stabilizes C1 but to a lesser extent than **1** and **2**.

N8_open_-**3** exhibits similar patterns as N8_closed_-**3**, with R371 frequently swung far back (C4; 28%) or R292 and E277 swung toward E276 (C5; 28%). This is likely due to the position of **3** which exhibits poses that are similarly sunken as those observed in N8_closed_ simulations (L1, L2, L4, L5; 91%). There is significant movement of the ligand’s carboxylate group, which occasionally exits the active site (L2; 17%). Only during one triplicate does the ligand adopt the CS-like pose and only infrequently (L3; 6%), but during this time the enzyme rarely has R371 withdrawn (C4; 10%), demonstrating a correlation between these changes.

### Conformations of NA Active Site and Complexed 150-Binders 5 and 6

**Figure 10:**
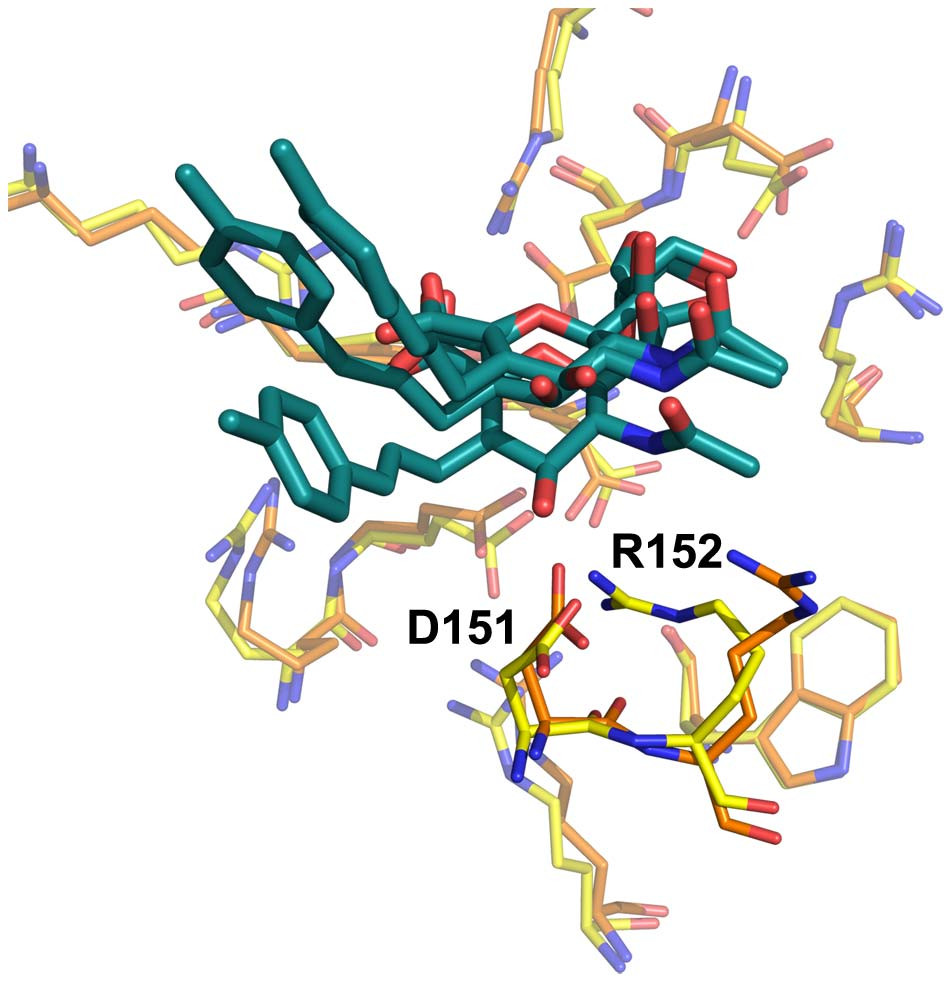
N2–5 facilitating R152 moving into the active site center. Shown is C1 and C5 of N2 and L1, L3, and L4 of **5**. The color scheme is identical to Figure 5.

The 150-binders are far more dynamic than the standard binders, especially in regards to their sidechains. Core interactions such as carboxylate-arginine interactions are typically preserved, however. As such, in complex with N2, **5** maintains a CS-like pose for one of the triplicate runs but with its phenyl-sidechain oscillating in (L7; 5%) and out (L2, L6; 29%) of the active site. For the remaining two triplicates, only the ligand’s glycerol and carboxylate moieties remain in the active site (L1, L3, L4, L5; 41.5%). Without significant correlation to the ligand pose, the enzyme generally adopts either a CS-like pose (C1, 67%) or an alternative conformation (C5; 25%) in which R152 is swung down into the active site (Figure 10). This movement is facilitated by contacts between the ligand’s hydroxyl and amide, and then stabilized by electronegative carboxylate and carbonyl moieties within the active site interior. Once formed, it then supports the ejection of the ligand as is observed several times during the trajectory. One additional enzyme pose, arising for 18% of the time in which the ligand is CS-like is C3, where R118 and E119 move to interact with the ligand’s phenyl sidechain, and R152 swings toward the ligand hydroxyl.

**Figure 11:**
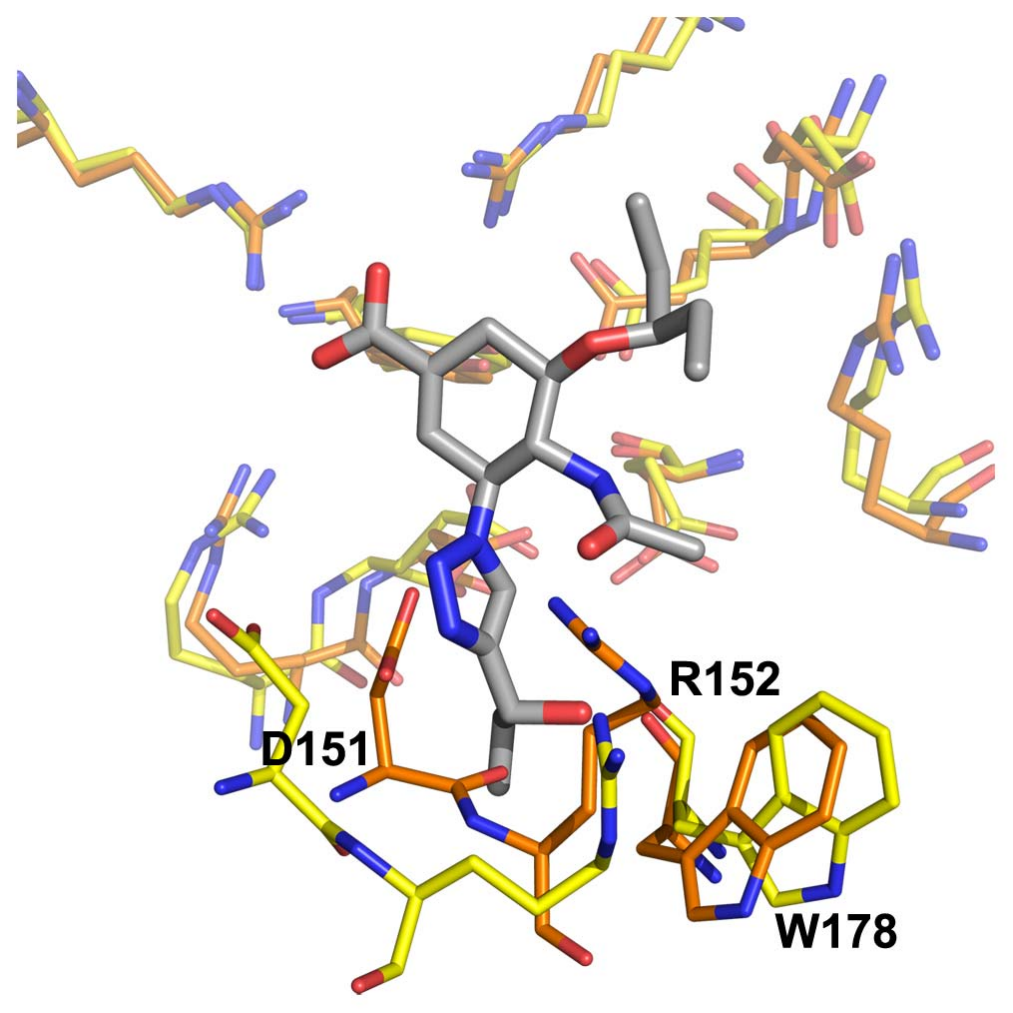
N2–6 demonstrating a recessed D151–R152. Shown is C1 and C6 of N2 and L1 of **6**. The color scheme is identical to Figure 5.

In N2–**6** runs, the ligand never adopts the expected pose though remains within the active site for roughly one third of the simulation time. The most CS-like poses (L1, L3, L4, L6; 60%) differ in that the sidechain is angled directly under the D151 and R152 residues, rather than adjacent to them. This binding mode is similar to the sunken poses of ligand **3**. While the ligand is alternating between these flexible poses, it frequently loses its carboxylate interactions (L3, L4, L6; 42%) while the enzyme mainly resides in C1 (40%). Occasionally, D151 and R152 shift to either side of the ligand’s triazole (C3; 19%), but more frequently, these residues swing back (C6; 37%), as shown in Figure 11. This ligand is thus able to force open the loop residues of the rigid N2 better than any other studied ligand, but is prone to alternative binding poses. During the remaining 40% of the simulation, the ligand is somewhat ejected from the confined enzyme active site; only its carboxylate and sidechain remain within the active site (L2, L5; 31%) and the enzyme is dominantly in C1 (98%).

During the N1_09_-**5** simulation, the enzyme is constantly in a C1 pose (98%). The ligand is almost always in a CS-like pose (L1; 66%) with its sidechain directed into the 150-cavity, suggesting these conformations are not incompatible. The remaining simulation time has the sidechain fluctuating in the solution without an apparent impact on the enzyme.

**Figure 12:**
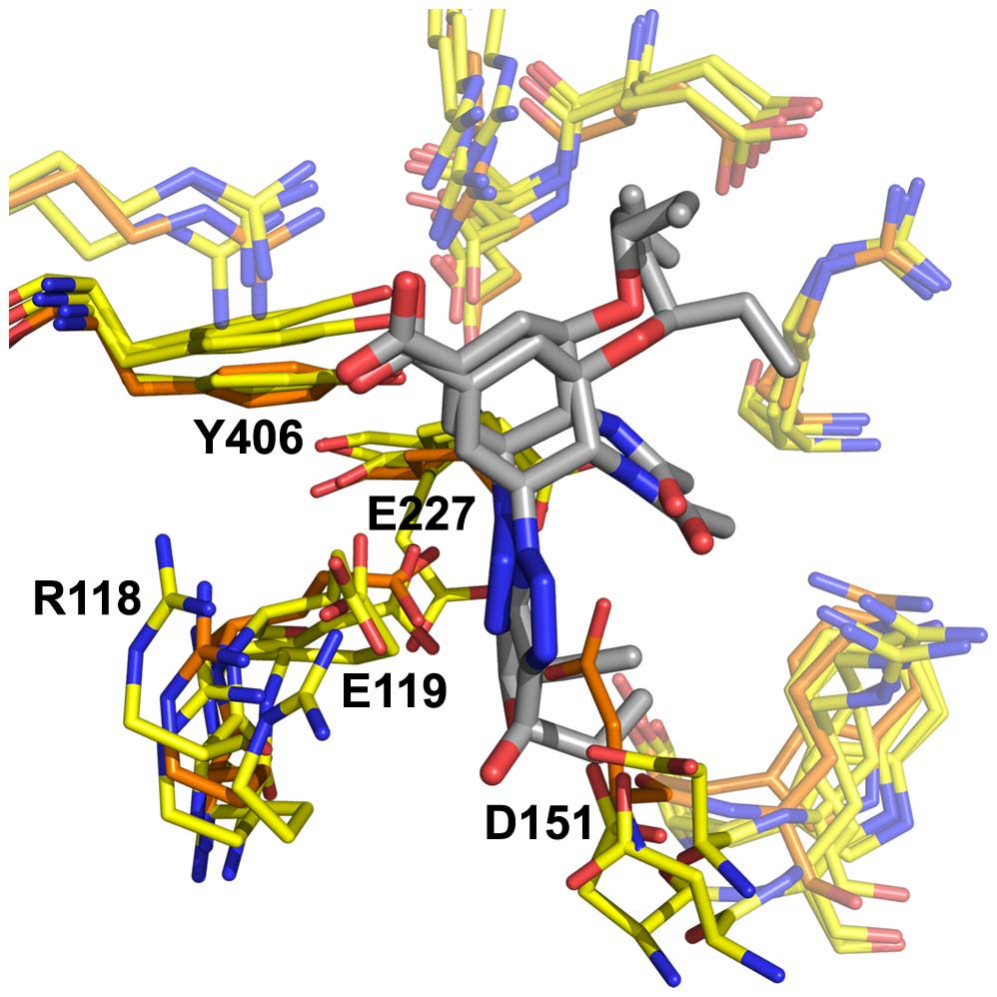
N1_09_-6 shows a significant loop-opening effect. Shown is C3, C7 and C9 of N1_09_ and L1 and L2 of **6**. C1 is included for reference, though not occupied. The color scheme is identical to Figure 5.

In contrast, during simulations of N1_09_-**6** C1 is never occupied, mostly due to variation in 150-loop residue D151 and nearby amino acids (Figure 12). **6** adopts a CS-like pose for two of the triplicate runs (L1, L2; 62%). In this pose, its electronegative triazole group pushes back the electronegative D151 into the solution, which thereafter interacts with the ligand’s sidechain hydroxyl group (C3, C7, C9; 90%). This is similar to the conformations of N2–**6** with one exception; the recessed 150-loop residues now adopt multiple distinct orientations. E119 and occasionally R118 also interact with the ligand’s sidechain. The ligand’s sidechain fluctuates outside of the active site during the remaining triplicate although the key interactions remain intact and the loop remains recessed (C3, C6, C9; 98%). Frequently, Y406 moves forward to fill the gap (C6; 68%).

**Figure 13:**
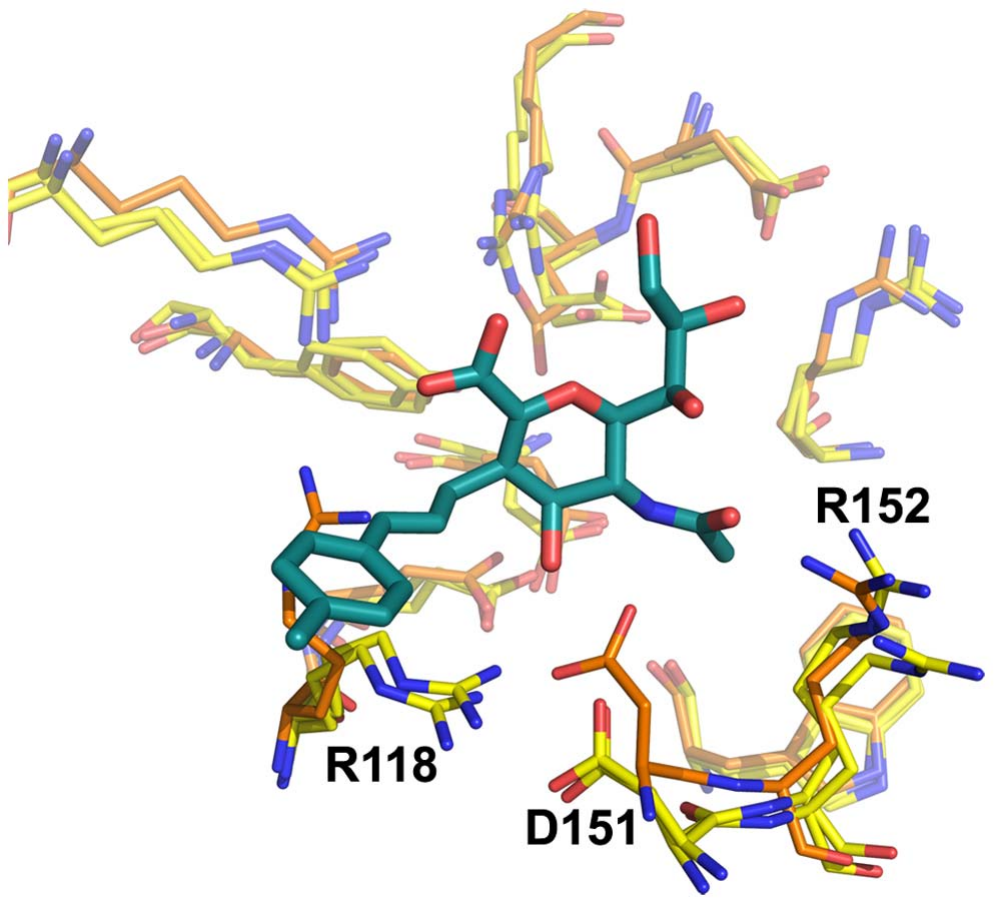
5 is blocked from reentering the active site of N8_closed_. Shown is C2 and C13 of N8_closed_ and L3 **5**. C1 is included for reference. The color scheme is identical to Figure 5.

In complex with N8_closed_, **5** adopts multiple poses, all with the key carboxylate interactions preserved. Only residues R118, D151, and R152 exhibit different poses. While the ligand most resembles the CS-like pose (L2, L6, L7; 32%), the enzyme is typically in C1 (78%). However, the ligand’s sidechain frequently clashes with D151, causing it to swing back, while R118 swings in and interacts with the aromatic sidechain and R152 either remains in its normal position (C3, 16%) or withdraws (C8, 10%). In another populated pose, the glycerol chain contacts are lost and the sidechain enters the solution (L1; 30%) while D151 is periodically pushed away from the sidechain toward R152 (C7; 4%). In a third pose (L3, 23%), all contacts are maintained although the side chain exits the active site and R118 again swings in toward D151 as is seen with apo-N1_09_ simulations (C2, C13; 70%). This interaction (Figure 13) prevents the ligand from re-entering the 150-cavity, as is observable in the trajectory.

With N8_closed_, **6** often adopts a CS pose with its sidechain frequently entering the 150-cavity (L1, L4, L7; 60%) while the enzyme fluctuates between several conformations. Most interestingly, the enzyme adopts a C1 conformation for 91% of the time in which the ligand’s sidechain exits the active site, but only 44% otherwise, demonstrating the sidechain’s impact. Instead, D151 is often pushed far back (C3, C8; 41%) or turned toward R152 (C7; 13%), which occasionally swings in to interact with the ligand’s hydroxyl group along with E119 (C8; 6%).

With N8_open_, **5** is considerably more dynamic, giving rise to 13 clusters with more than 1.0% occupation. The trajectory can be divided into two groups; one in which the enzyme changes conformation to accommodate the ligand, and the other in which the reverse occurs. The former is constant for one of the triplicates – the ligand adopts a CS-like pose (L1; 33%) during which time the enzyme is predominantly in C3 and C28 (98%). In these poses, D151 is swung toward R152 while R371, E276, and E277 are withdrawn, demonstrating successful blocking of the loop by the ligand. In the remaining two triplicate runs, the ligand is largely outside of the active site (L2–L13; 94%) with only the carboxylate interaction intact. The enzyme meanwhile adopts a CS-like pose (C1, C6, C22; 94%) with minor movement in R118. Occasionally the loop recedes somewhat (C5; 4%).

**Figure 14:**
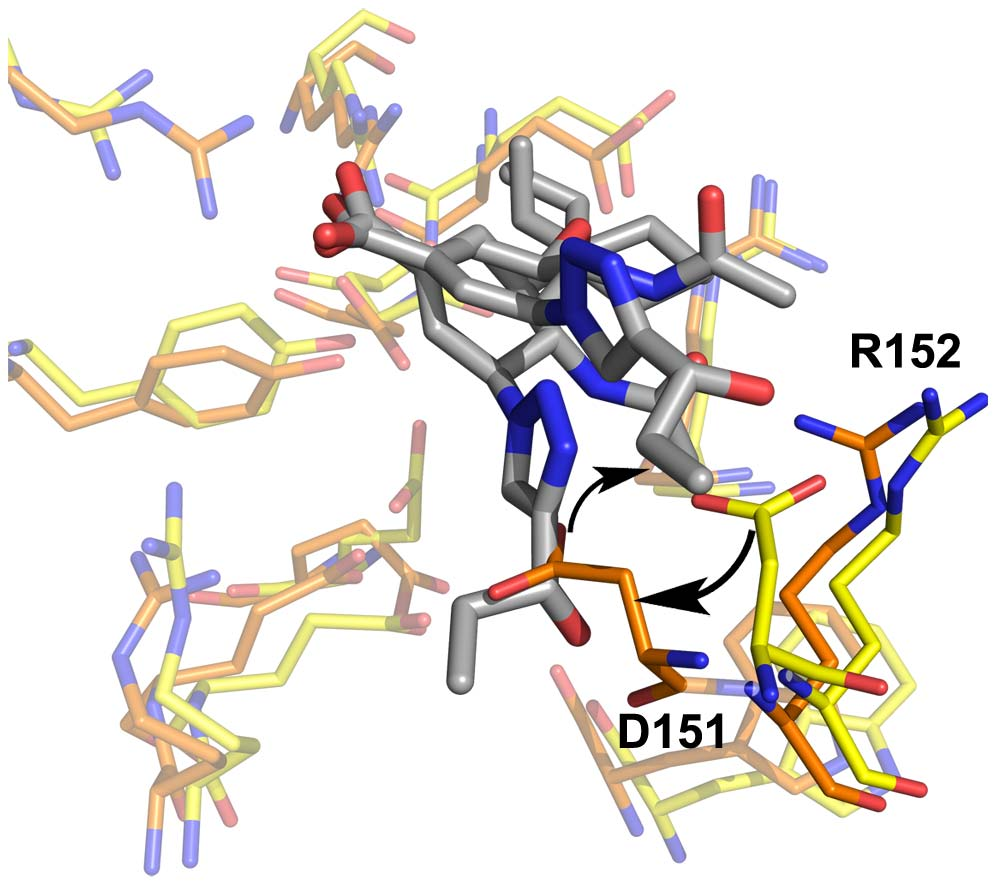
Simultaneous 150-loop closure and ligand sidechain ejection for N8_open_-6. Shown is C1 and C3 of N8_open_ with L1 and L2 of **6**. The color scheme is identical to Figure 5.

In simulation with N8_open_, **6** adopts a CS-like pose for the beginning of all three triplicates (L1; 50%) while the enzyme’s 150-loop is forced open (C2, C3, C13, C14, C19; 91%). Occasionally R118 swings in toward the ligand’s triazole moiety (C14; 4%), remaining as stable arrangement for one entire triplicate. In another triplicate, the ligand exits into the solution while the loop continues to oscillate, and in the remaining triplicate the loop closes (C1; 8.3%) and thereby displaces the ligand’s sidechain (L5; 5%), shown in Figure 14. These runs demonstrate that **6** can be stable within the active site, or with its sidechain directed into the solvent, while the loop fluctuates or rests in a closed conformation.

## Discussion and Conclusions

The above discussion detailed the motions and binding modes observed throughout the simulations, and several key results from the clustering analysis are summarized in Table 2. This table lists the populations of the CS-like C1 populations, summed populations of every loop-closed conformation for each enzyme, the summed population of ligand conformations in which the ligand remains within the active site, as well as the latter’s impact on loop closure. Specifically, the total populations of loop-closed conformations that occur *while* the cavity is fully occupied are given. By comparing the propensity of an enzyme’s 150-loop to close in the apo state, versus when in complex with a specific ligand, and further when that ligand is fully within the active site, the ligand’s influence on the loop can be better quantified. For example, in complex with **4**, N1_09_ exhibits a reduced loop-closed population of 39% relative to 77% of the apo simulation. However, during the 30% of the simulations in which **4** is entirely within the active site, the loop-closed population increases to 85%. This reveals that the N1_09_-**4** complex is far more likely to exhibit a closed 150-loop when the ligand is within the active site than when the ligand is partially exited, contradicting the expected effects of these 150-binders of forcing open the 150-loop by occupying the 150-cavity [6]. Instead, it appears that it is the fluctuations of **4** that encourage the 150-loop of N1_09_ to open.

**Table 2:** Clustering results summary by neuramindase.

NA	Ligand	CS-like Pop.	Loop Closed Population^a^		Ligand in Active Site Population^a^	Populated Conformations^a^	
			Overall	While Ligand is in Active Site^b^		Enzyme	Ligand
**N2**	**Apo-**	99%	99%	–	–	1	–
	**1**	86%	100%	100%	100%	2	1
	**2**	100%	100%	100%	100%	1	1
	**3**	98%	98%	98%	100%	1	2
	**4**	63%	93%	–	0%	2	2
	**5**	67%	98%	100%	33%	2	9
	**6**	58%	77%	37%	35%	2	6
	**7**	42%	42%	42%	100%	3	2
**N1_09_**	**Apo-**	35%	77%	–	–	3	–
	**1**	97%	99%	99%	100%	2	2
	**2**	100%	100%	100%	100%	1	3
	**3**	94%	98%	98%	96%	1	5
	**4**	32%	39%	85%	30%	3	7
	**5**	98%	98%	100%	67%	1	4
	**6**	1%	1%	0%	69%	3	6
	**7**	5%	8%	7%	52%	2	6
**N8_closed_**	**Apo-**	16%	42%	–	–	4	–
	**1**	100%	100%	100%	100%	1	1
	**2**	100%	100%	100%	100%	1	2
	**3**	66%	73%	74%	93%	3	8
	**4**	91%	92%	91%	66%	2	7
	**5**	56%	59%	77%	64%	4	6
	**6**	59%	59%	48%	75%	3	10
	**7**	60%	60%	49%	71%	2	6
**N8_open_**	**Apo-**	0%	22%	–	–	5	–
	**1**	83%	100%	100%	100%	2	2
	**2**	90%	100%	100%	99%	2	2
	**3**	41%	42%	42%	98%	2	5
	**4**	51%	84%	85%	57%	4	5
	**5**	47%	60%	7%	36%	3	9
	**6**	8%	10%	2%	65%	4	9
	**7**	0%	18%	0%	6%	3	11

a See materials and methods for criteria.

b Percentage of simulation time that a loop-closed cluster is occupied while the ligand remains in the active site.

In the absence of any ligand, the enzymes vary significantly in mobility. The C1 population of N2 is nearly 100%, dropping to 35% for N1_09_, and further to 16% and 0% for N8_closed_ and N8_open_, respectively. These conformational changes are not purely derived from loop mobility though; N1_09_ adopts loop-closed conformations for 77% of the simulations, indicating that the majority of its mobility is due to fluctuations in amino acids that are not part of the 150-loop. Similarly, N8_closed_ and N8_open_ remain loop-closed for 37% and 18% of the apo simulations, respectively, suggesting that roughly half of their conformational flexibility is due to loop movement. These closed-loop values are similar to those determined by cavity-width monitoring, supporting the validity of this measurement for apo systems. However, there is little correlation between loop-closed conformation populations and cavity-width for the simulations without complexed ligands.

Regarding ligand dynamics, the standard binders are almost entirely stable within the active site, as demonstrated by the average values for each ligand given in Table 2. 150-Binders, by contrast, remain within the active site less often, yielding averages from 38% for **4** to 61% for **6**, likely due to their lower binding affinities. In fact, the trend of these average active site populations matches the known potency of these compounds: **1**≈**2**≈**3**>**6**>**7**>**5**>**4** [6], [7]. In general, measuring cavity-occupation by inspection of cluster structures yields better results than COM-distances, which are less able to discern ligand fluctuations and reorientations.

In line with their stability, the conformations of standard binders **1** and **2** are extremely static, as has been reported previously. Only twists of the pentyloxy and glycerol chains are observed. Ligand **3**, despite its similar structure, potency, and active site occupancy to **1** and **2**, is notably more dynamic. It averages 4.8 populated conformations – several more than **1** or **2** – while stabilizing the closed-loop conformations less and inducing an unexpected mobility of R371. It is surprising that, despite the frequent loss of the energetically important carboxylate-R371 interactions [4], this inhibitor exhibits comparable potency to **1** and **2** [7]. This could be due to the viability of alternative poses for **3**, which were observed in multiple simulations and are shown in Figures 8 and 9. This flexibility may also be responsible for the unreduced potency of **3** against oseltamivir-resistant influenza strains with the H274Y mutation [7], which reportedly target the pentyloxy group of **2** that is common to **3** [20]. Given that the potency of **1** is similarly not reduced by the H274Y mutation [20], it is possible that the common guanidinium moiety is a factor.

Unlike the standard binders, the 150-binders do not reduce the mobility of the enzyme, exhibiting similar populated enzyme conformations relative to apo simulations. However, 150-binders **4** and particularly **5** increase the population of the CS-like conformations relative to the apo simulations, much like the standard binders. 150-Binders **6** and **7**, by contrast, do not significantly alter the CS-like conformation populations but do significantly increase the tendency of NA to adopt loop-open conformations. This effect is significantly pronounced for compound **6** when considering only the period in which the ligand is fully within the active site, indicating that its relative instability reduces its capacity to lock open the 150-loop. The six other ligands, in contrast, did not demonstrate significantly different behavior overall when only considering the periods in which they remain entirely within the active site. Overall, compound **6** is most representative of a successful 150-binder, exhibiting a relatively high active site population (61%), an average decrease of loop-closed populations of 37% relative to apo simulations, and a greater reduction of loop-closed populations (20%) when fully within the active site.

On the importance of starting conformations, as demonstrated by comparing N8_closed_ and N8_open_, it is clear that the position of the 150-loop exerts a significant effect on the behavior of the NA and the ligands. There is greater mobility observed for N8_open_, which exhibits more significantly populated conformations and decreased C1 and loop-closed populations than N8_closed_. These effects are reduced in simulations of the standard binders, which generally equilibrate quickly and exhibit greater stabilities. The 150-binders, in contrast, are more likely to exit the active site during the N8_open_ simulations – especially **7**, which features a sidechain that is relatively stable in solution (Text S2). Generally, these results demonstrate that triplicate simulations of 100 ns are insufficient for overcoming starting-conformation bias for all simulations except those with standard binders. This is confirmed by analyzing the clustering analysis results as 20 ns blocks (Text S5), which suggests that cluster populations are generally stable over the length of the simulation after removing the initial 20 ns. This suggests that in order to overcome the bias of the beginning orientation of the 150-loop, removing more than 20 ns – at least 100 ns – from the beginning of simulations prior to analysis would be necessary. This would require simulations of much greater length than 100 ns, at least for the mobile N8 systems, if both loop-closed and loop-open structures are not employed. It is unclear whether the more stable N1_09_ and N2 simulations are equally sensitive to starting loop-orientations, and therefore whether prior analyses of the free energy of loop closure are reasonable [21].

In comparison to cavity-width monitoring and COM measurements, combined clustering offers clearer trends between ligand positions, the 150-loop’s conformation, and NA fluctuations, which we attribute to several factors. For one, the cavity-width appears to require more sampling overall. This is evidenced by the consistency of the results for the apo systems, which equilibrate faster, in comparison to the holo systems, which lack clear trends. Secondly, the variety of loop positions cannot be simplified to a single distance measurement as argued above (Figure 3). Thirdly, COM measurements are ambiguous, being unable to distinguish between a variety of ligand positions that may share the same COM, for example. Finally, combined clustering is far better able to analyze shared conformations between diverse systems, which allows for straightforward tabulation of CS-like populations, loop-closed conformations, and enzyme mobility.

Another common measurement of mobility, root mean squared fluctuation (RMSF), was also evaluated. However, in general, these values offer less insight than the number of populated conformations available from clustering. The average apo NA adopts seven significantly populated conformations, dropping to five when in complex with 150-binders, and two when in complex with standard inhibitors. In contrast, RMSF values (Text S6) attest to the stability of N2 and the mobility of N8, relative to N1_09_, but otherwise show few clear trends between the various complexes. This is because RMSF values cannot distinguish between a residue that generally only oscillates rapidly in place, such as Y406, from a residue that adopts multiple conformations, such as D151.

Overall, this work highlights the extremely complex and dynamic interactions of influenza neuraminidase with its inhibitors. This is particularly true for the 150-binders studied, which are more prone to exiting the active site and are more dynamic than the standard binders. None of these ligands demonstrated all of the desired characteristics of this class, though compound **6** is nearest. In general, detailed analysis is required to discern the impact of ligand mobility, which is generally deleterious for the 150-binders but can be beneficial, as for compound **3**. Similarly, interesting phenomena occur frequently that are entirely dynamic in nature, such as partially exited ligands encouraging the NA to adopt unique poses that in turn prevent the ligand from reentering the active site (ligand **5** in particular). Future design of 150-binders will require significant consideration of such dynamics, which are readily probed by MD simulations and clustering techniques. The results of combined clustering in particular can be efficiently analyzed to determine the interdependency of given conformational changes, such as the relation between loop-closed populations and active site occupation. Cavity-width monitoring, in contrast, appears well suited for apo systems but does not give meaningful results for holo simulations. Additionally, molecular docking approaches may better account for NA dynamics by including a greater variety of NA conformations, such as the cluster-centroid structures of this work (provided as Structures S1).

One of the central challenges of improving the potency of 150-binders is that increased loop-open conformation populations are associated with ligands increasingly exiting the active site, as evidenced by decreased active site populations for compounds **4**–**7** in complex with N8_open_ versus N8_closed_. This is likely related to a decrease in binding affinity. Although our results confirm the relative stability of the loop-open state for the typical group-1 N8 systems, in the absence of binding ligands, loop closure is certainly an energetically favorable process in the presence of the strongest inhibitors, **1** and **2**. Therefore, the ideal 150-binders may similarly induce loop closure by maintaining contacts with the 150-loop residues, while also forming additional contacts within the 150-cavity. Our results indicate that such conformations are possible.

## Materials and Methods

### System Preparation

All systems were prepared in a standardized fashion to allow for convenient automation via scripting. For each individual protein and ligand, properly aligned, parameterized, and processed structure files were first produced. These structure files were then combined to produce all complexes, prior to simulation. All protein structures (2AEP, 3NSS, 2HT7, 2HT8) were first validated and repaired with MolProbity [22]. The ‘A’ chain of each was extracted and uploaded to the PDB2PQR server [23], in which histidine residues were protonated at pH 6.5 and verified manually. Disulfide linkages were enforced with the proper AMBER notation and the resultant files were input into tleap of Amber 12.0 [24], automatically renumbered, then exported as pdb files. All structures were then imported into PyMOL and aligned [25]. Crystallographic water molecules from all CS that did not clash with any of the ligand-enzyme complexes were combined and added to each structure, along with the key calcium ion if absent [26], and the structure was output. Each ligand was constructed and subjected to at least one dozen optimization calculations, at the Hartree-Fock level using the 6-31G* basis set, from different conformations in Gaussian 09 [27]. RESP charges [28] for each unique conformation were then derived using multiple-orientations from single point calculations input to the R.E.D. server [29]. Slight variations in atomic positions yielded charges that varied significantly, and therefore the set of charges that were most consistent among and between the ligands was selected (Text S7). All ligands were then docked into 2HT7 using AutoDock Vina [30]. As the top-scoring poses did not consistently agree with crystallographic data, the poses for each ligand were selected manually. For ligands **1**, **2**, **4**, and **5**, those that matched most closely to the PDB structures 2HTQ, 2HT8, 309J, and 309 K, respectively, were selected. For ligands **3**, **6**, and **7**, the poses were similarly chosen by comparison to unpublished crystallographic data for each ligand, yielding similar binding modes to the other ligands in all cases. Each ligand’s pose was then combined with the relevant atomic charges described above to produce Tripos mol2 files. Complexes of all ligands and enzymes were then produced via combination in tleap. All proteins were then parameterized using Amber ff99SB, and all ligands were parameterized with the General Amber Force Field (GAFF) [31] with the corresponding RESP charges, using tleap and antechamber. AMBER files were then exported.

### Molecular Dynamics Simulations

MD simulations of all systems were conducted with the GROMACS suite, version 4.5.4 [32], utilizing the Amber ff99SB force field [33]. AMBER files for each complex were converted into GROMACS formats using ACPYPE with the “gmx45” option [34]. Each system was placed in a dodecahedral box with a minimal 12 Å distance between solute and box edge and solvated with TIP3P water molecules. Salt ions were then introduced to achieve a concentration of 0.15 M and neutralize the overall charge. Each system was then treated to at least two alternating rounds of 5000 steps of steepest descent and conjugate gradient minimization. Following minimization, random velocities were generated in the first step of equilibration to yield unique triplicate runs of each system. Equilibration entailed first gradually heating the system from 0 K to 300 K in 60 K increments with a Berendsen thermostat during simulations of 40 ps duration. Position restraints on solute molecules began at 1000 kJ mol–1 nm–2 and were reduced by 200 kJ mol–1 nm–2 per incremental run. Pressure was then equilibrated during three steps. First, with 200 kJ mol^–1^ nm^–2^ solute restraints, a Berendsen barostat with a time constant of 2.0 ps and a reference pressure of 1.0 atm, and a Berendsen thermostat with a time constant of 0.5 ps and a reference temperature of 298 K. After 100 ps, position restraints were removed for an additional 100 ps simulation. Finally, the pressure and heat controls were changed to a Nose-Hoover [35] thermostat and a Parrinello-Raham barostat [36], and the system was equilibrated for a final 400 ps before beginning production runs with the same configuration. Throughout, the LINCS algorithm was used to constrain bonds involving hydrogen atoms and the leapfrog integrator was employed with a 2 fs time step. Short-range interactions were calculated with a cut-off of 1.0 nm for columbic interactions and 1.3 nm for van der Waals interactions. Long-range electrostatic interactions were calculated with the particle mesh Ewald (PME) algorithm using a grid spacing of 0.12 nm and an interpolation order of 4. Neighbor lists with a 1.0 nm cutoff were updated every 5 steps.

### Trajectory Analysis

RMSD plots of all heavy protein atoms (Text S8) and all heavy active site atoms (Text S9) of all systems illustrated general convergence after no longer than 20 ns of post-equilibration simulation time. Accordingly, the first 20 ns of all trajectories were discarded and the three triplicate runs were concatenated prior to analysis, yielding 240 ns of fully equilibrated simulation time for each of the 28 systems. All systems were aligned based on the alpha carbons of the 100 NA residues with the lowest RMSF values. Cavity-width was measured using the GROMACS suite as the minimal distance between the alpha-carbon of residue 431 and the sidechain carbons of residue 149 [4], [11]. Centre of mass distances between ligands and enzymes were measured from all non-hydrogen ligand and enzyme atoms. Combined clustering was performed by extracting the enzyme trajectories from all systems, then renumbering residues and atoms to ensure that all numberings of apo and holo systems of the same enzyme matched. All trajectories of the same enzyme were then concatenated. A 38401 by 38401 RMSD matrix for the combined trajectories of each was then calculated from a time step of 50 ps and based on the sidechain heavy atoms of key, conserved active site residues: R118, E119, D151, R152, W178, R224, E227, E276, E277, R292, R371 and Y406. Clustering was then performed on each combined enzyme trajectory using the Gromos algorithm [37] as implemented in the GROMACS package with a RMSD cut-off of 0.12 nm. This cut-off was chosen as the optimal balance between the number of clusters and their meaningfulness, after experimenting with values from 0.10 to 0.25 nm. Comparison of the cluster centroids to the averaged cluster structures allowed confirmed that the centroid structure was representative of all relevant frames. Ligands were clustered separately by the same method, although based on all heavy atoms and a RMSD cut-off of 0.13 nm. Results for the enzymes were separated and sorted via custom Python scripts and assembled along with the ligand clusters in spreadsheets to facilitate interpretation. To assemble the information in Table 2, NA clusters were deemed to be “loop-open” if the 150-loop residues D151 and R152 met two criteria. First, the RMSD difference between D151 and R152 of the cluster in question and of the most CS-like cluster was greater than 0.450 Å. Secondly, the distance between the terminal oxygen atom of Y406 of the most CS-like cluster and the carboxylate carbon of D151 of the cluster in question was greater than 8.50 Å, or the distance from the same oxygen to the arginine carbon of R152 of the cluster in question was greater than 10.80 Å. Similarly, ligand poses were deemed to be within the active site if the majority of their atoms were within the active site cavity, regardless of conformation. However, if sidechains of the 150-binders were directed into the solvent, the pose was deemed to not be within the active site as the sidechain occupying the 150-cavity is an essential requirement of 150-binders. Structures of the clusters are provided as Structures S1. The average values in Table 3 were generated by averaging all individual runs for each ligand. For the values of loop-closure while the ligand is within the active site, the average was proportionally weighted towards runs in which the ligand occupies the active site for a longer period.

**Table 3:** Averaged clustering results and standard deviations.

		Apo	1	2	3	4	5	6	7
**CS-like Population**		38%	91%	97%	75%	59%	67%	32%	27%
Std. Dev.		42%	17%	8%	33%	25%	22%	31%	29%
**Loop Closed Population** a	**Overall**	60%	100%	100%	78%	77%	79%	37%	32%
	Std. Dev.	31%	1%	0%	29%	32%	35%	33%	34%
	**While Ligand is in Active Site** b	–	100%	100%	78%	88%	76%	20%	35%
	Std. Dev.	–	1%	0%	32%	36%	46%	18%	27%
**Ligand in Active Site Population** a		–	100%	100%	97%	38%	50%	61%	57%
	Std. Dev.		0%	0%	4%	40%	50%	39%	45%
**Populated Enzyme Conformations**		3.0	1.4	1.2	1.8	2.8	2.6	2.9	2.8
	Std. Dev.	0.6	0.7	0.6	0.8	1.6	1.2	1.4	1.1
**Populated Ligand Conformations**		–	1.4	2.0	4.8	5.3	7.0	7.7	6.3
	Std. Dev.		0.4	0.5	1.8	2.8	3.7	4.5	4.1

a See materials and methods for criteria.

b Percentage of simulation time that a loop-closed cluster is occupied while the ligand remains in the active site.

## Supporting Information

Figure S1 **Comparison of cavity-width and ligand distance from active site for compound 2.**
(TIF)

Text S1 **Cluster population over time results.**
(PDF)

Text S2 **Description of clustering results for ligands 4 and 7.**
(PDF)

Text S3 **Populated conformations of enzymes.**
(PDF)

Text S4 **Key ligand conformations.**
(PDF)

Text S5 **Simulation stability over time from 20 ns blocks of cluster populations.**
(PDF)

Text S6 **Root mean squared fluctuations of key residues.**
(PDF)

Text S7 **Atomic charges and atom types.**
(PDF)

Text S8 **Root mean squared deviation of all heavy NA atoms for all simulations**
(PDF)

Text S9 **Root mean squared deviation of heavy active site atoms for all simulations.**
(PDF)

Structures S1 **The coordinates of the top 20 most populated ligand and top 40 most populated enzyme clusters in PDB format.**
(ZIP)
