# Supplementary figures and images for: The Influence of 150-Cavity Binders on the Dynamics of Influenza A Neuraminidases as Revealed by Molecular Dynamics Simulations and Combined Clustering
Source: PLoS One. 2013 Mar 27;8(3):e59873. doi: 10.1371/journal.pone.0059873 (PMC3609799; doi:10.1371/journal.pone.0059873)

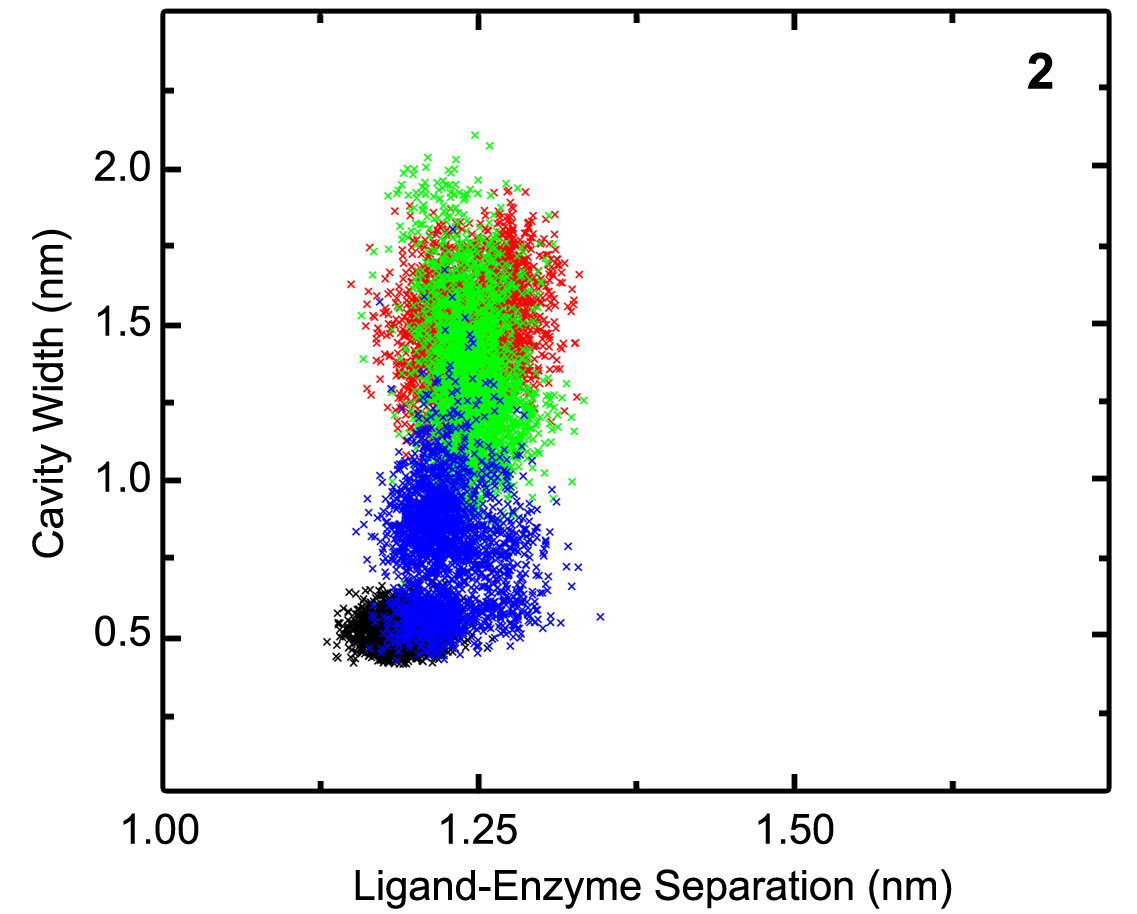

Supplement: Figure S1 — Comparison of cavity-width and ligand distance from active site for compound 2. (TIF) [file pone.0059873.s001.tif]

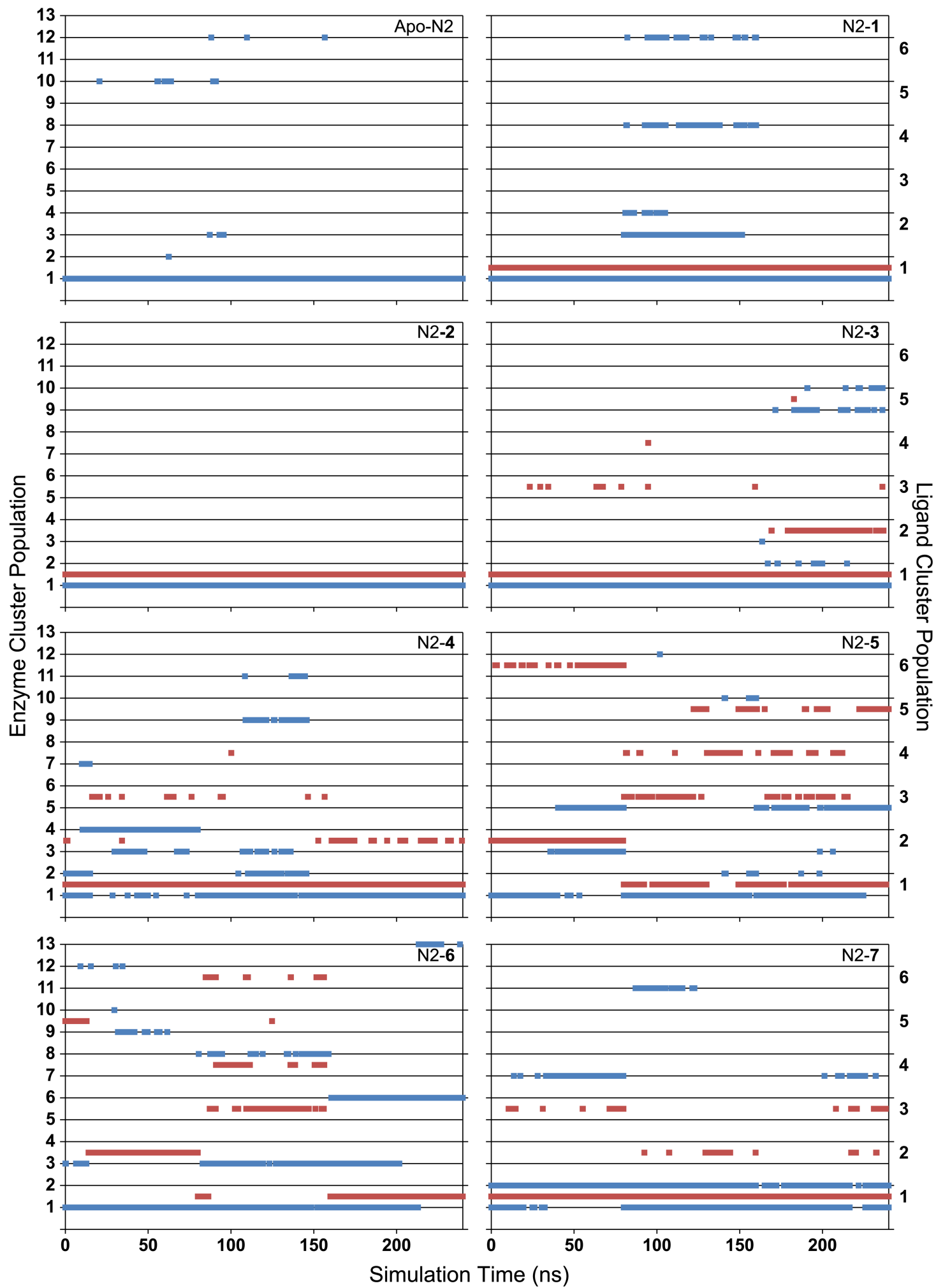

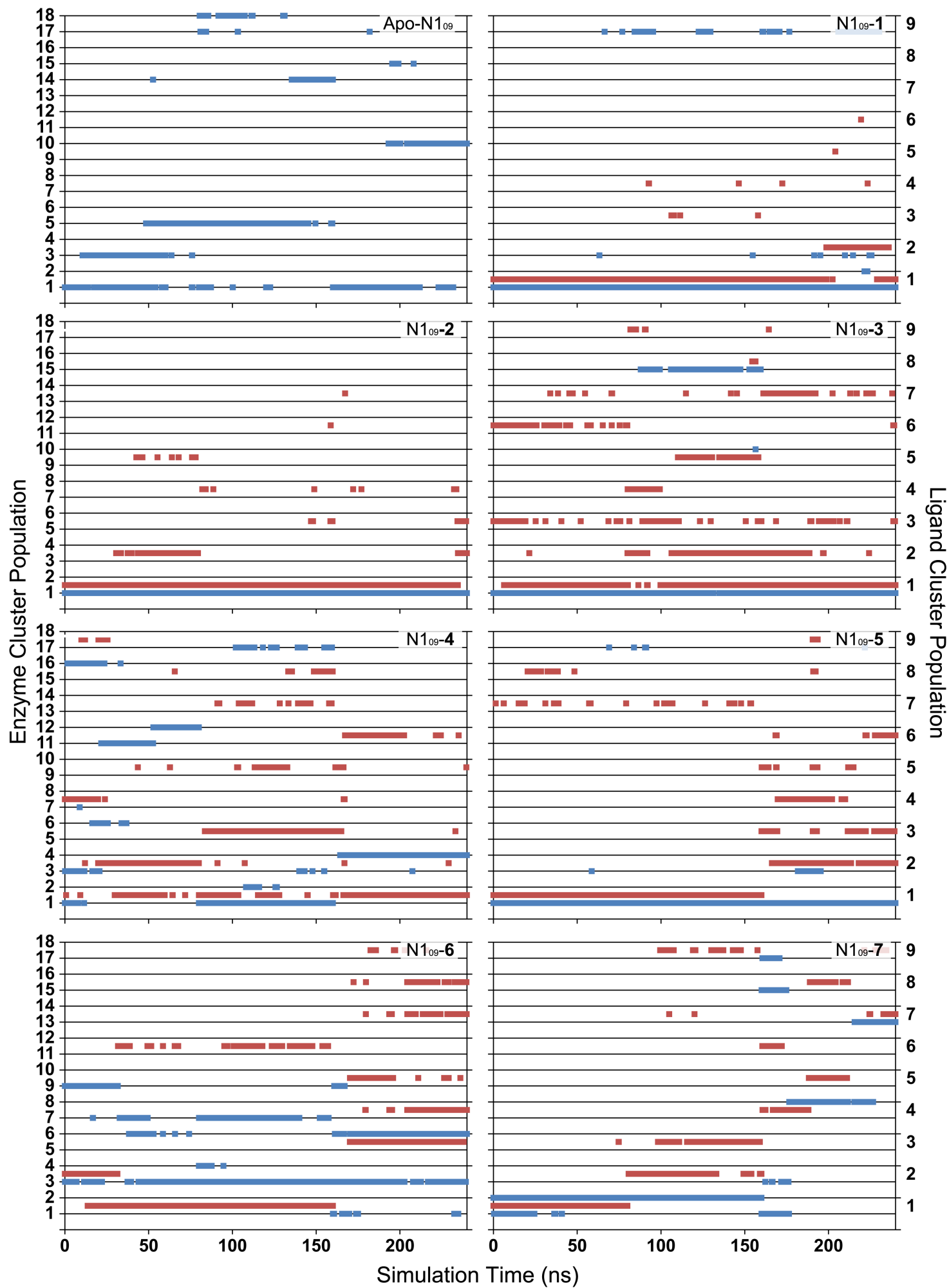

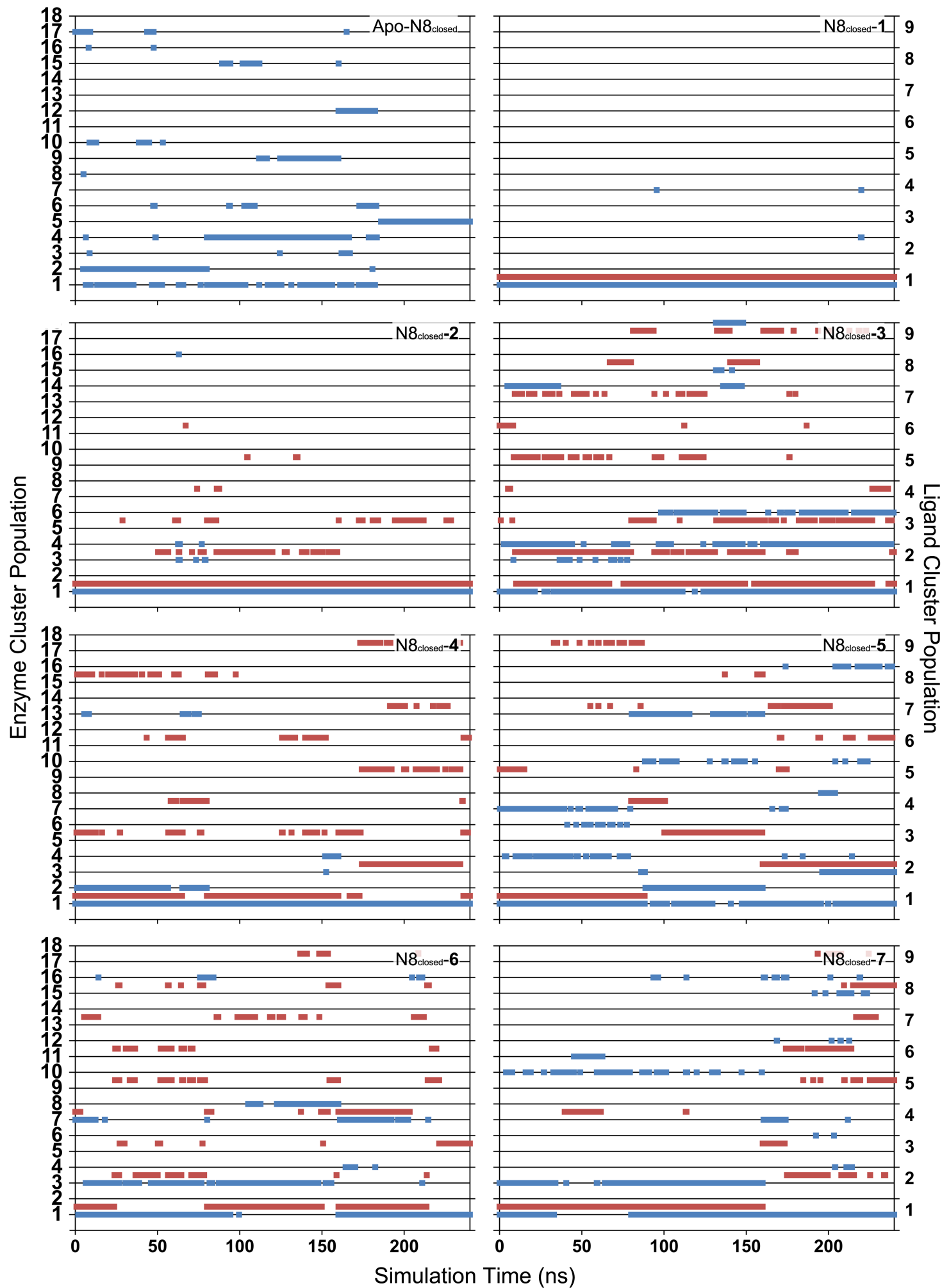

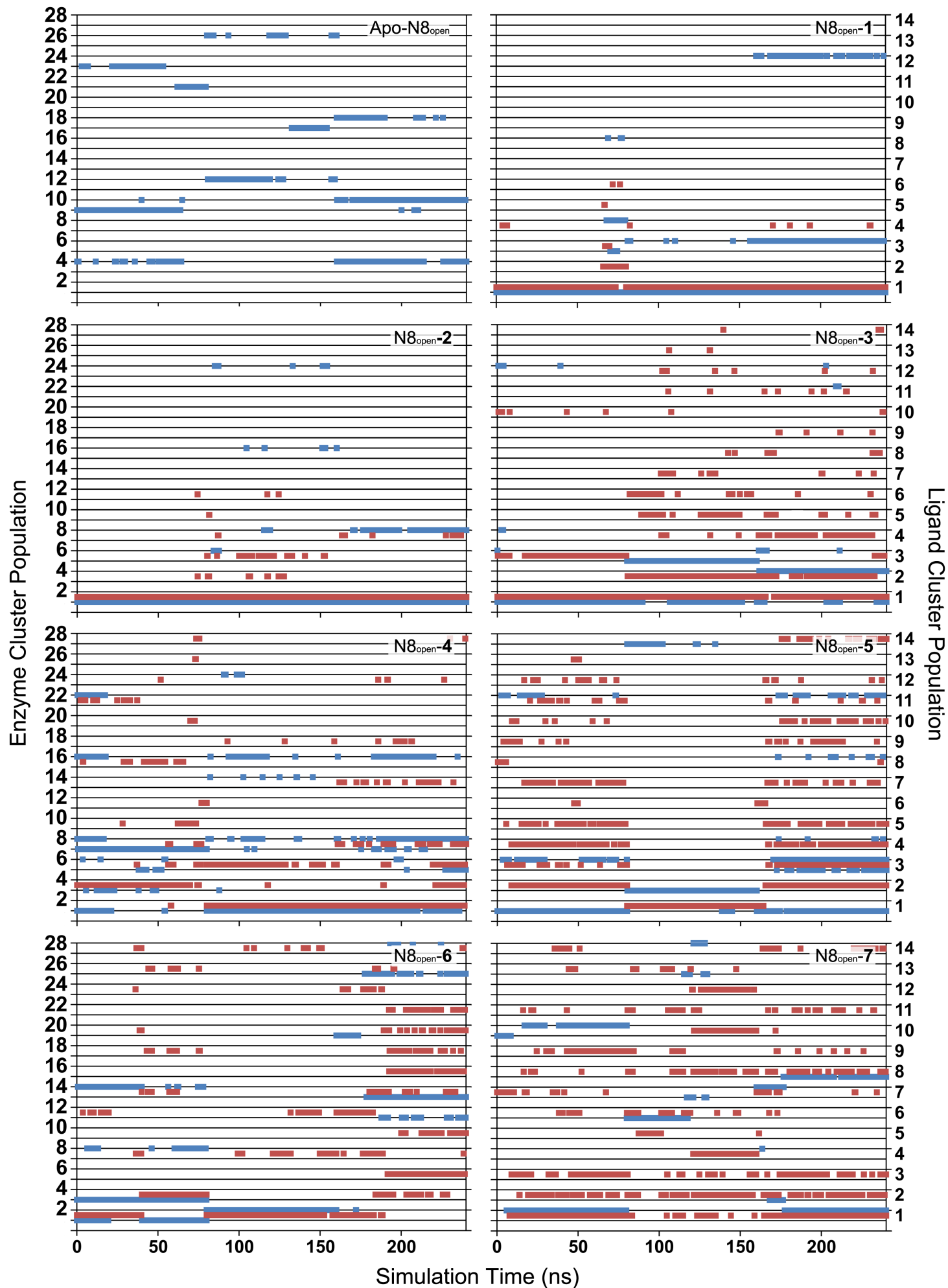

Supplement: Text S1 — Cluster population over time results. (PDF) [file pone.0059873.s002.pdf]

Supporting Information

Text S6. Root Mean Squared Fluctuations of Key Residues.

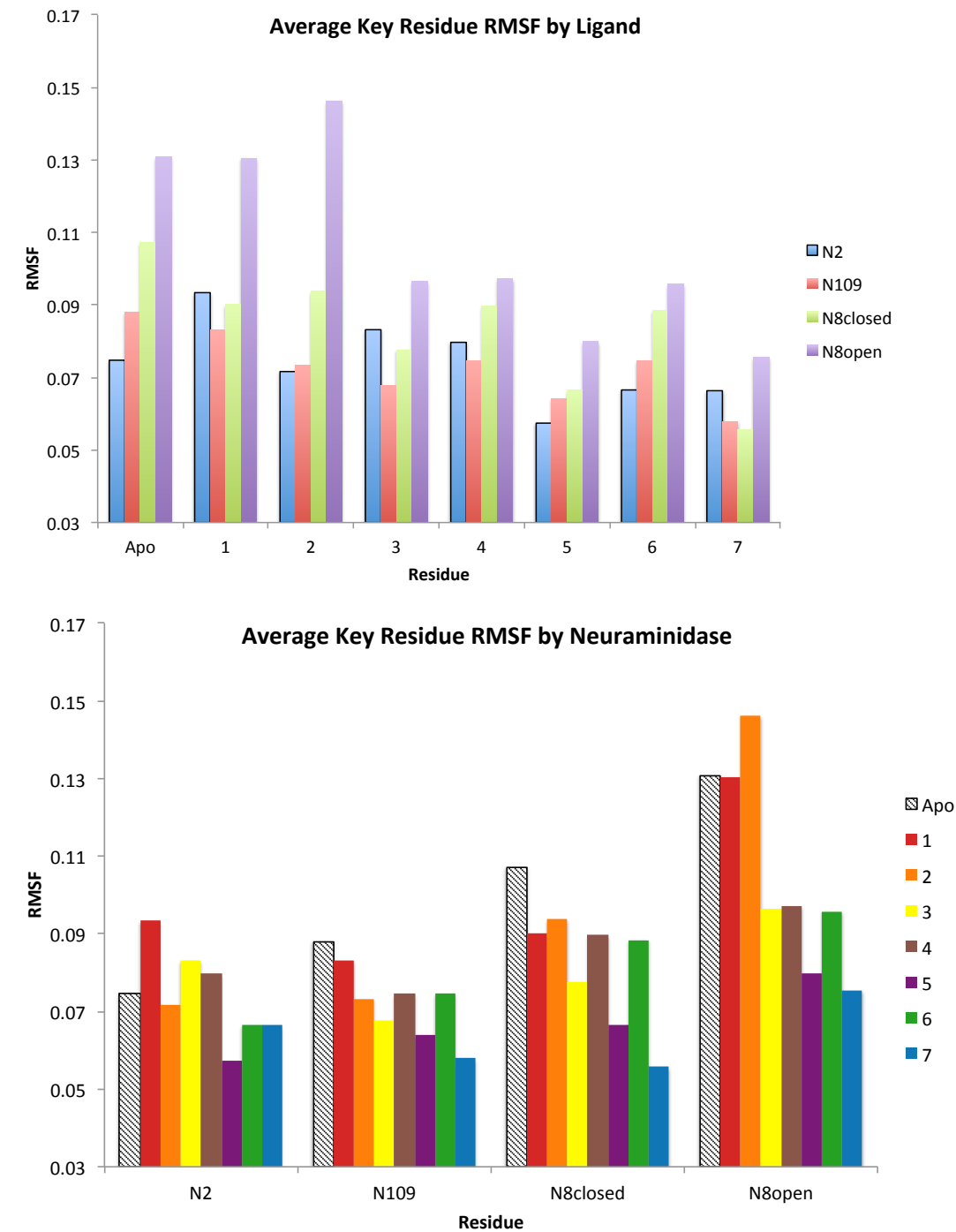

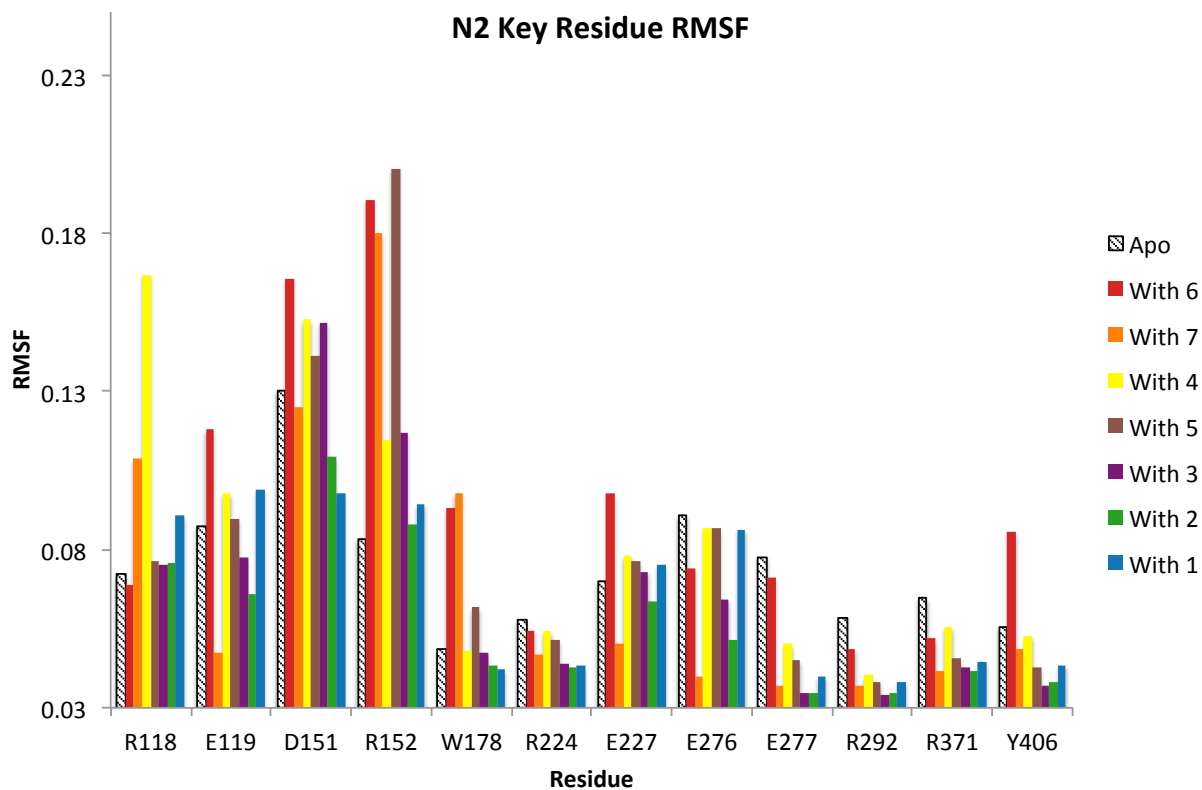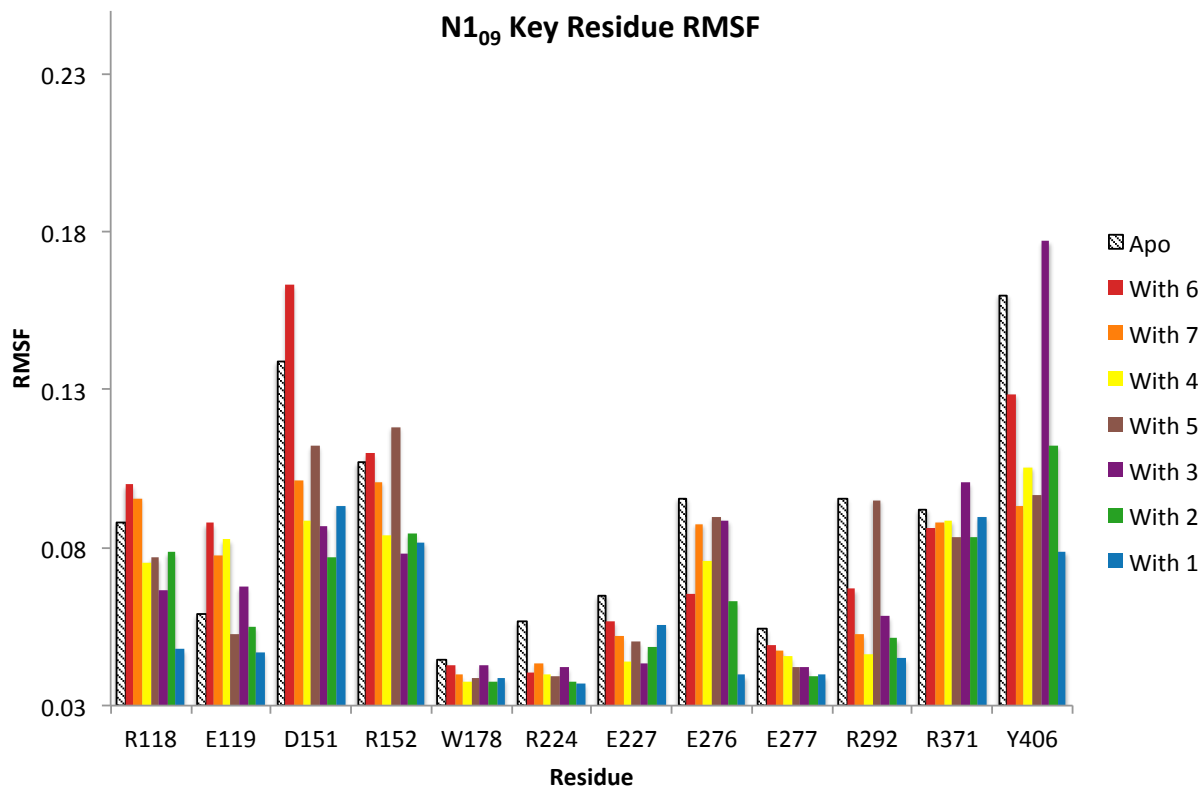

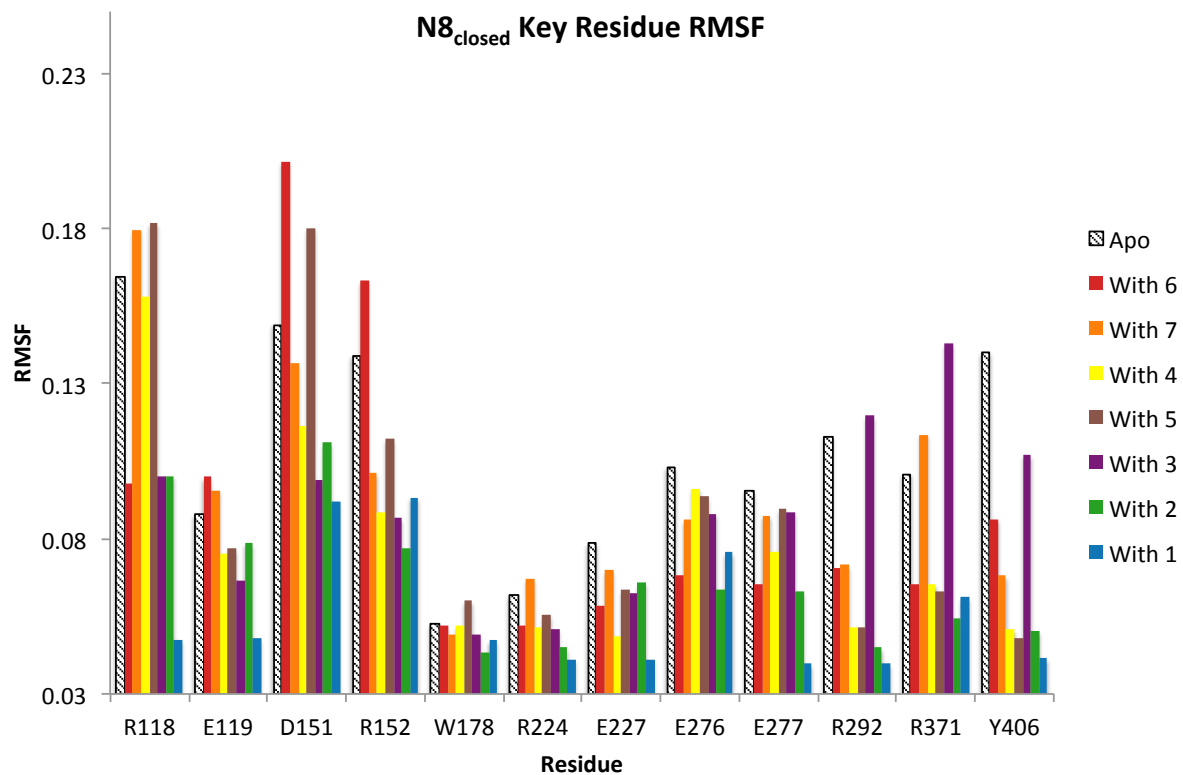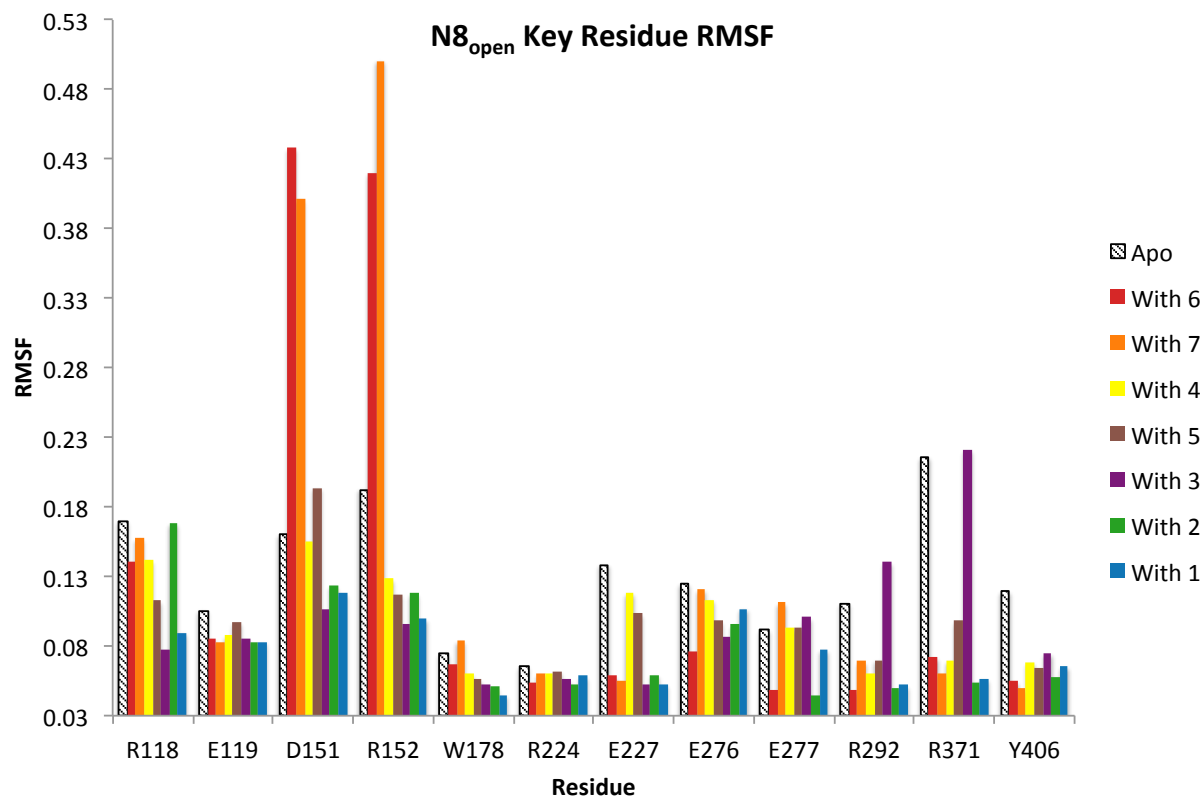

Supplement: Text S6 — Root mean squared fluctuations of key residues. (PDF) [file pone.0059873.s007.pdf]
