# Supplementary material for: The Influence of 150-Cavity Binders on the Dynamics of Influenza A Neuraminidases as Revealed by Molecular Dynamics Simulations and Combined Clustering
Source: PLoS One. 2013 Mar 27;8(3):e59873. doi: 10.1371/journal.pone.0059873 (PMC3609799; doi:10.1371/journal.pone.0059873)
Supplement: Text S2 — Description of clustering results for ligands 4 and 7. (PDF) [file pone.0059873.s003.pdf]

Supporting Information

Text S2. Description of clustering results for ligands 4 and 7

Conformations of NA Active Site and Complexed 150-Binder 4

With N2, **4** remains in its crystal-structure like pose almost constantly (L1; 98%) with its alkene-sidechain pointing out of the active site. The enzyme is stable in C1 (63%), and C4 (27%), where a cascade of shifts occur: Y406 swings toward E119 which bends toward the ligand's hydroxyl while R118 swings in to interact with D151 and the hydroxyl as well and D152 turns toward it. This suggests that the side group does not block open the loop, but nevertheless has an effect. Modest populations also exist where D151 and R152 are moved outwards somewhat (C2, C9; 6%) in comparison to enzyme-only runs.

With $N1_{09}$, the dynamics of the enzyme are completely altered as only the C1 pose (32%) is common between apo-$N1_{09}$ and $N1_{09}$-**4**. **4** is highly mobile, with nine clusters of 1.0% or greater population. It is often modified from a CS-like pose, typically being somewhat dislodged from the active site (L1, L2, L4, L6, L7; 74%) or occasionally completely outside of the active site (L9, L14, L16; 1%). Due to lacking good interactions at its fourth and fifth ring carbons, and fluctuations in R371 and R292, the side of the ring near the fifth carbon is quite mobile. The ligand is often shifted toward the withdrawn triarginyl cluster with the sidechain pointing into solution (L1, L4, L6; 49%), the fourth and fifth carbons are often lifted out somewhat (L2, L7; 30%), or pivoted toward R118 (L3; 8.2%). Occasionally the sidechain rotates into the active site (L2; 22%), presumably to avoid unfavorable interactions with solvent molecules, and this is often concomitant with the loop being open and D151 and R152 being significantly withdrawn (C11, C12; 87%). R292 and R371 are typically withdrawn somewhat (C3, C4, C11, C12, C16; 59.5%) while R152 is frequently extended toward the ligand's amide (C4, C16; 37%).

In complex with $N8_{closed}$, **4** mostly resembles the crystal structure pose (L1, L3, L8; 64%) though the side of the fourth and fifth carbons frequently lifts out of the active site (L2, L5, L7; 15%) and the ligand exhibits translational movement within the active site (L4, L6; 7%). The sidechain nearly always points into solution, with a brief time spent pointing into the active site (L7; 2%) and only when the ring is lifted up. Carboxylate-arginine interactions are extremely stable however, and the enzyme is quite static. It is typically in its crystal structure pose (C1; 90%) while R118 and D151 occasionally move back together (C2; 7%), likely due to a lack of interactions with the hydroxyl group. R371 and R292 are static.

In the $N8_{open}$-**4** simulations, the loop is never fully open. Instead, during one of the triplicates, the ligand is unusually rotated in the active site (L2; 30%) while D151 is slightly withdrawn and R118 is bent toward it, being recessed only slightly (C7, C8; 33%). In the remaining two triplicates, the ligand's sidechain is CS-like (L1; 83%) or pointing into solution (L3, L4; 16%) while the enzyme is CS-like (C1; 73%). This indicates the strong stabilizing effects of **4**, comparable to the standard binders, and that the sidechain typically does not affect the 150-loop while inside or outside of the active site.

Conformations of NA Active Site and Complexed 150-Binder 7

With N2, **7** is extremely stable and almost entirely in a crystal structure-like pose (L1, 97%). The NA meanwhile alternates between C1 (42%) and C2/C7 (56%), the latter exhibiting D151 and R152 simultaneously withdrawn due to clash between the ligand's triazole and D151. In these conformations, R118 is further moved in somewhat to interact with the ligand's sidechain, which periodically bends to bring the terminal hydroxyl closer (L2; 2%). These results show that **7** can extend into the 150-cavity without disturbing the D151 and R152 interactions significantly unlike **1**.

With $N1_{09}$, **7** is often in its CS-like pose in the active site (L1; 32%), which often has D151 and R152 moved back toward W178 and mutually interacting (C2; 62%) – much like C2

seen in the N2 simulations. The sidechain frequently exits the active site, giving rise to ligand clusters that are progressively further from the active site (L2-L10; 46%), as does the isopentyl group to a lesser degree. When D151 returns to its crystal structure-like pose (C8; 13%), the ligand is unable to re-enter the active site. The ligand is extremely flexible, especially outside of the active site, which gives rise to numerous poses; 16 above 1.0 % population. The carboxylate interaction is extremely constant. C1 and L1 are almost entirely incompatible, with only a 11% incidence of C1 when L1 occurs.

In complex with $N8_{closed}$, **7** is typically rotated from an CS-like pose (L1; 61%), while the enzyme exhibits its crystal structure-like conformation (C1; 59%). D151 is frequently swung toward to interact with the side-chain hydroxyl and R118 interacting strongly with the ligand's triazole and pushed back (C3, C10; 30.0%). The ligand carboxylate often swings out, exiting the active site (L4; 4%), and the R371 contact is typically concomitantly lost (C11; 81%) allowing R118 to swing in and interact with the extended D151 in turn. When the central ring is in a CS-like pose, the sidechain is extremely mobile and more often exited (L2, L5, L6; 15%) than not (L3; 5%) while the enzyme adopts C1 (92%).

For $N8_{open}$ in complex with **7**, the loop never closes and there is zero population of C1. During two of the triplicates, C2 is the most populated (62%) featuring a widely open loop as seen in $N8_{closed}$-**6** while the ligand's sidechain is fluctuating significantly outside of the active site (L1, L2, L3, L6, L7, L8, L9, L11; 88%). During the remaining triplicate, the ligand occasionally adopts a CS-like pose (L5; 15%) but otherwise demonstrates the same behavior as the remaining two triplicates. The enzyme, meanwhile, demonstrates more widely loop-open poses. This demonstrates that, in absence of the dynamic loop's interactions, the ligand is generally unstable within the active site. In fact, the ligand lifts out of the active entirely, except for the carboxylate region, for the bulk of the total simulation (L1-L4, L6, L8-L12; 80%).
