# Supplementary material for: The Influence of 150-Cavity Binders on the Dynamics of Influenza A Neuraminidases as Revealed by Molecular Dynamics Simulations and Combined Clustering
Source: PLoS One. 2013 Mar 27;8(3):e59873. doi: 10.1371/journal.pone.0059873 (PMC3609799; doi:10.1371/journal.pone.0059873)
Supplement: Text S3 — Populated conformations of enzymes. (PDF) [file pone.0059873.s004.pdf]

Supporting Information

Text S3. Populated Conformations of Enzymes

N2 Populations of Top 18 Conformations

	Ligand							
**Conformation**	**None**	**1**	**2**	**3**	**4**	**5**	**6**	**7**
**1**	99%	100%	98%	63%	67%	58%	42%	86%
**2**	0%	0%	1%	3%	0%	0%	47%	0%
**3**	0%	0%	0%	2%	6%	15%	0%	10%
**4**	0%	0%	0%	27%	0%	0%	0%	1%
**5**	0%	0%	0%	0%	25%	0%	0%	0%
**6**	0%	0%	0%	0%	0%	22%	0%	0%
**7**	0%	0%	0%	0%	0%	0%	9%	0%
**8**	0%	0%	0%	0%	0%	3%	0%	3%
**9**	0%	0%	1%	3%	0%	1%	0%	0%
**10**	1%	0%	0%	0%	1%	0%	0%	0%
**11**	0%	0%	0%	0%	0%	0%	1%	0%
**12**	0%	0%	0%	0%	0%	0%	0%	1%
**13**	0%	0%	0%	0%	0%	1%	0%	0%
**14**	0%	0%	0%	0%	0%	0%	0%	0%
**15**	0%	0%	0%	1%	0%	0%	0%	0%
**16**	0%	0%	0%	0%	0%	0%	0%	0%
**17**	0%	0%	0%	0%	0%	0%	0%	0%
**18**	0%	0%	0%	0%	0%	0%	0%	0%
**Sum**	100%	100%	100%	100%	100%	100%	100%	100%

N2 Cluster Structures

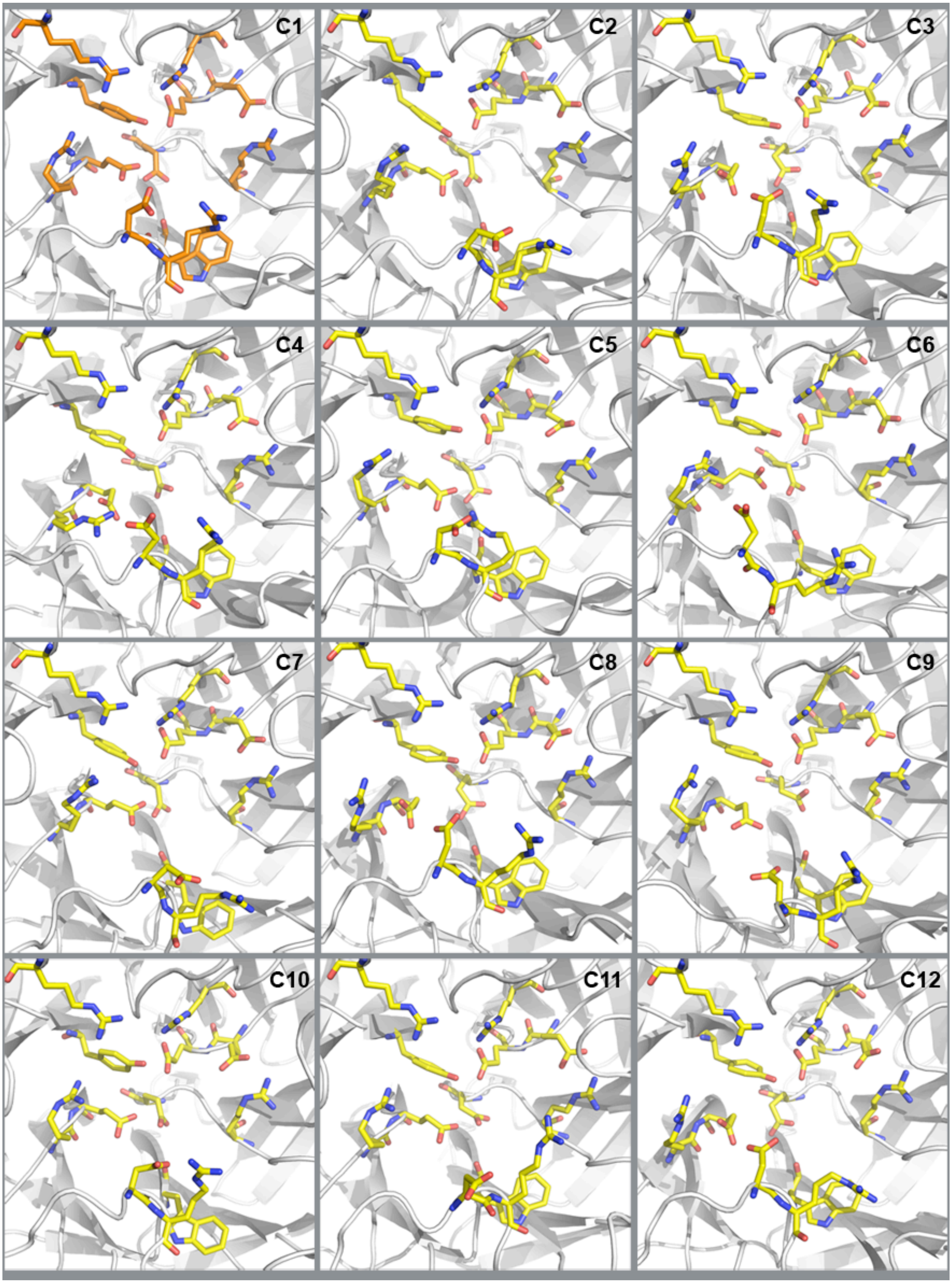

N1$_{09}$ Populations of Top 18 Conformations

	Ligand							
**Conformation**	**None**	**1**	**2**	**3**	**4**	**5**	**6**	**7**
**1**	34%	96%	100%	93%	32%	98%	1%	5%
**2**	0%	0%	0%	0%	0%	0%	0%	62%
**3**	7%	0%	0%	0%	2%	2%	40%	0%
**4**	0%	0%	0%	0%	31%	0%	0%	0%
**5**	31%	0%	0%	0%	0%	0%	0%	0%
**6**	0%	0%	0%	0%	1%	0%	23%	0%
**7**	0%	0%	0%	0%	0%	0%	17%	0%
**8**	0%	0%	0%	0%	0%	0%	0%	13%
**9**	0%	0%	0%	0%	0%	0%	13%	0%
**10**	12%	0%	0%	0%	0%	0%	0%	0%
**11**	0%	0%	0%	0%	12%	0%	0%	0%
**12**	0%	0%	0%	0%	9%	0%	0%	0%
**13**	0%	0%	0%	0%	0%	0%	0%	9%
**14**	8%	0%	0%	0%	0%	0%	0%	0%
**15**	0%	0%	0%	5%	0%	0%	0%	3%
**16**	0%	0%	0%	0%	6%	0%	0%	0%
**17**	0%	3%	0%	0%	2%	0%	0%	0%
**18**	4%	0%	0%	0%	0%	0%	0%	0%
**Sum**	97%	99%	100%	98%	95%	100%	93%	93%

$N1_{09}$ Cluster Structures

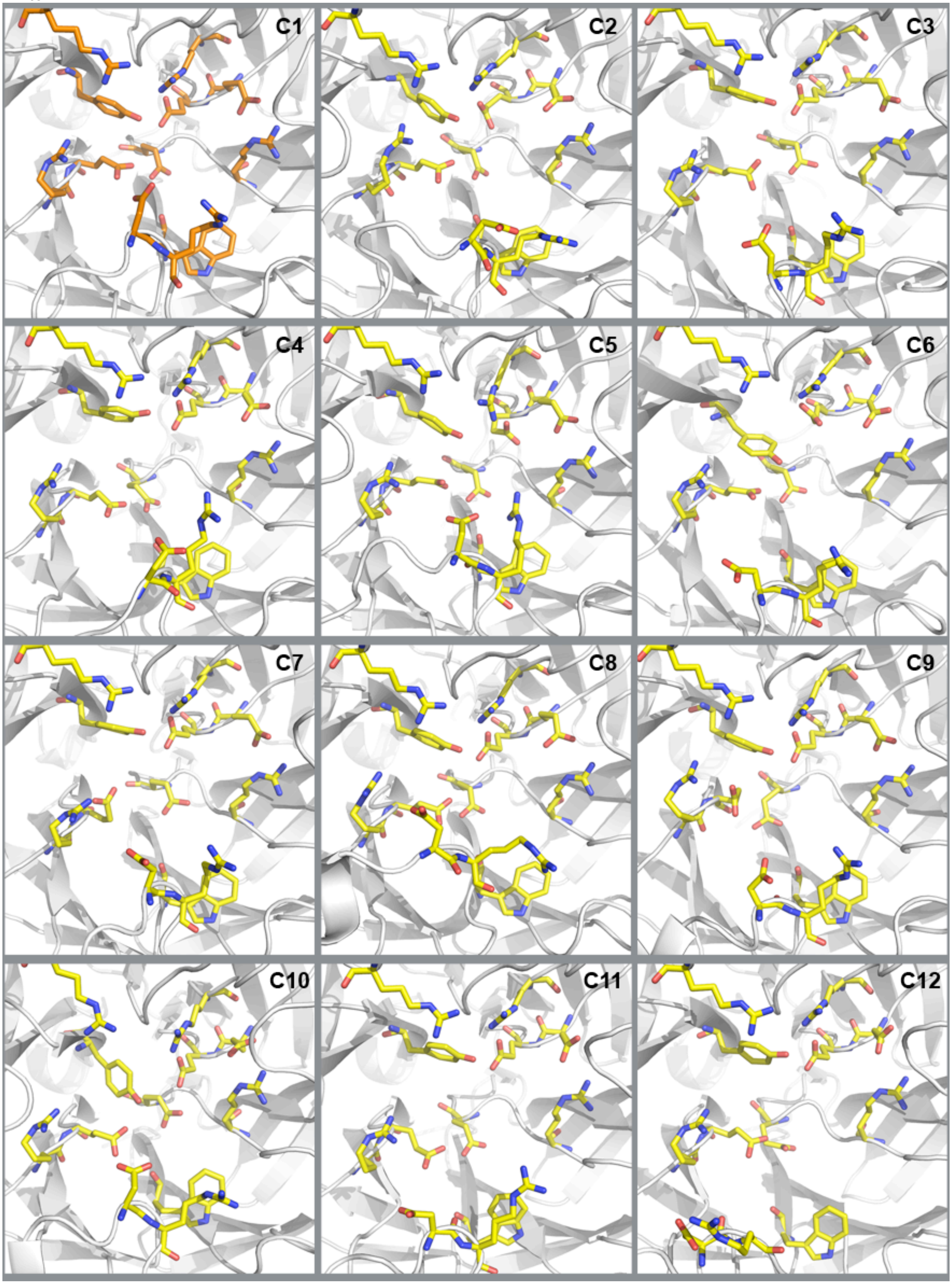

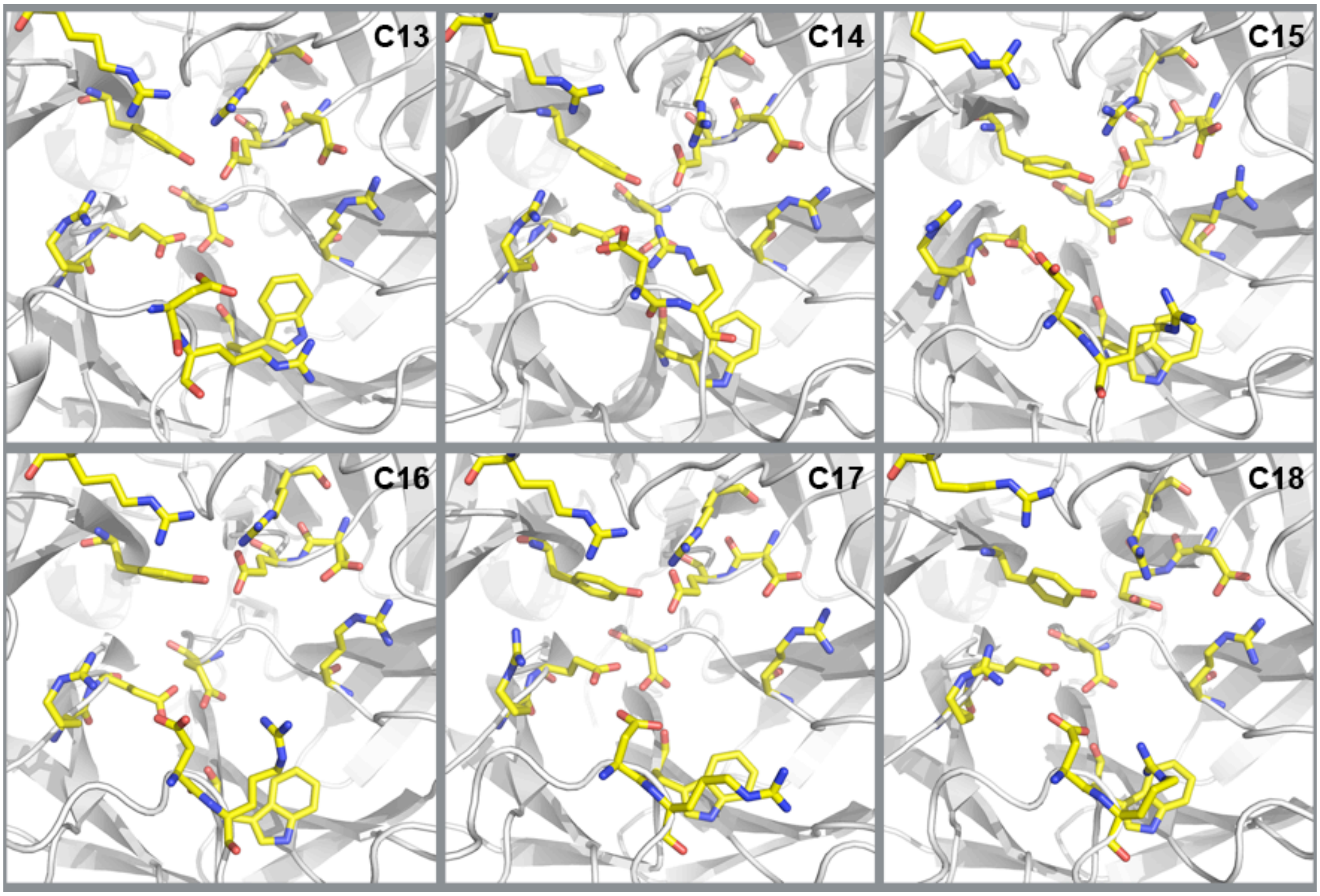
C13
C14
C15
C16
C17
C18

$N8_{closed}$ Populations of Top 18 Conformations

	Ligand							
**Conformation**	**None**	**1**	**2**	**3**	**4**	**5**	**6**	**7**
**1**	15%	100%	100%	66%	89%	55%	58%	59%
**2**	24%	0%	0%	0%	7%	22%	0%	0%
**3**	0%	0%	0%	1%	0%	5%	24%	22%
**4**	10%	0%	0%	15%	1%	2%	0%	0%
**5**	19%	0%	0%	0%	0%	0%	0%	0%
**6**	2%	0%	0%	12%	0%	0%	0%	0%
**7**	0%	0%	0%	0%	0%	4%	8%	1%
**8**	0%	0%	0%	0%	0%	3%	8%	0%
**9**	10%	0%	0%	0%	0%	0%	0%	0%
**10**	0%	0%	0%	0%	0%	2%	0%	8%
**11**	0%	0%	0%	0%	0%	0%	0%	6%
**12**	5%	0%	0%	0%	0%	0%	0%	0%
**13**	0%	0%	0%	0%	1%	3%	0%	0%
**14**	0%	0%	0%	4%	0%	0%	0%	0%
**15**	3%	0%	0%	0%	0%	0%	0%	0%
**16**	0%	0%	0%	0%	0%	1%	1%	1%
**17**	3%	0%	0%	0%	0%	0%	0%	0%
**18**	0%	0%	0%	2%	0%	0%	0%	0%
**Sum**	91%	100%	100%	99%	99%	98%	99%	97%

$N8_{closed}$ Cluster Structures

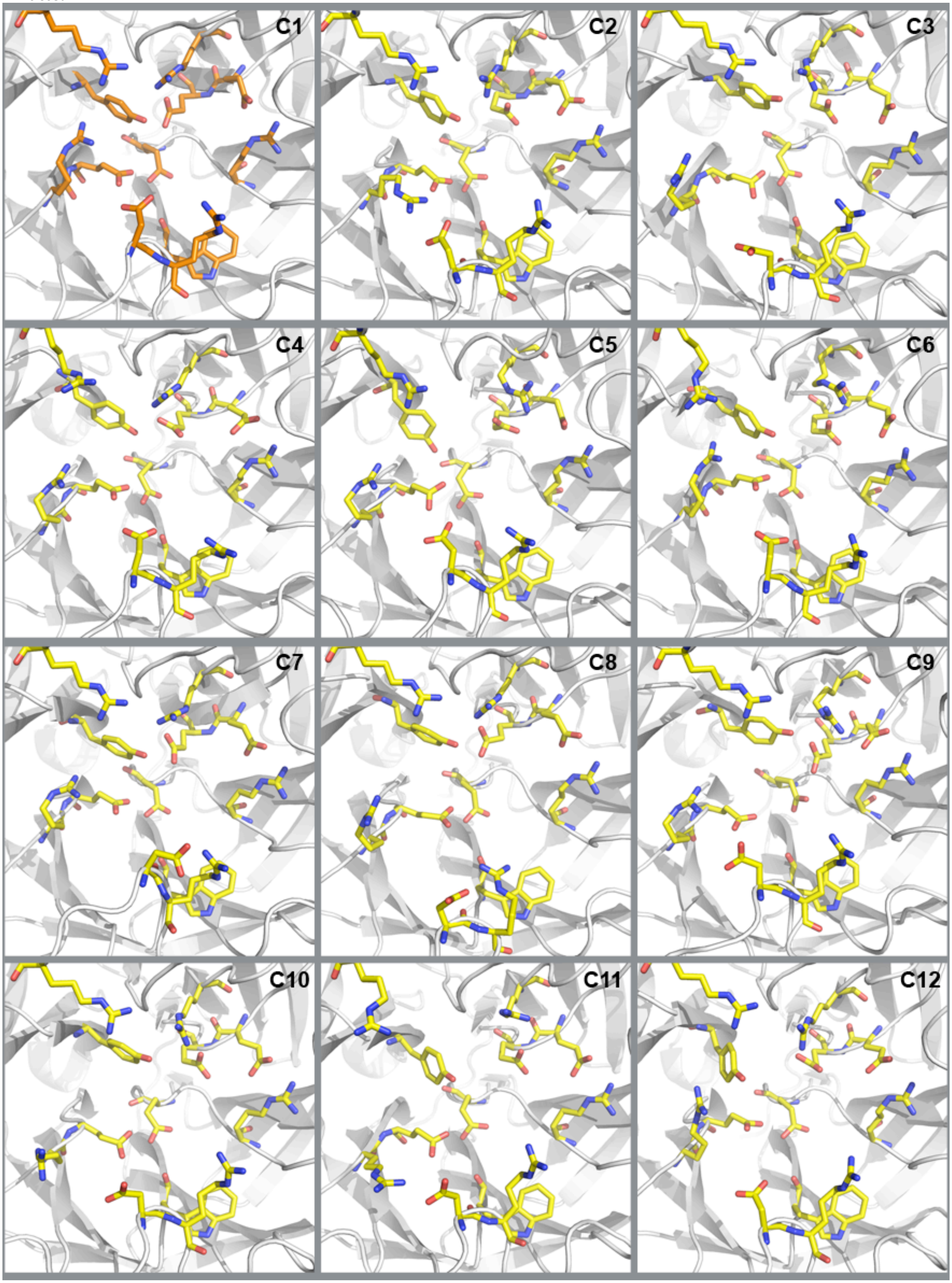

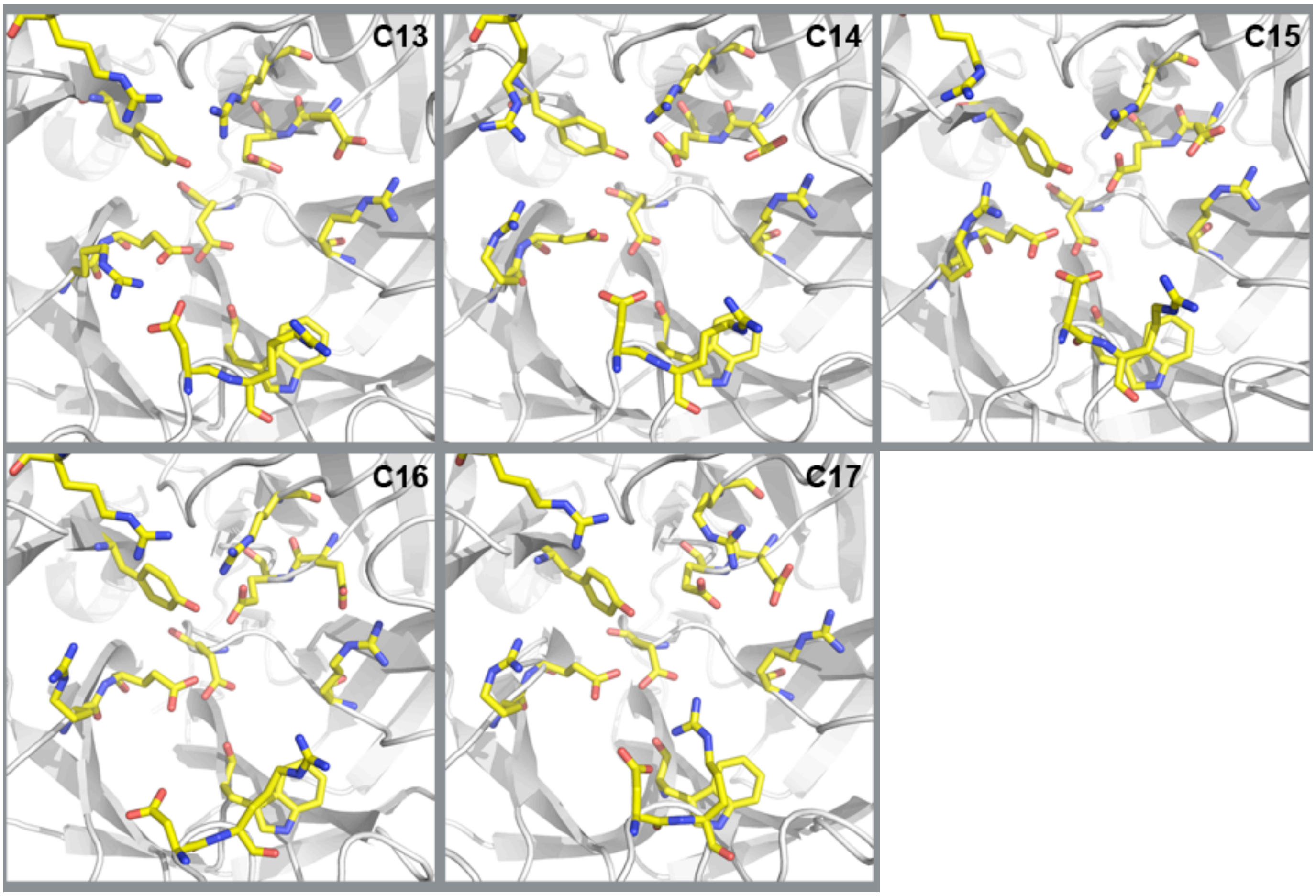
C13
C14
C15
C16
C17

N8$_{open}$ Populations of Top 28 Conformations

	Ligand							
**Conformation**	**None**	**1**	**2**	**3**	**4**	**5**	**6**	**7**
**1**	0%	82%	82%	41%	49%	45%	8%	0%
**2**	0%	0%	0%	0%	0%	0%	33%	40%
**3**	0%	0%	0%	0%	1%	30%	19%	0%
**4**	11%	0%	0%	28%	0%	0%	0%	0%
**5**	0%	0%	0%	28%	2%	2%	0%	0%
**6**	0%	14%	14%	1%	0%	15%	0%	0%
**7**	0%	0%	0%	0%	25%	0%	0%	0%
**8**	0%	0%	0%	0%	8%	0%	2%	0%
**9**	16%	0%	0%	0%	0%	0%	0%	0%
**10**	15%	0%	0%	0%	0%	0%	0%	0%
**11**	0%	0%	0%	0%	0%	0%	1%	13%
**12**	12%	0%	0%	0%	0%	0%	0%	0%
**13**	0%	0%	0%	0%	0%	0%	11%	1%
**14**	0%	0%	0%	0%	0%	0%	4%	6%
**15**	0%	0%	0%	0%	0%	0%	0%	9%
**16**	0%	0%	0%	0%	8%	0%	0%	0%
**17**	7%	0%	0%	0%	0%	0%	0%	0%
**18**	7%	0%	0%	0%	0%	0%	0%	0%
**19**	0%	0%	0%	0%	0%	0%	4%	3%
**20**	0%	0%	0%	0%	0%	0%	0%	6%
**21**	6%	0%	0%	0%	0%	0%	0%	0%
**22**	0%	0%	0%	0%	2%	3%	0%	0%
**23**	4%	0%	0%	0%	0%	0%	0%	0%
**24**	0%	3%	3%	0%	0%	0%	0%	0%
**25**	0%	0%	0%	0%	0%	0%	4%	0%
**26**	4%	0%	0%	0%	0%	0%	0%	0%
**27**	0%	0%	0%	0%	0%	3%	0%	0%
**28**	0%	0%	0%	0%	0%	0%	1%	2%
**Sum**	81%	100%	100%	98%	95%	99%	86%	80%

$N8_{open}$ Cluster Structures

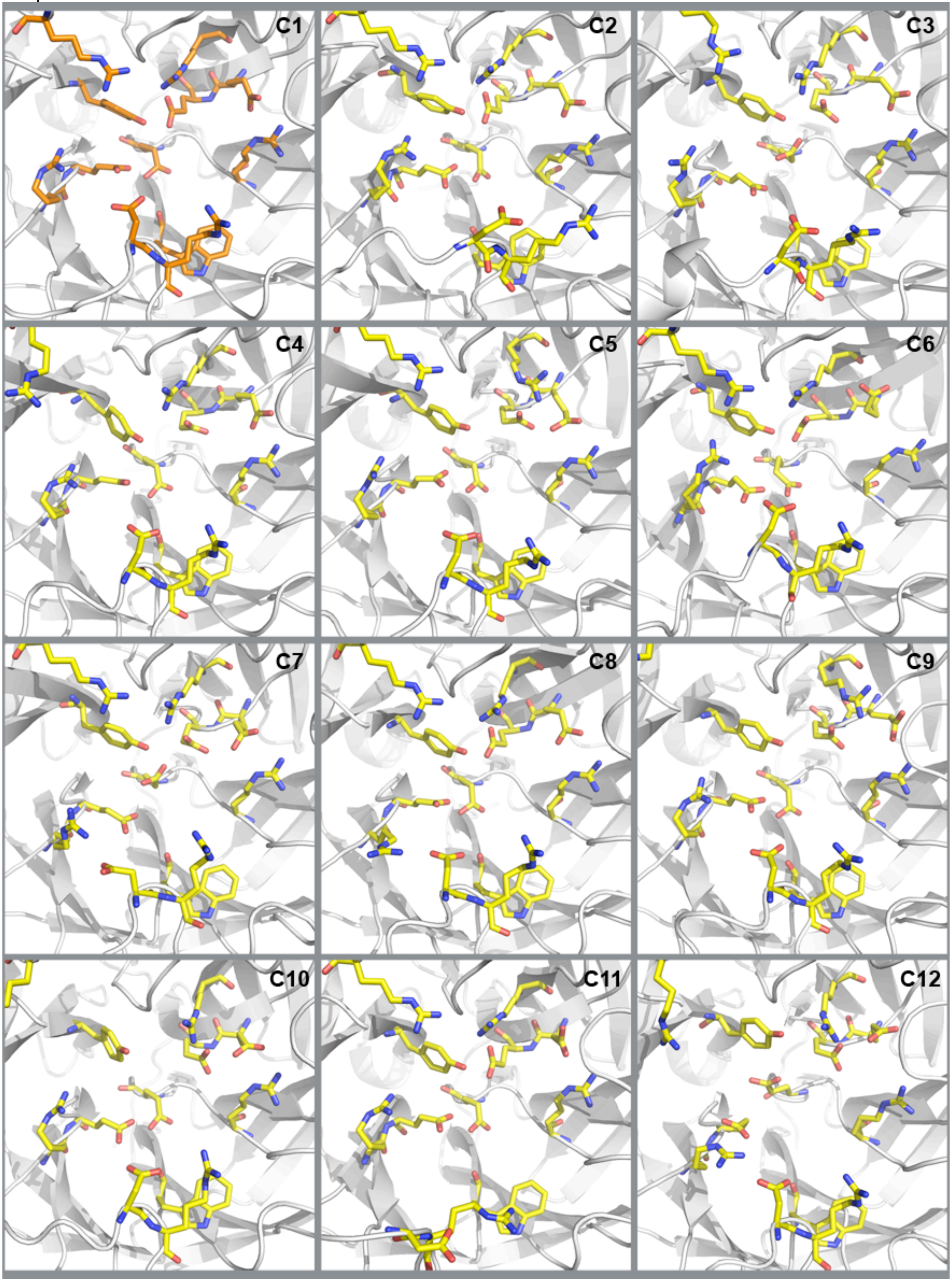

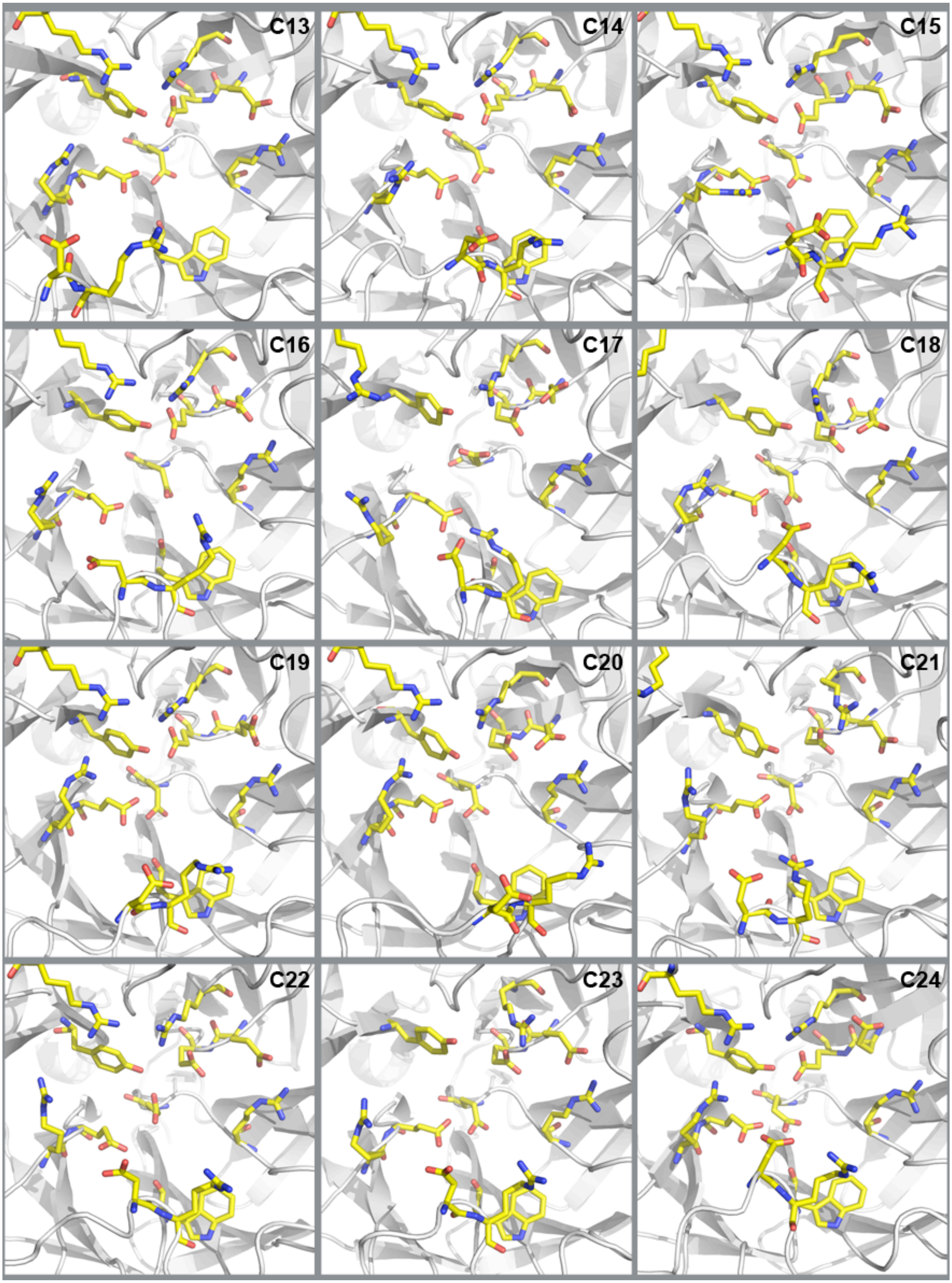
C13
C14
C15
C16
C17
C18
C19
C20
C21
C22
C23
C24

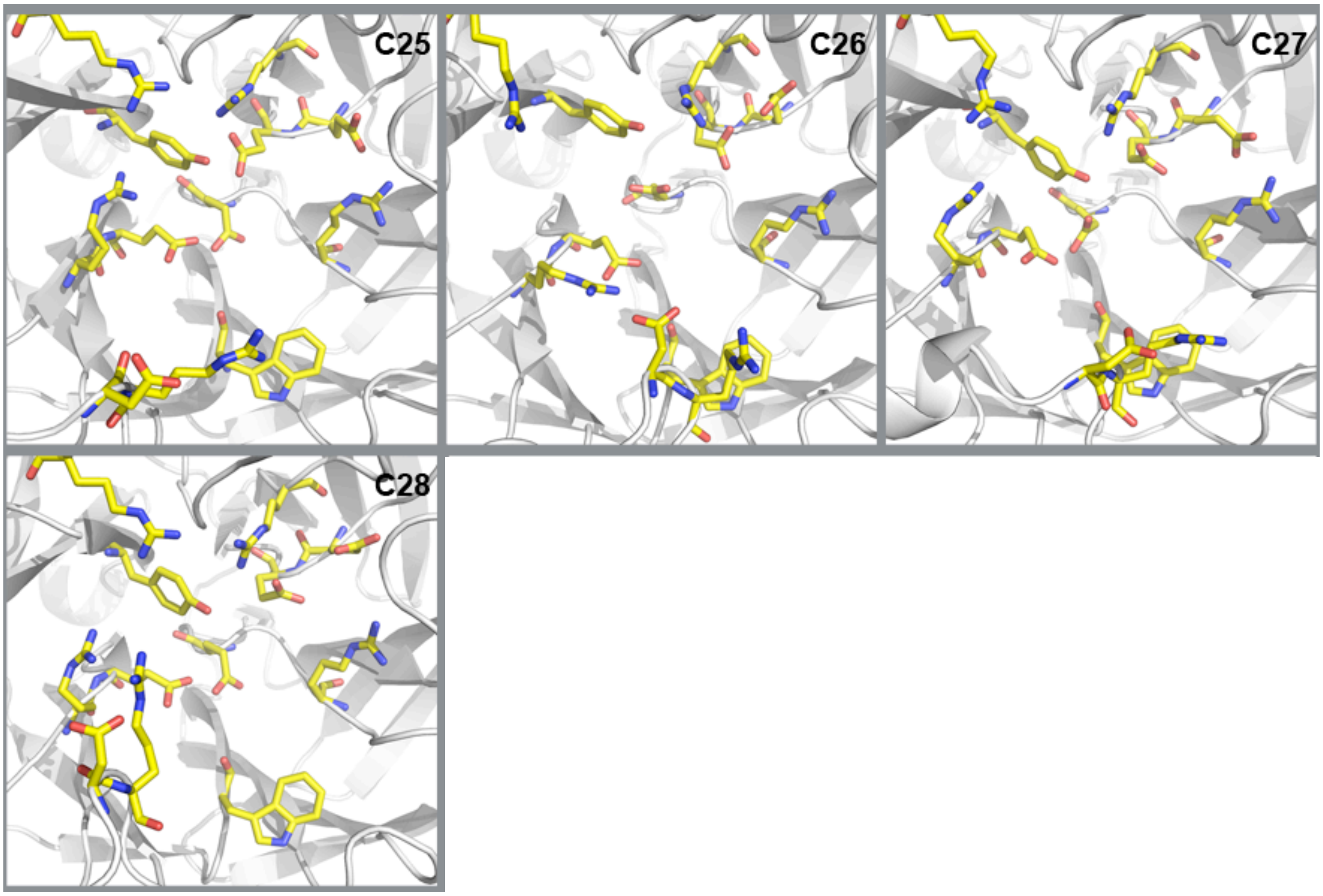
C25
C26
C27
C28
