# Supplementary material for: The Influence of 150-Cavity Binders on the Dynamics of Influenza A Neuraminidases as Revealed by Molecular Dynamics Simulations and Combined Clustering
Source: PLoS One. 2013 Mar 27;8(3):e59873. doi: 10.1371/journal.pone.0059873 (PMC3609799; doi:10.1371/journal.pone.0059873)

Supporting Information

Text S4. Key Ligand Conformations.

Ligand 1 Populations of Top 14 Conformations

	Neuraminidase			
**Conformation**	**N2**	**$N1_{09}$**	**$N8_{closed}$**	**$N8_{open}$**
**1**	100%	84%	100%	95%
**2**	0%	15%	0%	4%
**3**	0%	0%	0%	0%
**4**	0%	0%	0%	0%
**5**	0%	0%	0%	0%
**6**	0%	0%	0%	0%
**7**	0%	0%	0%	0%
**8**	0%	0%	0%	0%
**9**	0%	0%	0%	0%
**10**	0%	0%	0%	0%
**11**	0%	0%	0%	0%
**12**	0%	0%	0%	0%
**13**	0%	0%	0%	0%
**14**	0%	0%	0%	0%
**Sum**	100%	100%	100%	100%

Ligand 1 Key Cluster Structures

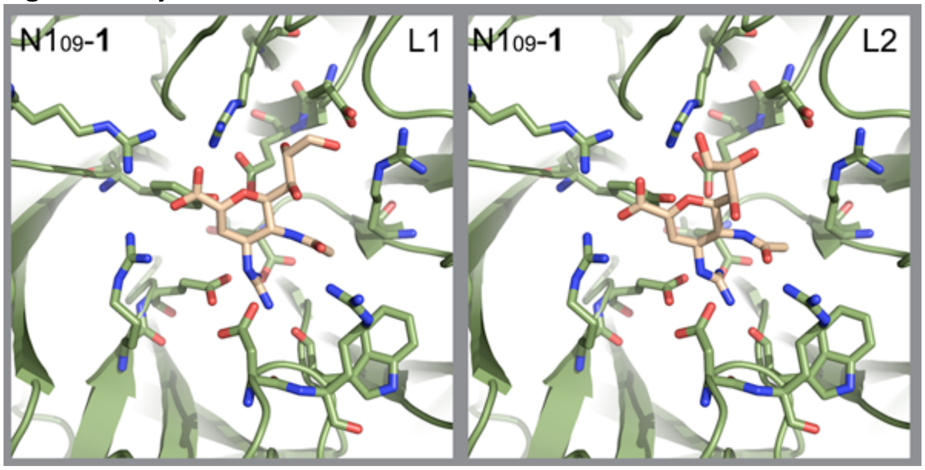

Ligand 2 Populations of Top 14 Conformations

Conformation	Neuraminidase			
	N2	$N1_{09}$	$N8_{closed}$	$N8_{open}$
**1**	100%	94%	96%	97%
**2**	0%	4%	2%	1%
**3**	0%	1%	1%	1%
**4**	0%	0%	0%	0%
**5**	0%	0%	0%	0%
**6**	0%	0%	0%	0%
**7**	0%	0%	0%	0%
**8**	0%	0%	0%	0%
**9**	0%	0%	0%	0%
**10**	0%	0%	0%	0%
**11**	0%	0%	0%	0%
**12**	0%	0%	0%	0%
**13**	0%	0%	0%	0%
**14**	0%	0%	0%	0%
**Sum**	100%	100%	100%	100%

Ligand 2 Key Cluster Structures

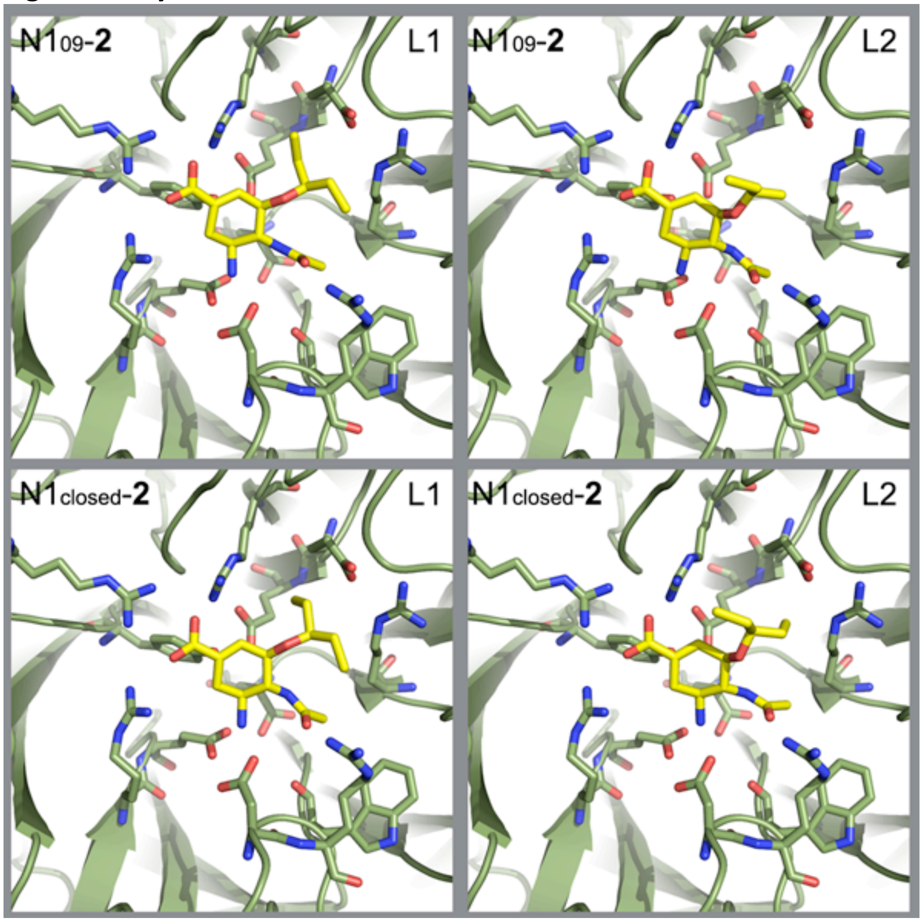

Ligand 3 Populations of Top 14 Conformations

Conformation	Neuraminidase			
	N2	$N1_{09}$	$N8_{closed}$	$N8_{open}$
**1**	97%	70%	55%	67%
**2**	3%	9%	20%	17%
**3**	0%	8%	9%	6%
**4**	0%	4%	4%	4%
**5**	0%	2%	4%	2%
**6**	0%	2%	3%	1%
**7**	0%	2%	1%	1%
**8**	0%	1%	1%	0%
**9**	0%	0%	1%	0%
**10**	0%	0%	0%	0%
**11**	0%	0%	0%	0%
**12**	0%	0%	0%	0%
**13**	0%	0%	0%	0%
**14**	0%	0%	0%	0%
**Sum**	100%	100%	100%	100%

Ligand 3 Key Cluster Structures

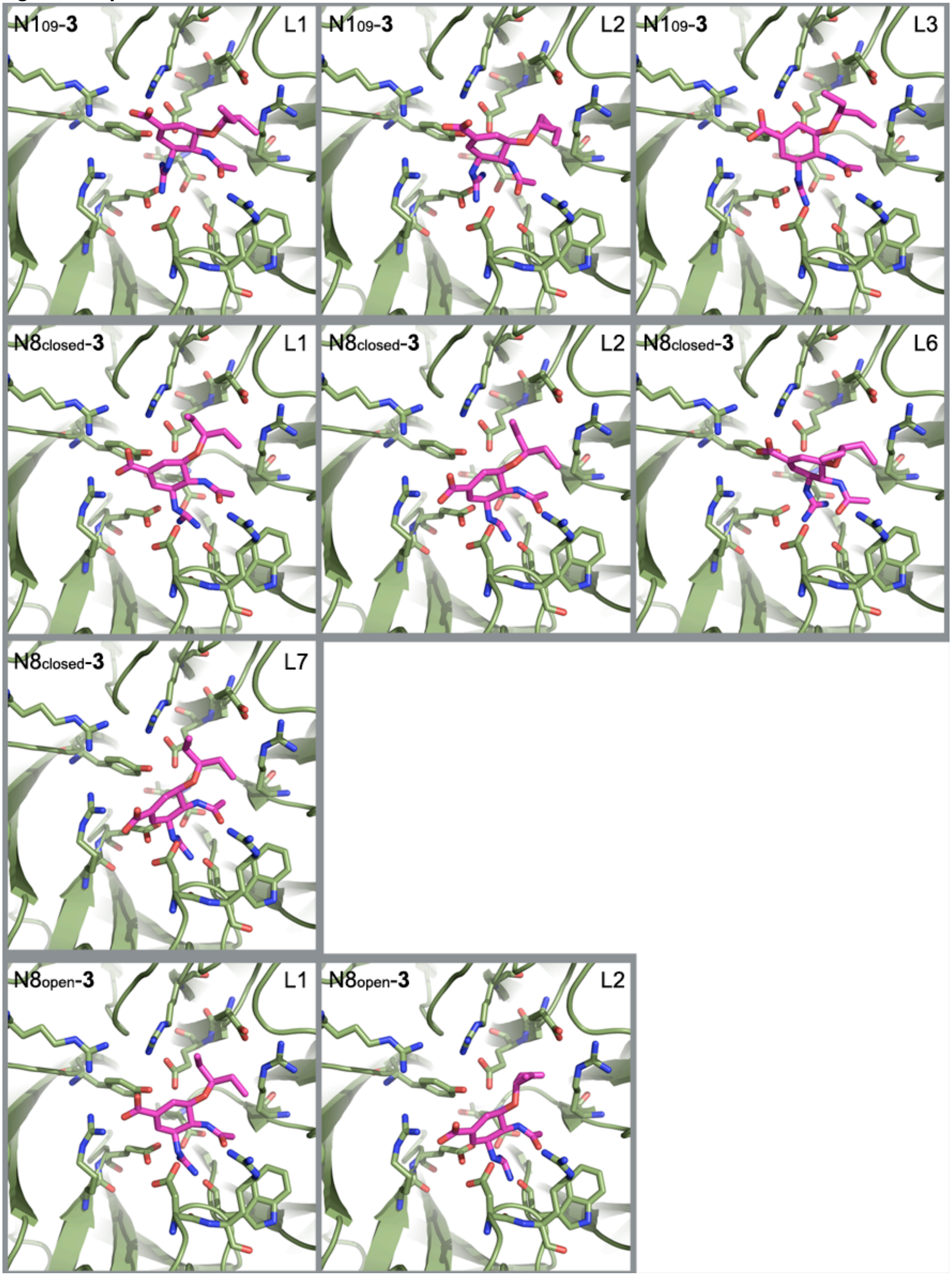

Ligand 4 Populations of Top 14 Conformations

Conformation	Neuraminidase			
	N2	$N1_{09}$	$N8_{closed}$	$N8_{open}$
**1**	98%	38%	55%	55%
**2**	2%	22%	18%	27%
**3**	0%	17%	7%	8%
**4**	0%	8%	5%	3%
**5**	0%	4%	4%	2%
**6**	0%	3%	2%	1%
**7**	0%	3%	2%	1%
**8**	0%	1%	1%	1%
**9**	0%	1%	1%	0%
**10**	0%	1%	1%	0%
**11**	0%	1%	1%	0%
**12**	0%	0%	1%	0%
**13**	0%	0%	1%	0%
**14**	0%	0%	0%	0%
**Sum**	100%	100%	100%	100%

Ligand 4 Key Cluster Structures

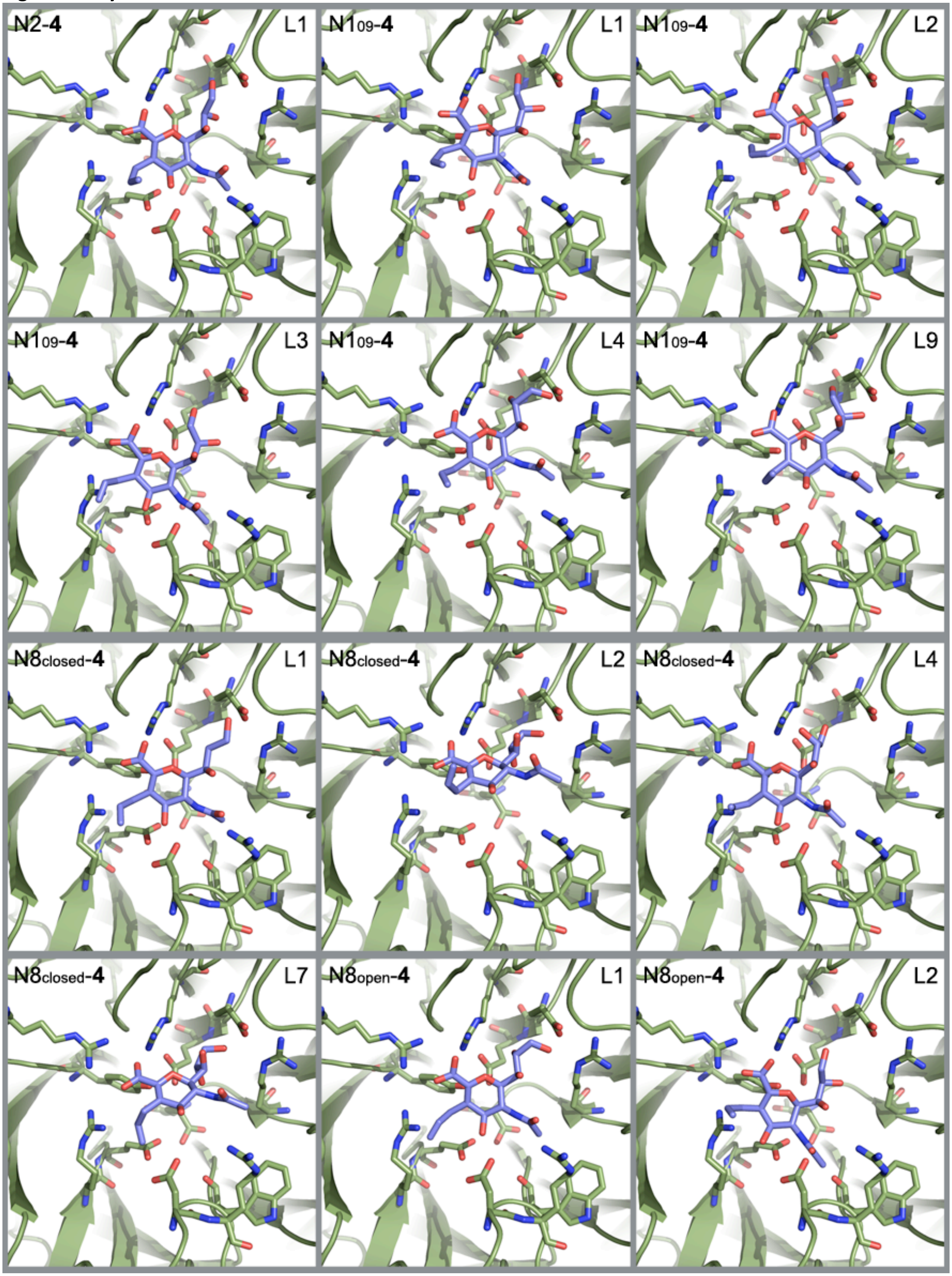

Ligand 5 Populations of Top 14 Conformations

Conformation	Neuraminidase			
	**N2**	**$N1_{09}$**	**$N8_{closed}$**	**$N8_{open}$**
**1**	27%	66%	30%	33%
**2**	23%	20%	29%	30%
**3**	10%	8%	23%	10%
**4**	8%	3%	7%	7%
**5**	7%	1%	4%	6%
**6**	5%	1%	2%	2%
**7**	5%	1%	1%	2%
**8**	5%	0%	1%	2%
**9**	3%	0%	1%	1%
**10**	2%	0%	1%	1%
**11**	1%	0%	0%	1%
**12**	1%	0%	0%	1%
**13**	1%	0%	0%	1%
**14**	1%	0%	0%	0%
**Sum**	99%	100%	100%	100%

Ligand 5 Key Cluster Structures

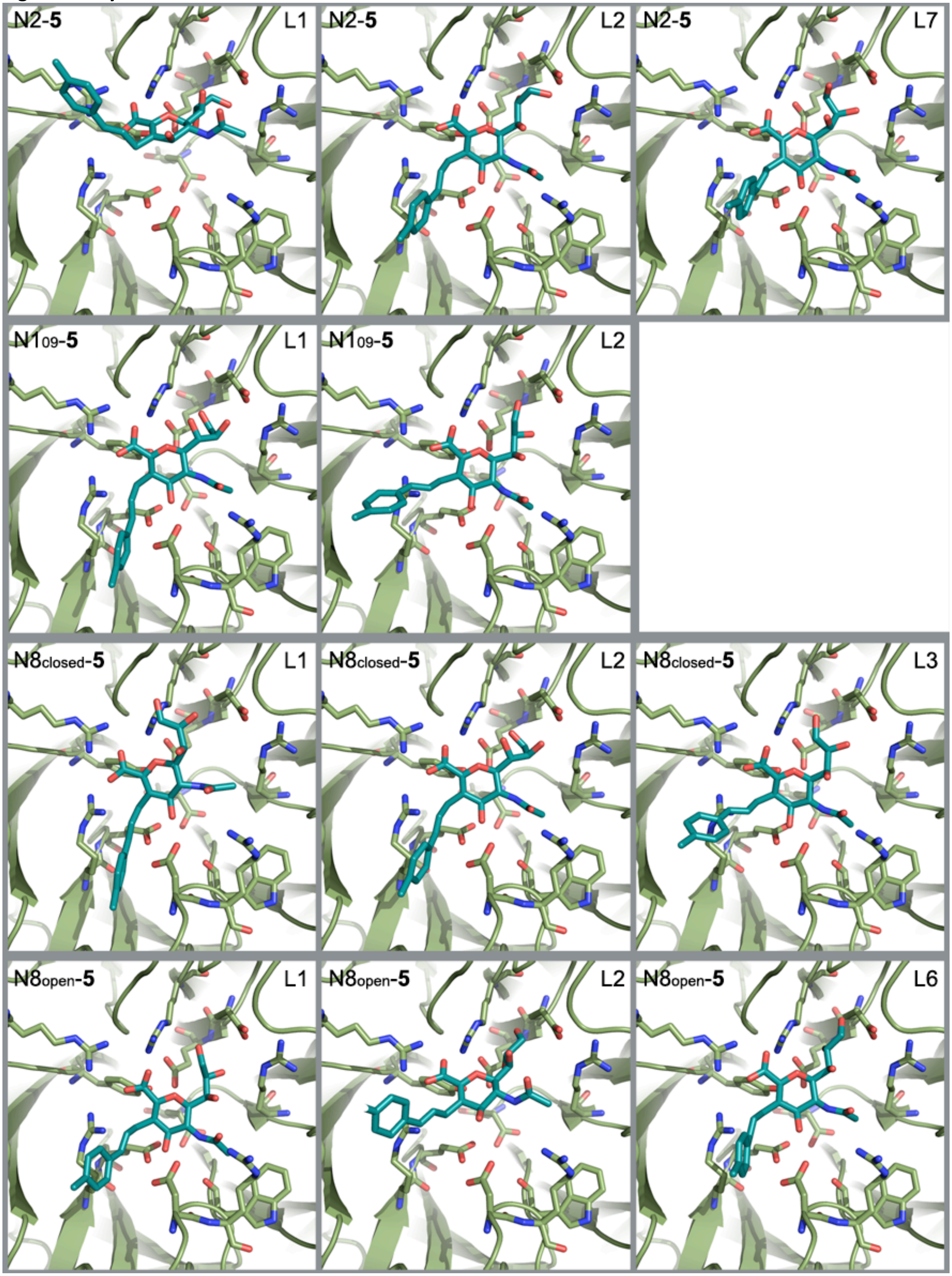

Ligand 6 Populations of Top 14 Conformations

	Neuraminidase			
**Conformation**	**N2**	**$N1_{09}$**	**$N8_{closed}$**	**$N8_{open}$**
**1**	35%	52%	53%	50%
**2**	25%	10%	9%	17%
**3**	12%	9%	8%	10%
**4**	10%	7%	5%	7%
**5**	5%	3%	4%	5%
**6**	3%	3%	3%	2%
**7**	3%	2%	2%	2%
**8**	1%	2%	2%	2%
**9**	1%	1%	1%	1%
**10**	1%	1%	1%	1%
**11**	1%	1%	1%	1%
**12**	1%	1%	1%	1%
**13**	0%	1%	1%	1%
**14**	0%	1%	1%	0%
**Sum**	100%	94%	92%	100%

Ligand 6 Key Cluster Structures

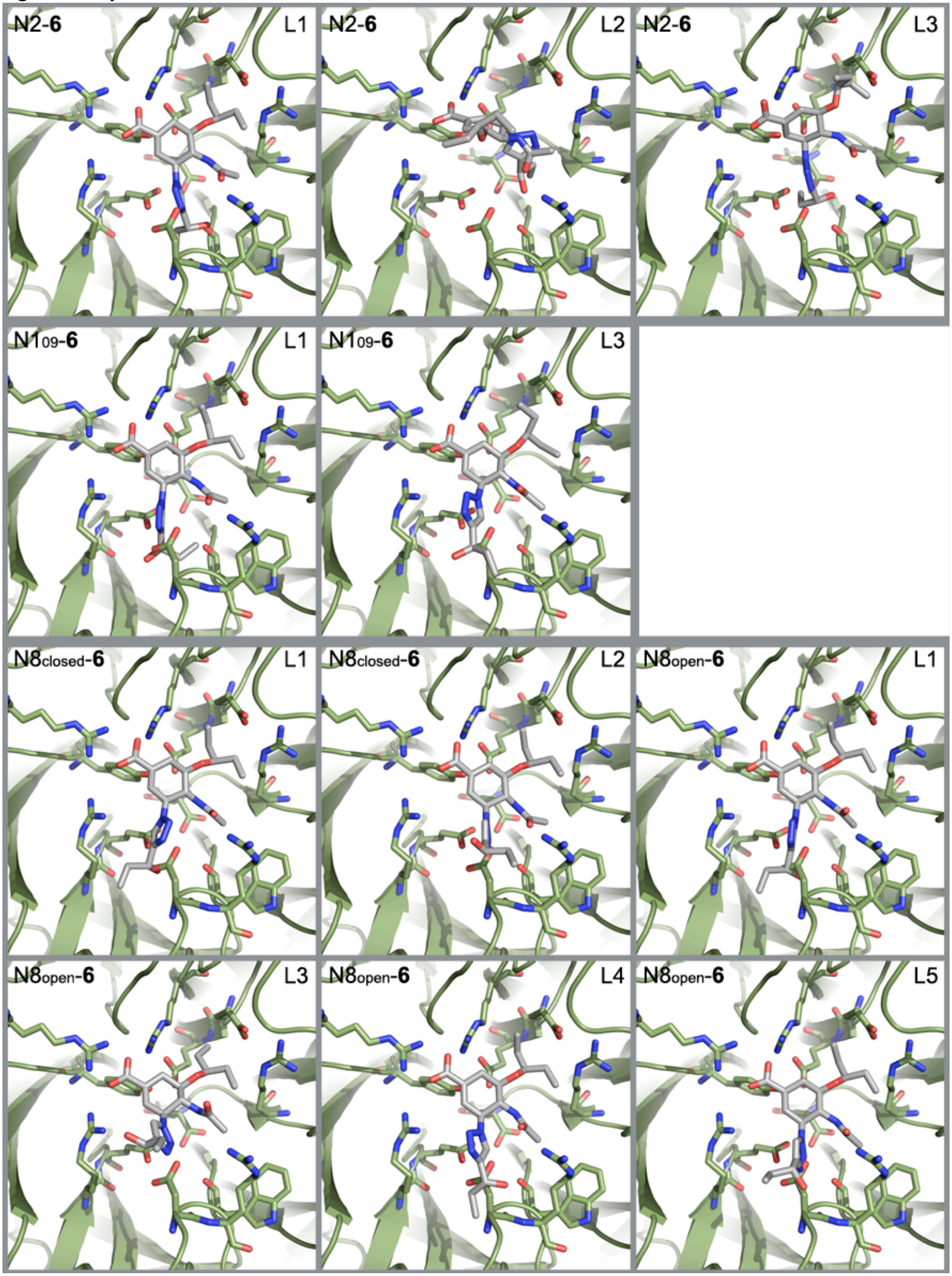

Ligand 7 Populations of Top 14 Conformations

Conformation	Neuraminidase			
	N2	$N1_{09}$	$N8_{closed}$	$N8_{open}$
1	97%	32%	61%	37%
2	2%	16%	9%	9%
3	1%	10%	5%	7%
4	0%	5%	4%	7%
5	0%	4%	3%	5%
6	0%	3%	3%	5%
7	0%	2%	2%	5%
8	0%	2%	2%	3%
9	0%	2%	2%	2%
10	0%	2%	1%	2%
11	0%	1%	1%	2%
12	0%	1%	1%	1%
13	0%	1%	0%	1%
14	0%	1%	0%	1%
Sum	100%	83%	100%	87%

Ligand 7 Key Cluster Structures

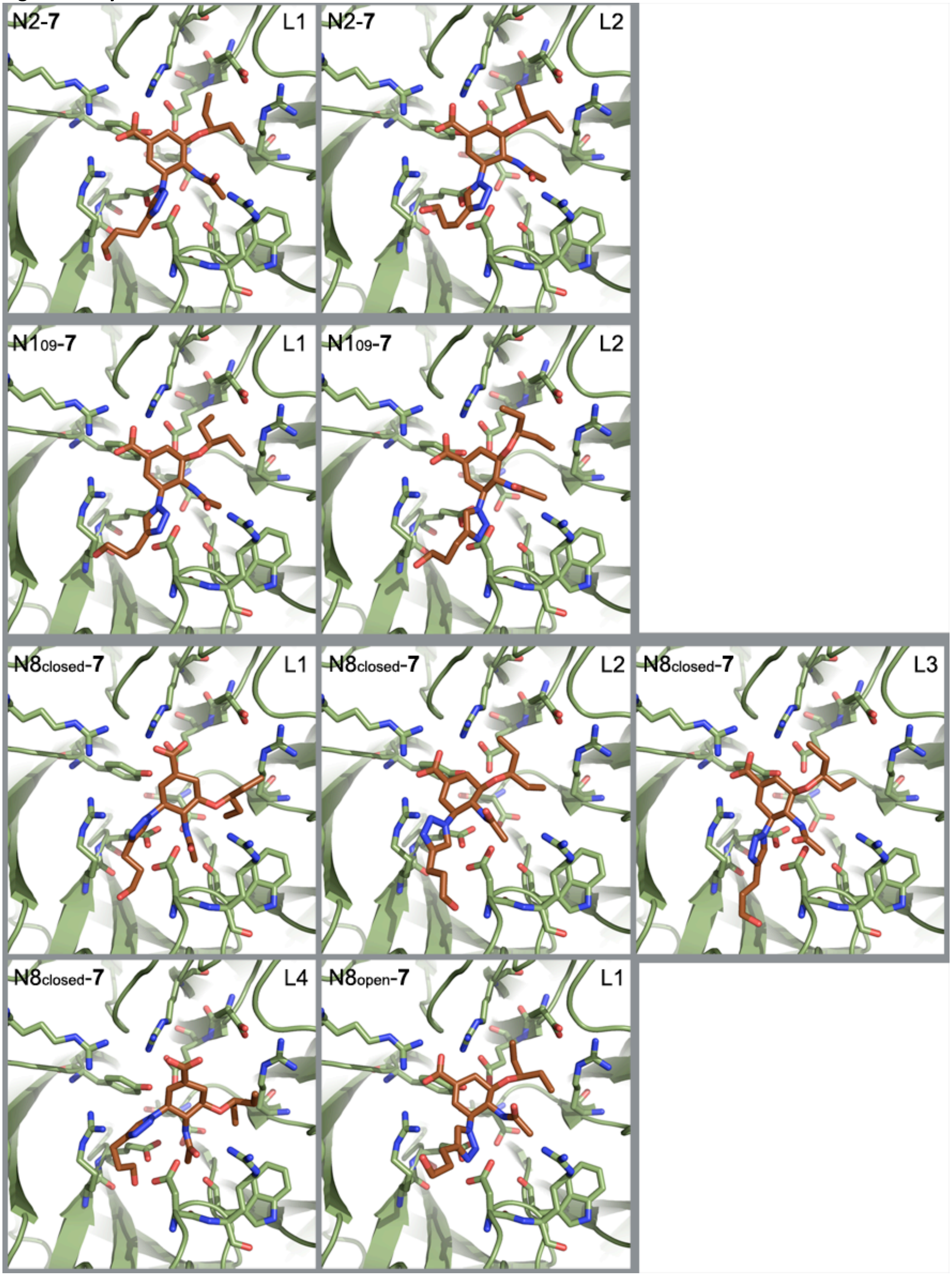

Supplement: Text S4 — Key ligand conformations. (PDF) [file pone.0059873.s005.pdf]
