# Supplementary material for: The Influence of 150-Cavity Binders on the Dynamics of Influenza A Neuraminidases as Revealed by Molecular Dynamics Simulations and Combined Clustering
Source: PLoS One. 2013 Mar 27;8(3):e59873. doi: 10.1371/journal.pone.0059873 (PMC3609799; doi:10.1371/journal.pone.0059873)
Supplement: Text S5 — Simulation stability over time from 20 ns blocks of cluster populations. (PDF) [file pone.0059873.s006.pdf]

Supporting Information
Text S5. Simulation stability over time from 20 ns blocks of cluster populations.

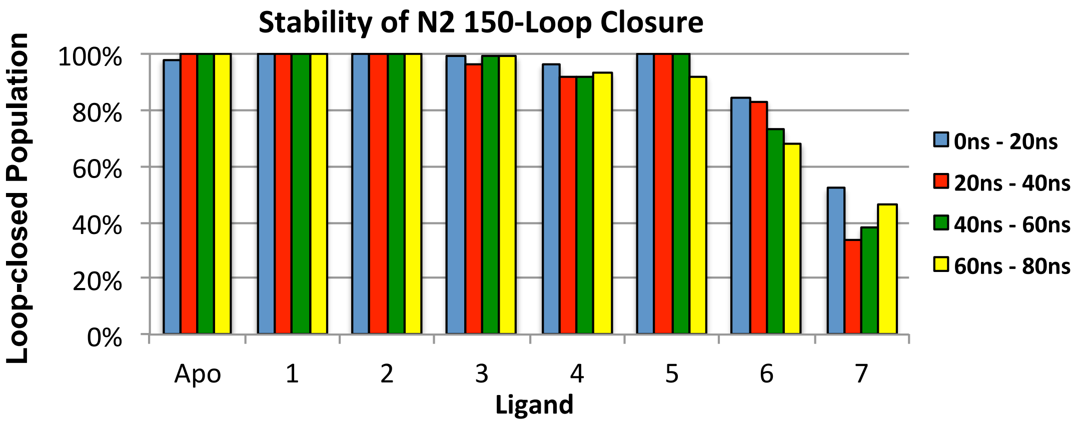

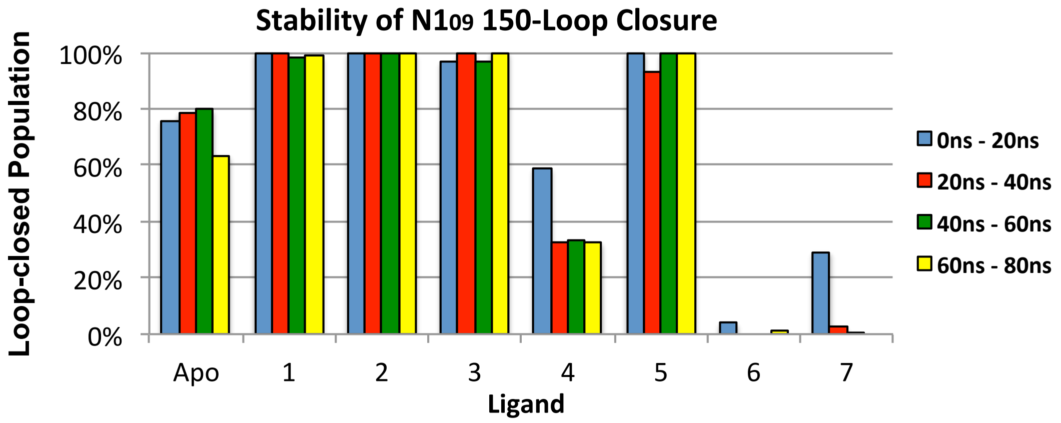

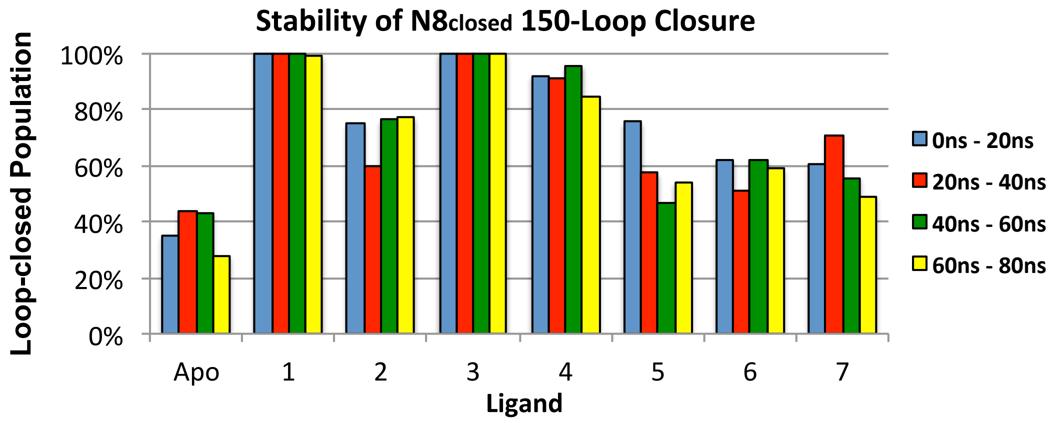

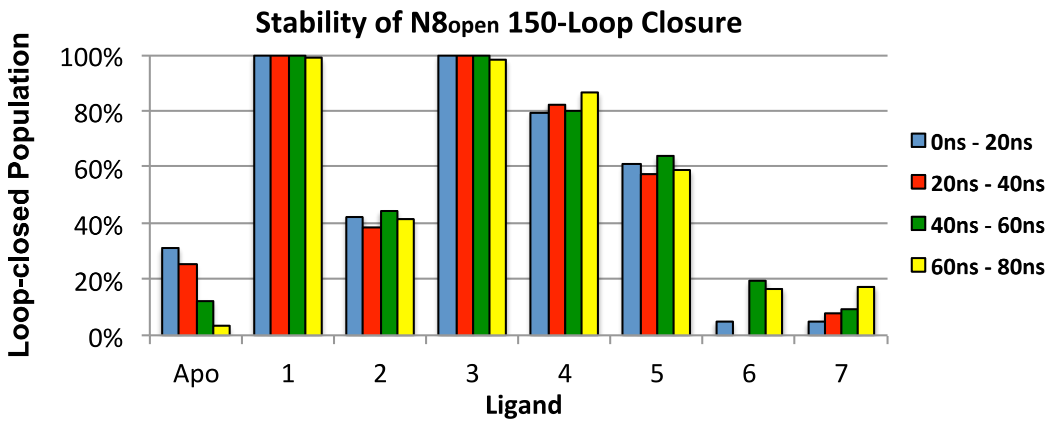

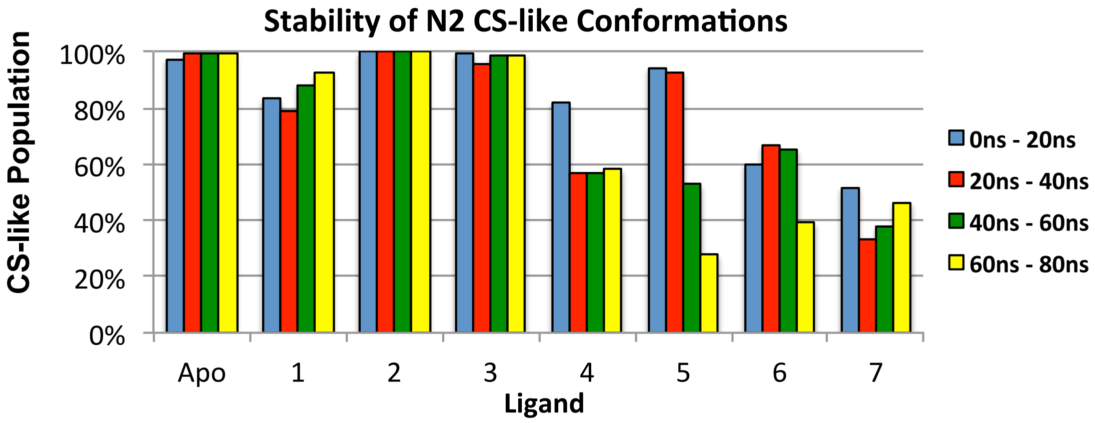
Stability of N2 CS-like Conformations
CS-like Population
100%
80%
60%
40%
20%
0%
Apo
1
2
3
4
5
6
7
Ligand
0ns - 20ns
20ns - 40ns
40ns - 60ns
60ns - 80ns

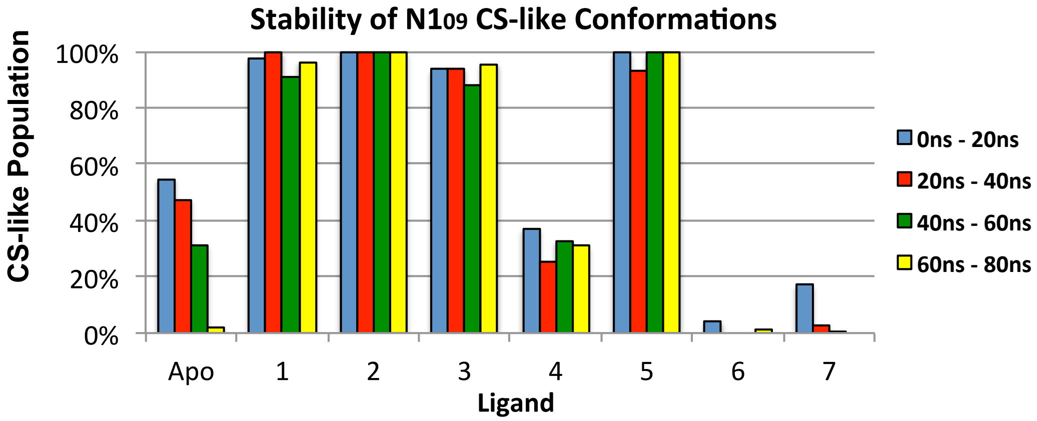
Stability of N109 CS-like Conformations
CS-like Population
100%
80%
60%
40%
20%
0%
Apo
1
2
3
4
5
6
7
Ligand
0ns - 20ns
20ns - 40ns
40ns - 60ns
60ns - 80ns

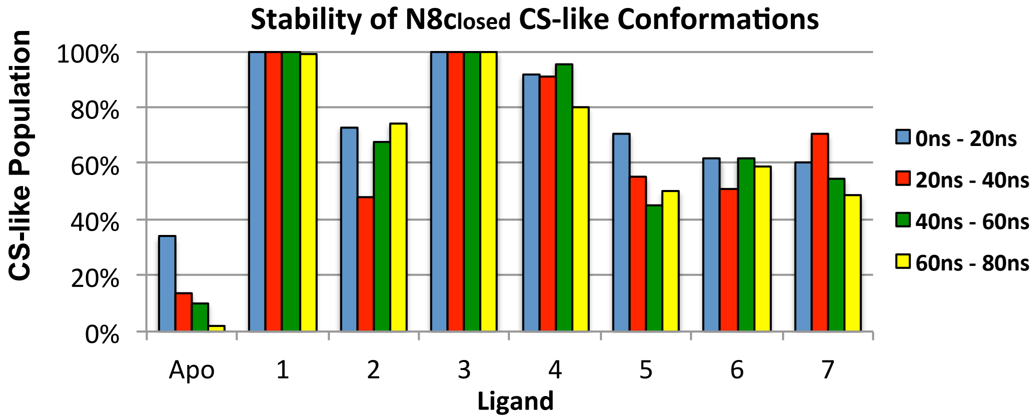
Stability of N8Closed CS-like Conformations
CS-like Population
100%
80%
60%
40%
20%
0%
Apo
1
2
3
4
5
6
7
Ligand
0ns - 20ns
20ns - 40ns
40ns - 60ns
60ns - 80ns

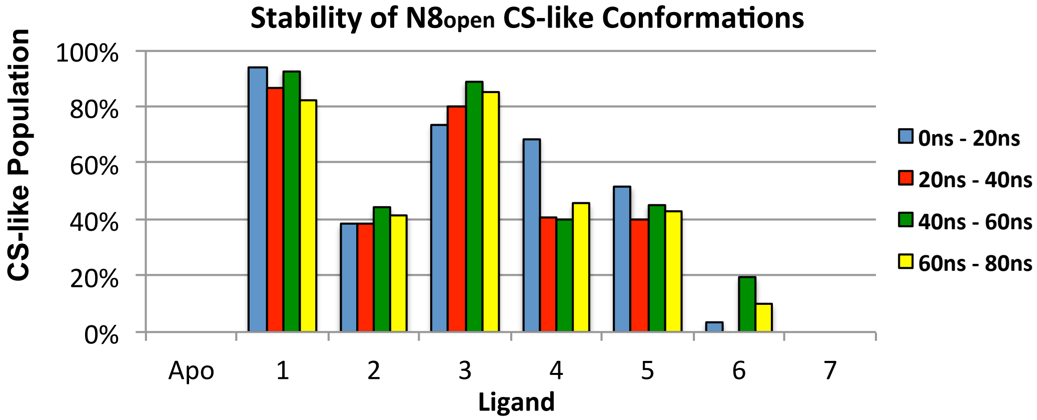
Stability of N8open CS-like Conformations
CS-like Population
100%
80%
60%
40%
20%
0%
Apo
1
2
3
4
5
6
7
Ligand
0ns - 20ns
20ns - 40ns
40ns - 60ns
60ns - 80ns

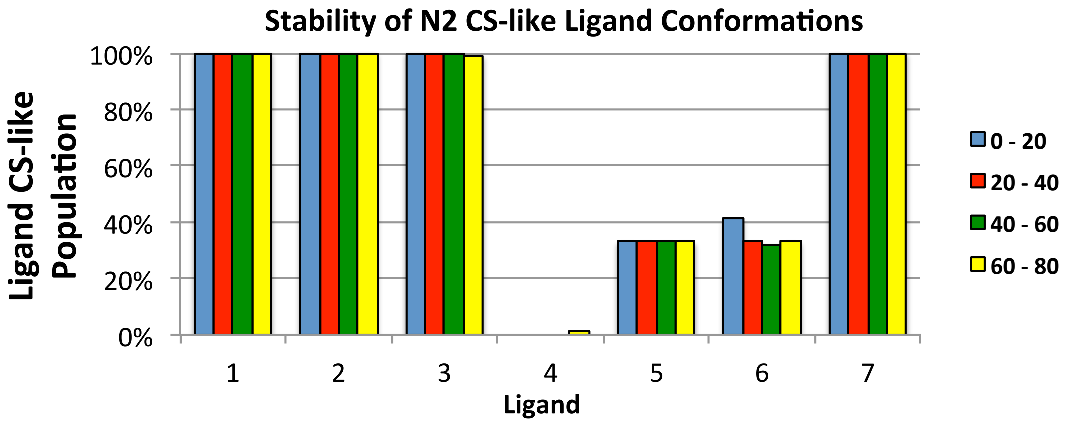
Stability of N2 CS-like Ligand Conformations
Ligand CS-like Population
100%
80%
60%
40%
20%
0%
1
2
3
4
5
6
7
Ligand
0 - 20
20 - 40
40 - 60
60 - 80

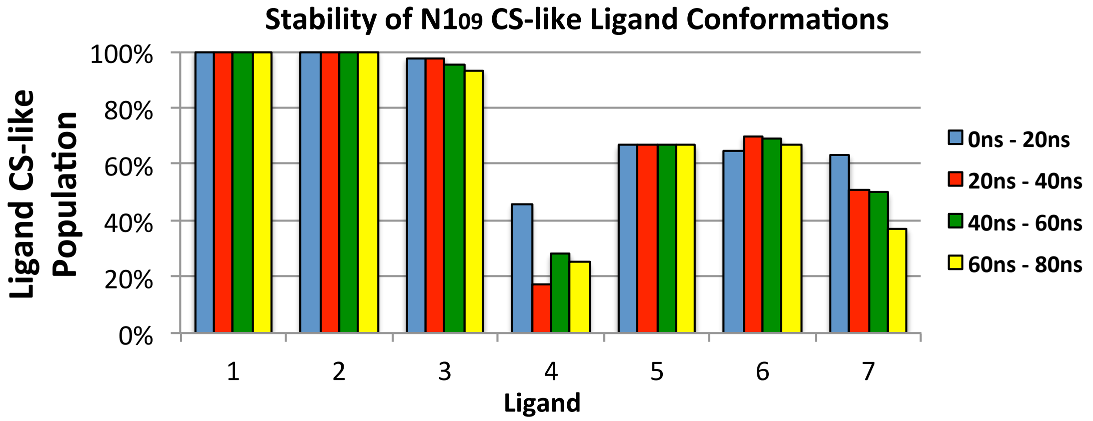
Stability of N109 CS-like Ligand Conformations
Ligand CS-like Population
100%
80%
60%
40%
20%
0%
1
2
3
4
5
6
7
Ligand
0ns - 20ns
20ns - 40ns
40ns - 60ns
60ns - 80ns

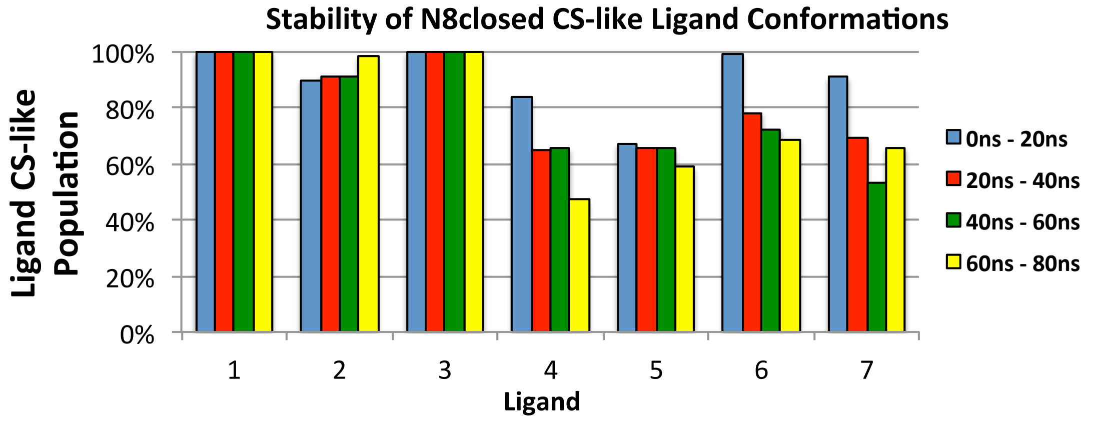
Stability of N8closed CS-like Ligand Conformations
Ligand CS-like Population
100%
80%
60%
40%
20%
0%
1
2
3
4
5
6
7
Ligand
0ns - 20ns
20ns - 40ns
40ns - 60ns
60ns - 80ns

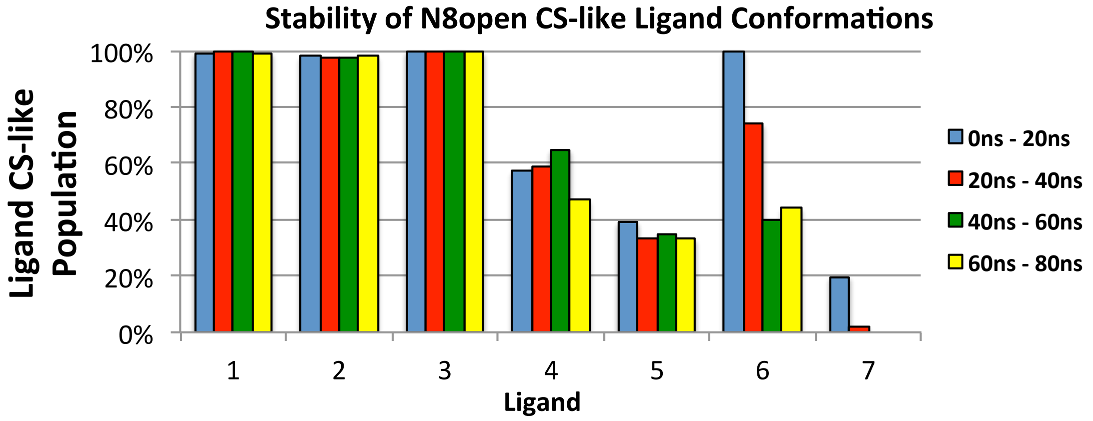
Stability of N8open CS-like Ligand Conformations
Ligand CS-like Population
100%
80%
60%
40%
20%
0%
1
2
3
4
5
6
7
Ligand
0ns - 20ns
20ns - 40ns
40ns - 60ns
60ns - 80ns
