# Supplementary material for: The Influence of 150-Cavity Binders on the Dynamics of Influenza A Neuraminidases as Revealed by Molecular Dynamics Simulations and Combined Clustering
Source: PLoS One. 2013 Mar 27;8(3):e59873. doi: 10.1371/journal.pone.0059873 (PMC3609799; doi:10.1371/journal.pone.0059873)

Supporting Information

Text S5. Atomic Charges and Atom Types.

(Note: Ligands are numbered as in paper and only one set of parameters is given for chemically identical atoms.)

Ligand 1

Atom	RESP Charge	GAFF Atom-Type
C1	0.1434	ce
C2	-0.4101	c2
C3	0.1901	c3
C4	0.0801	c3
C5	-0.0045	c3
O6	-0.1722	os
C7	0.7210	c
O8	-0.7106	o
N9	-0.6344	n
H10	0.3320	hn
C11	0.6730	c
O12	-0.5875	o
C13	-0.2707	c3
H14	0.0818	hc
H15	0.1664	h1
H17	0.1588	ha
N18	-0.5396	nh
H19	0.0819	h1
H20	0.1176	h1
H34	0.3141	hn
C35	0.9047	cz
N36	-0.9369	nh
H37	0.4433	hn
C62	0.0089	c3
O63	-0.5604	oh
H64	0.3755	ho
H65	0.1794	h1
C66	0.1635	c3
O67	-0.6270	oh
H68	0.4080	ho
H69	0.0806	h1
C70	0.1552	c3
H71	0.0391	h1
O72	-0.7010	oh
H73	0.4514	ho

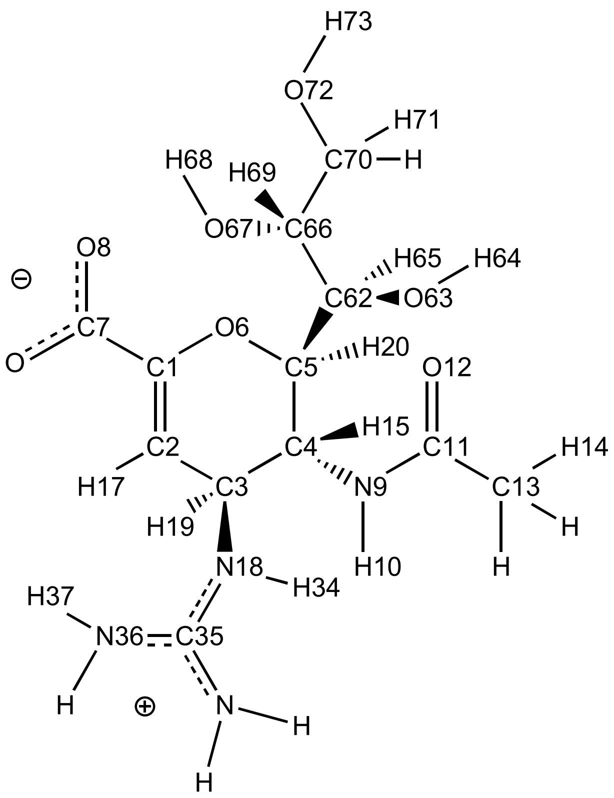

Ligand 2

Atom	RESP Charge	GAFF Atom-Type
C1	-0.0703	ce
C2	-0.0285	c3
C3	0.1050	c3
C4	-0.0024	c3
C5	0.0295	c3
C6	-0.2786	c2
C7	0.8031	c
O8	-0.7575	o
N9	-0.6654	n
H10	0.3547	hn
C11	0.6949	c
O12	-0.5959	o
C13	-0.3411	c3
H14	0.1032	hc
H15	0.1707	h1
H16	0.1915	ha
H17	0.0436	hc
N18	-0.5166	n4
H19	0.1596	hx
H20	0.1514	h1
H38	0.3538	hn
O55	-0.2939	os
C56	-0.0080	c3
H57	0.0907	h1
C58	-0.0114	c3
H59	0.0340	hc
C60	-0.0760	c3
H61	0.0242	hc

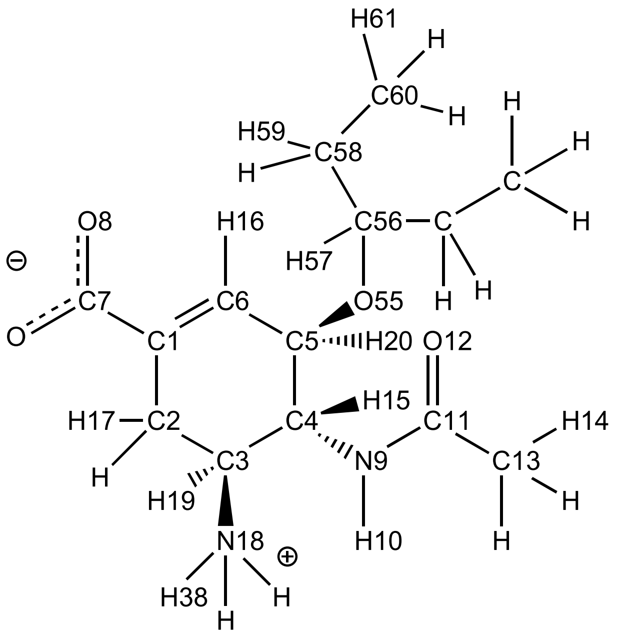

Ligand 3

Atom	RESP Charge	GAFF Atom-Type
C1	-0.0454	ce
C2	-0.3841	c2
C3	0.1278	c3
C4	0.0214	c3
C5	-0.0212	c3
C6	-0.0369	c3
C7	0.8045	c
O8	-0.7603	o
N9	-0.5626	n
H10	0.3182	hn
C11	0.6376	c
O12	-0.5828	o
C13	-0.1542	c3
H14	0.0482	hc
H15	0.1779	h1
H16	0.0985	hc
H17	0.1601	ha
N18	-0.5529	nh
H19	0.1113	h1
H20	0.0864	h1
H34	0.3712	hn
C35	0.8336	cz
N36	-0.8944	nh
H37	0.4415	hn
O55	-0.2476	os
C56	0.0215	c3
H57	0.0726	h1
C58	-0.0317	c3
H59	0.0308	hc
C60	-0.0432	c3
H61	0.0120	hc

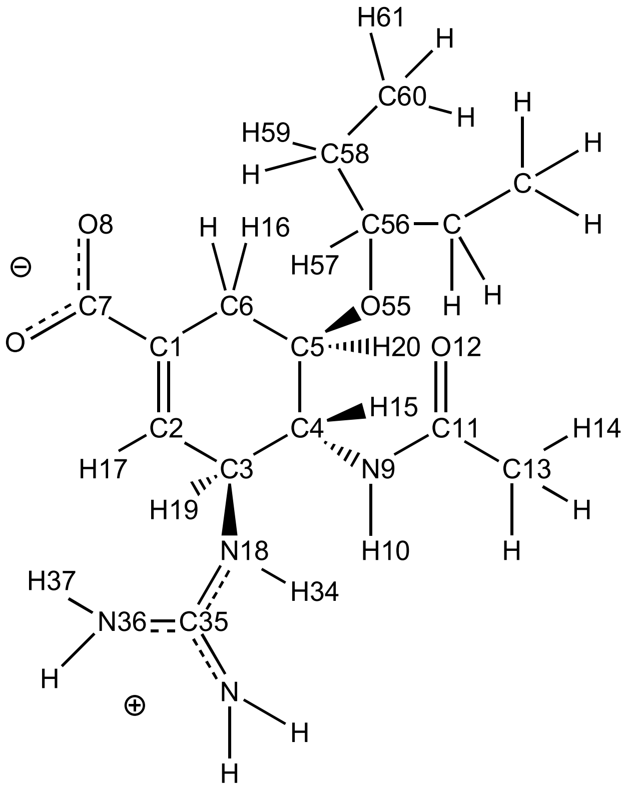

Ligand 4

Atom	RESP Charge	GAFF Atom-Type
C1	0.0224	ce
C2	-0.0752	c2
C3	0.0268	c3
C4	0.0946	c3
C5	-0.0041	c3
O6	-0.2184	os
C7	0.7697	c
O8	-0.7522	o
N9	-0.5948	n
H10	0.3130	hn
C11	0.7021	c
O12	-0.6069	o
C13	-0.2633	c3
H14	0.0713	hc
H15	0.1730	h1
H19	0.0809	h1
H20	0.1122	h1
O39	0.5786	oh
H40	0.3778	ho
C41	-0.0592	c3
H42	0.0859	hc
C43	-0.0623	c2
H44	0.1335	ha
C45	-0.4787	c2
H46	0.1500	ha
C62	0.0208	c3
O63	-0.5845	oh
H64	0.3948	ho
H65	0.1186	h1
C66	0.1318	c3
O67	-0.6233	oh
H68	0.3982	ho
H69	0.0816	h1
C70	0.1381	c3
H71	0.0458	h1
O72	-0.6237	oh
H73	0.4102	ho

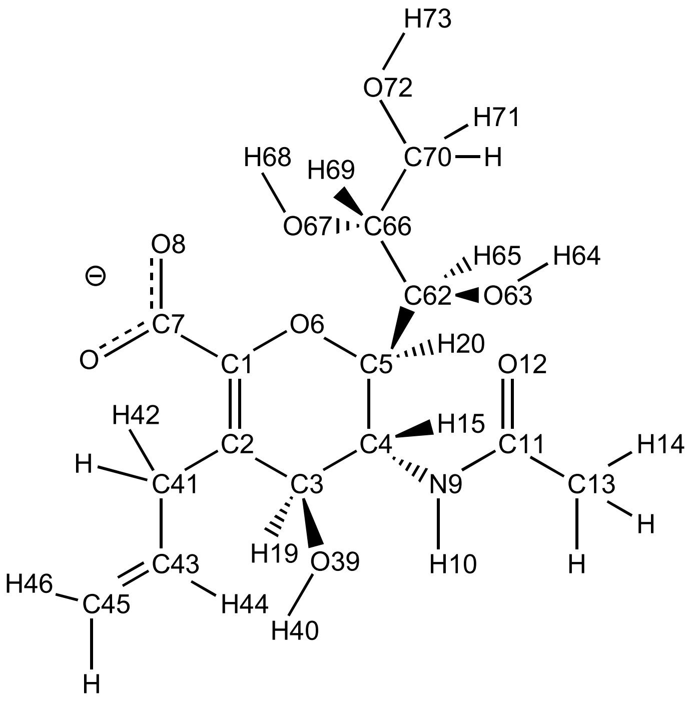

Ligand 5

Atom	RESP Charge	GAFF Atom-Type
C1	0.0263	ce
C2	-0.0422	c2
C3	-0.0082	c3
C4	0.0857	c3
C5	-0.0124	c3
O6	-0.2134	os
C7	0.7654	c
O8	-0.7504	o
N9	-0.5664	n
H10	0.3088	hn
C11	0.6902	c
O12	-0.6056	o
C13	-0.2639	c3
H14	0.0718	hc
H15	0.1763	h1
H19	0.0836	h1
H20	0.1178	h1
O39	-0.5653	oh
H40	0.3740	ho
C41	-0.1562	c3
H42	0.1063	hc
C43	-0.1068	c2
H44	0.1650	ha
C45	-0.2089	ce
H46	0.1137	ha
C47	0.0694	ca
C48	-0.1423	ca
H49	0.1305	ha
C50	-0.2350	ca
H51	0.1416	ha
C52	0.1169	ca
C53	-0.1750	c3
H54	0.0523	hc
C62	0.0168	c3
O63	-0.5845	oh
H64	0.3960	ho
H65	0.1230	h1
C66	0.1126	c3
O67	-0.6196	oh
H68	0.4004	ho
H69	0.0919	h1
C70	0.1284	c3
H71	0.0500	h1
O72	-0.6182	oh
H73	0.4107	ho

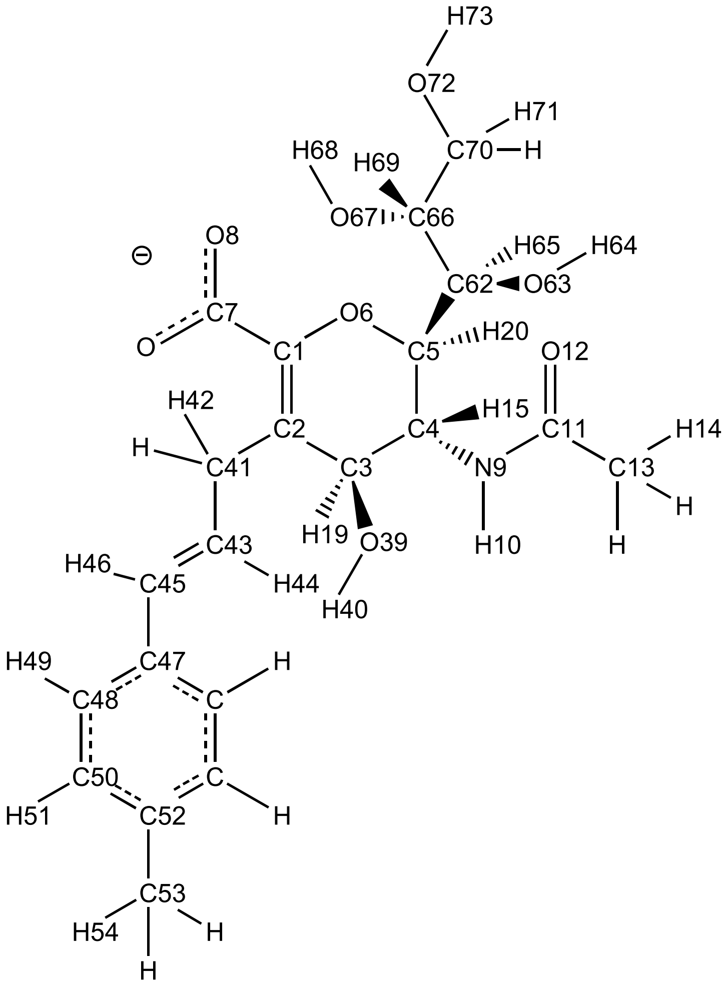

Ligand 6

Atom	RESP Charge	GAFF Atom-Type
C1	-0.0177	ce
C2	-0.4443	c2
C3	-0.0077	c3
C4	0.0188	c3
C5	-0.0056	c3
C6	-0.0610	c3
C7	0.8086	c
O8	-0.7839	o
N9	-0.5350	n
H10	0.3070	hn
C11	0.6556	c
O12	-0.5691	o
C13	-0.3067	c3
H14	0.0826	hc
H15	0.1898	h1
H16	0.0885	hc
H17	0.2147	ha
N18	0.3721	na
H19	0.1083	h1
H20	0.1001	h1
N21	-0.2060	nc
N22	-0.3519	nd
C23	-0.3224	cc
H24	0.1313	h4
C25	0.2787	cd
C26	0.0149	c3
O27	-0.6326	oh
H28	0.4144	ho
H29	0.1238	h1
C30	0.0827	c3
H31	0.0026	hc
C32	-0.0524	c3
H33	0.0055	hc
O55	-0.3552	os
C56	0.0588	c3
H57	0.0931	h1
C58	-0.0172	c3
H59	0.0325	hc
C60	-0.0753	c3
H61	0.0117	hc

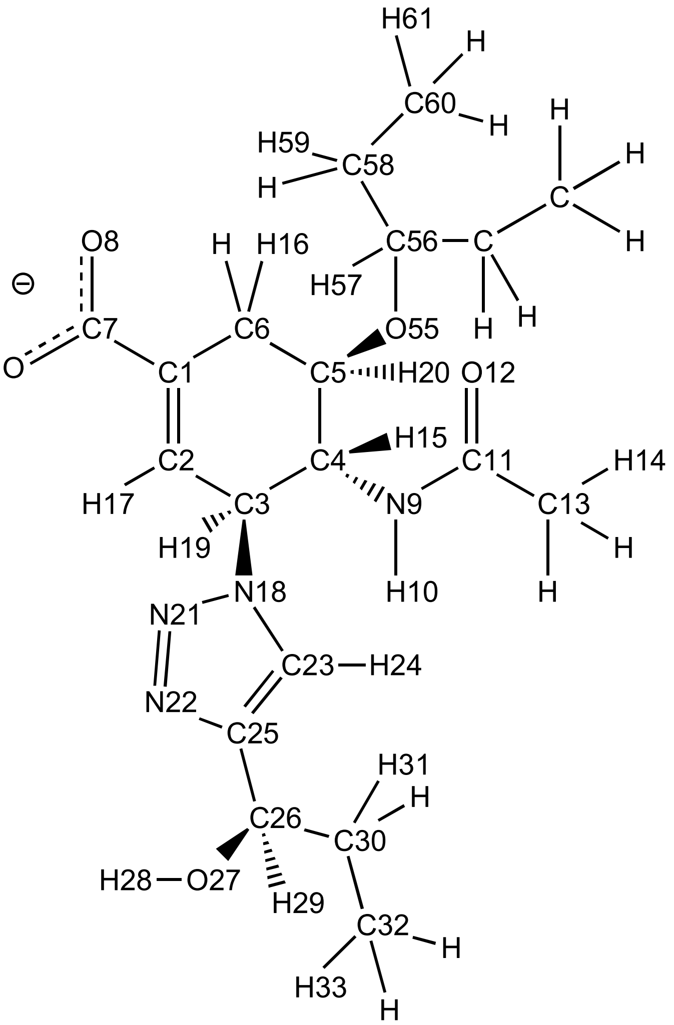

Ligand 7

Atom	RESP Charge	GAFF Atom-Type
C1	-0.0004	ce
C2	-0.4607	c2
C3	-0.0310	c3
C4	-0.0064	c3
C5	0.0227	c3
C6	-0.1209	c3
C7	0.8021	c
O8	-0.7826	o
N9	-0.4768	n
H10	0.2956	hn
C11	0.5951	c
O12	-0.5517	o
C13	-0.1768	c3
H14	0.0476	hc
H15	0.1776	h1
H16	0.0975	hc
H17	0.2157	ha
N18	0.4088	na
H19	0.1154	h1
H20	0.1108	h1
N21	-0.1905	nc
N22	-0.4087	nd
C23	-0.4022	cc
H24	0.1297	h4
C25	0.4359	cd
C26	-0.3828	c3
O27	-0.7174	oh
H28	0.4406	ho
H29	0.1052	hc
C30	0.0680	c3
H31	0.0526	hc
C32	0.1566	c3
H33	0.0218	h1
O55	-0.2909	os
C56	0.0043	c3
H57	0.0905	h1
C58	0.0140	c3
H59	0.0006	hc
C60	-0.0297	c3
H61	0.0075	hc

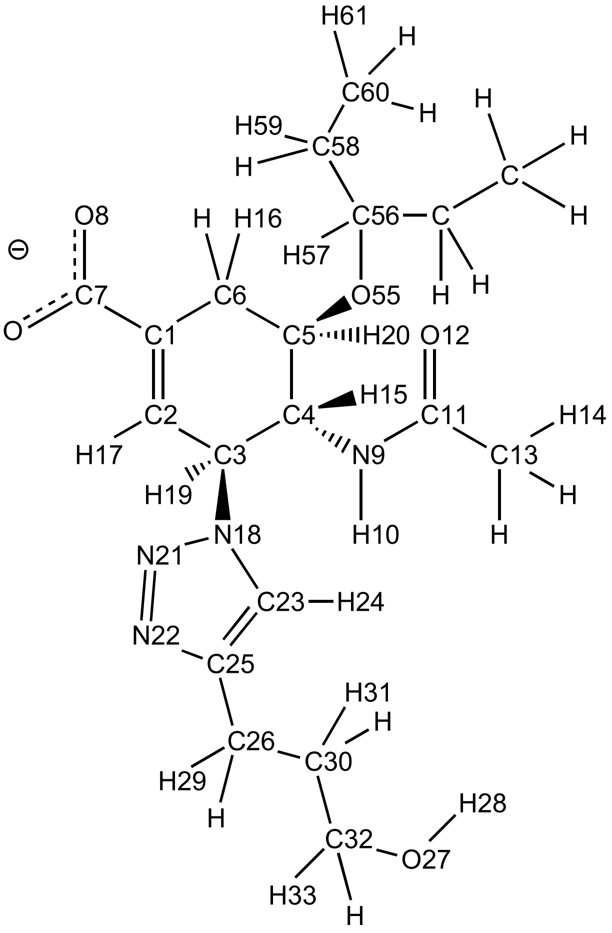

Supplement: Text S7 — Atomic charges and atom types. (PDF) [file pone.0059873.s008.pdf]
