# Supplementary material for: The Influence of 150-Cavity Binders on the Dynamics of Influenza A Neuraminidases as Revealed by Molecular Dynamics Simulations and Combined Clustering
Source: PLoS One. 2013 Mar 27;8(3):e59873. doi: 10.1371/journal.pone.0059873 (PMC3609799; doi:10.1371/journal.pone.0059873)

Supporting Information
Text S8

Root Mean Squared Deviation of All Heavy NA Atoms for N2 Systems

All values relative to post-minimization structre (pre-equilibration) common to the triplicate runs.

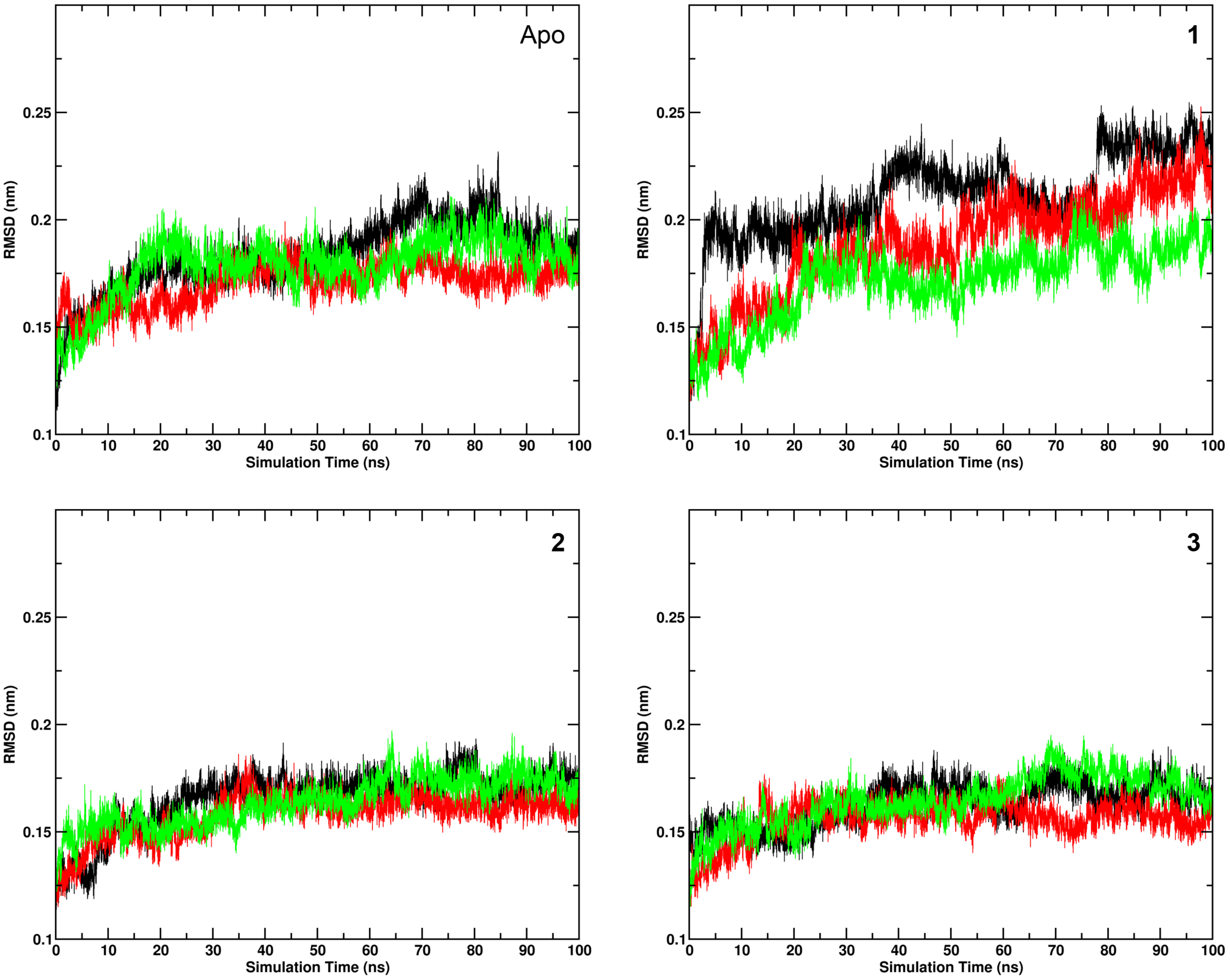

Supporting Information
Text S8

Root Mean Squared Deviation of All Heavy NA Atoms for N2 Systems

All values relative to post-minimization structre (pre-equilibration) common to the triplicate runs.

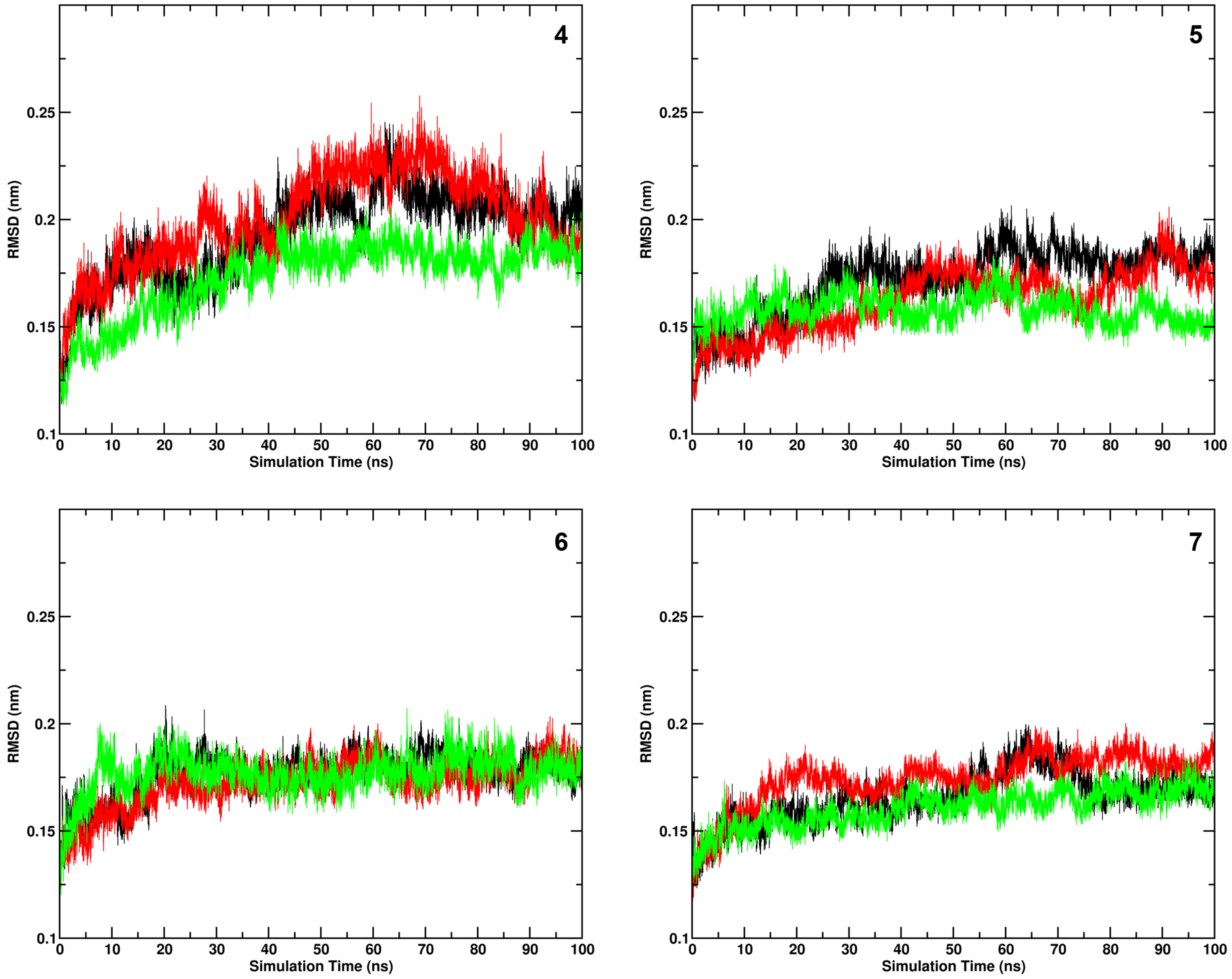

Supporting Information
Text S8

Root Mean Squared Deviation of All Heavy NA Atoms for N1$_{09}$ Systems

All values relative to post-minimization structre (pre-equilibration)
common to the triplicate runs.

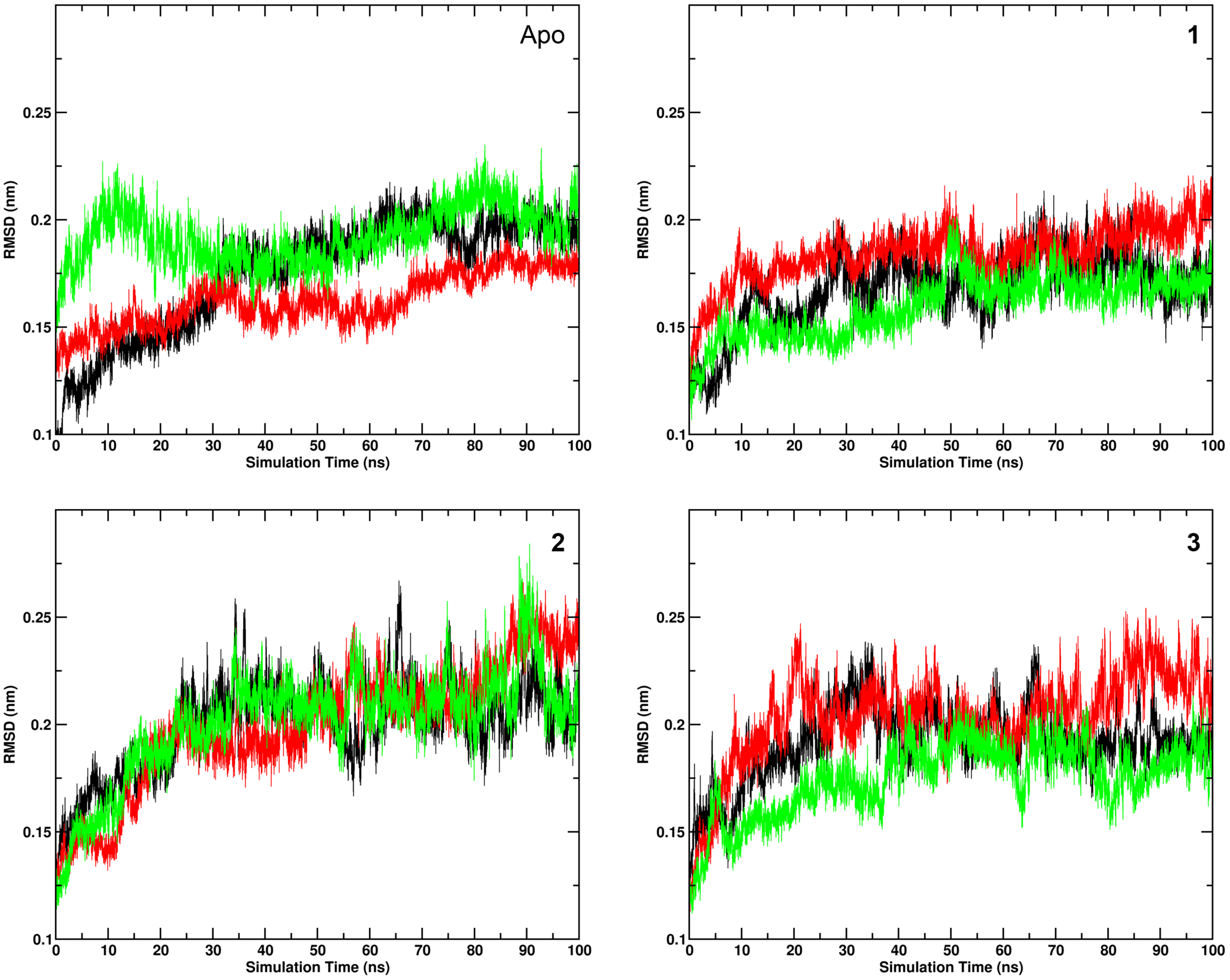

Supporting Information
Text S8

Root Mean Squared Deviation of All Heavy NA Atoms for N1$_{09}$ Systems

All values relative to post-minimization structre (pre-equilibration)
common to the triplicate runs.

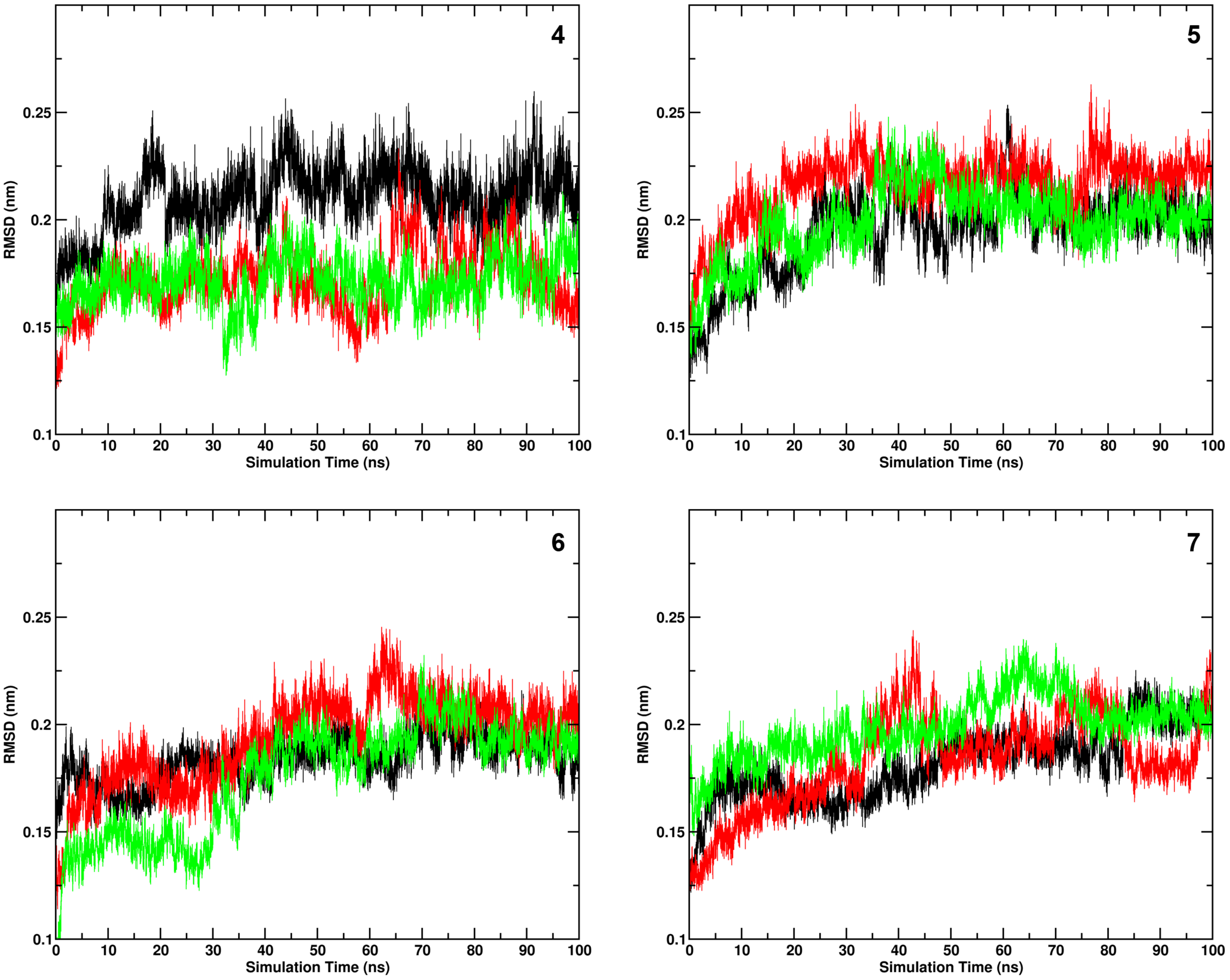

Supporting Information
Text S8

Root Mean Squared Deviation of All Heavy NA Atoms for $N8_{closed}$ Systems

All values relative to post-minimization structure (pre-equilibration) common to the triplicate runs.

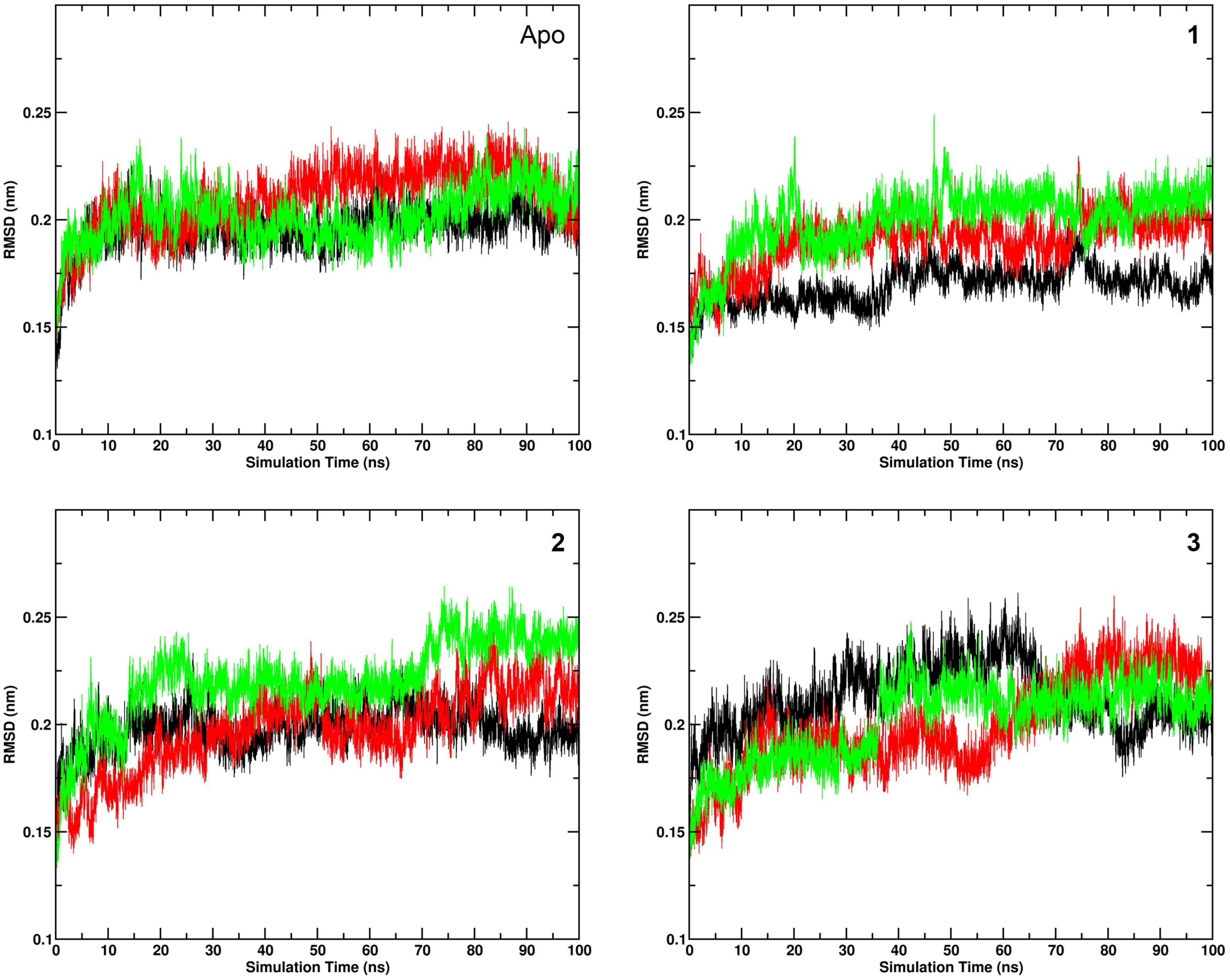

Supporting Information
Text S8

Root Mean Squared Deviation of All Heavy NA Atoms for N8$_{closed}$ Systems

All values relative to post-minimization structre (pre-equilibration)
common to the triplicate runs.

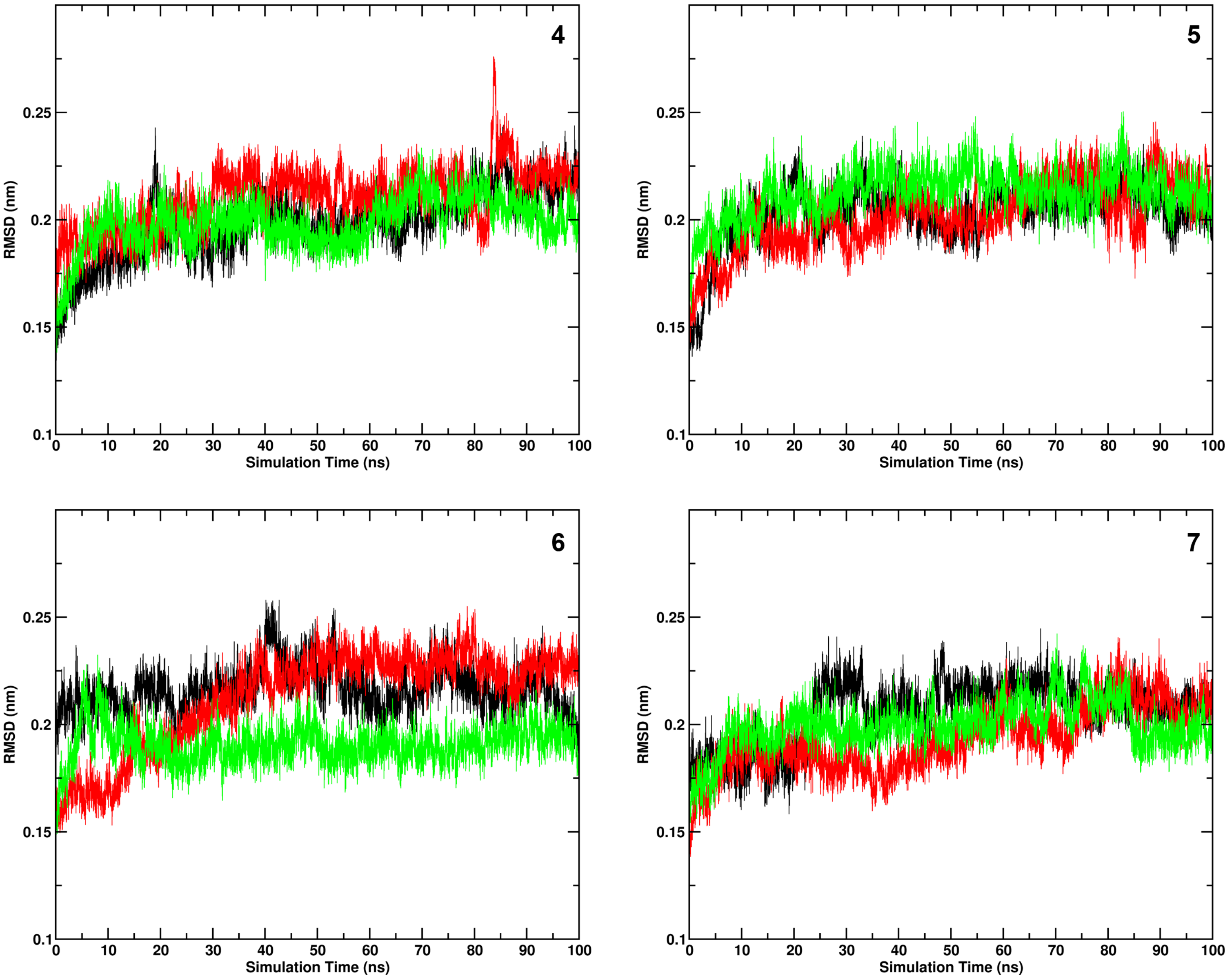

Supporting Information
Text S8

Root Mean Squared Deviation of All Heavy NA Atoms for N8$_{open}$ Systems

All values relative to post-minimization structre (pre-equilibration) common to the triplicate runs.

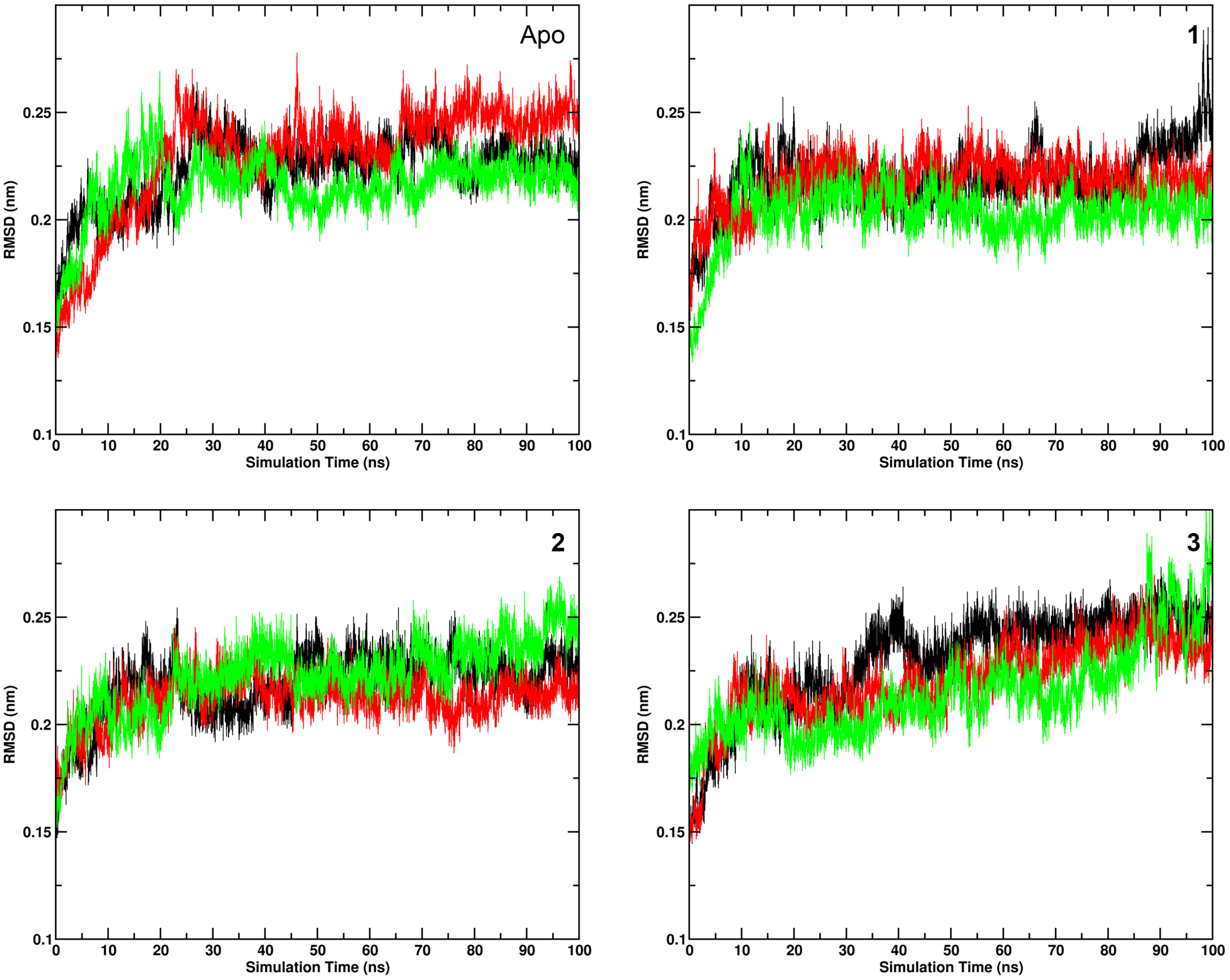

Supporting Information
Text S8

Root Mean Squared Deviation of All Heavy NA Atoms for N8$_{open}$ Systems

All values relative to post-minimization structre (pre-equilibration) common to the triplicate runs.

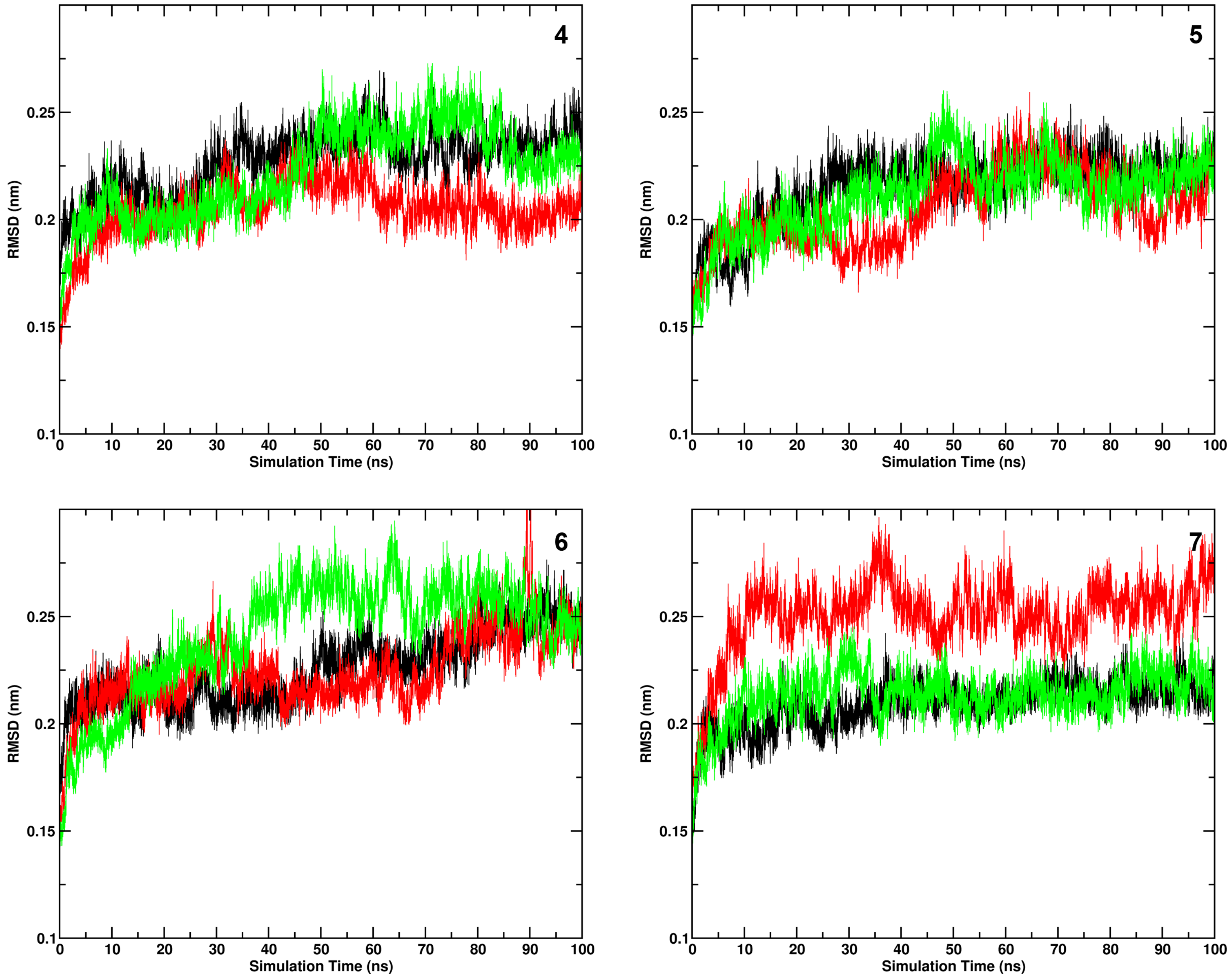

Supplement: Text S8 — Root mean squared deviation of all heavy NA atoms for all simulations (PDF) [file pone.0059873.s009.pdf]
