# Supplementary material for: The Influence of 150-Cavity Binders on the Dynamics of Influenza A Neuraminidases as Revealed by Molecular Dynamics Simulations and Combined Clustering
Source: PLoS One. 2013 Mar 27;8(3):e59873. doi: 10.1371/journal.pone.0059873 (PMC3609799; doi:10.1371/journal.pone.0059873)

Supporting Information
Text S9

Root Mean Squared Deviation of Heavy Active Site Atoms of N2 Systems

All values relative to post-minimization structre (pre-equilibration)
common to the triplicate runs.

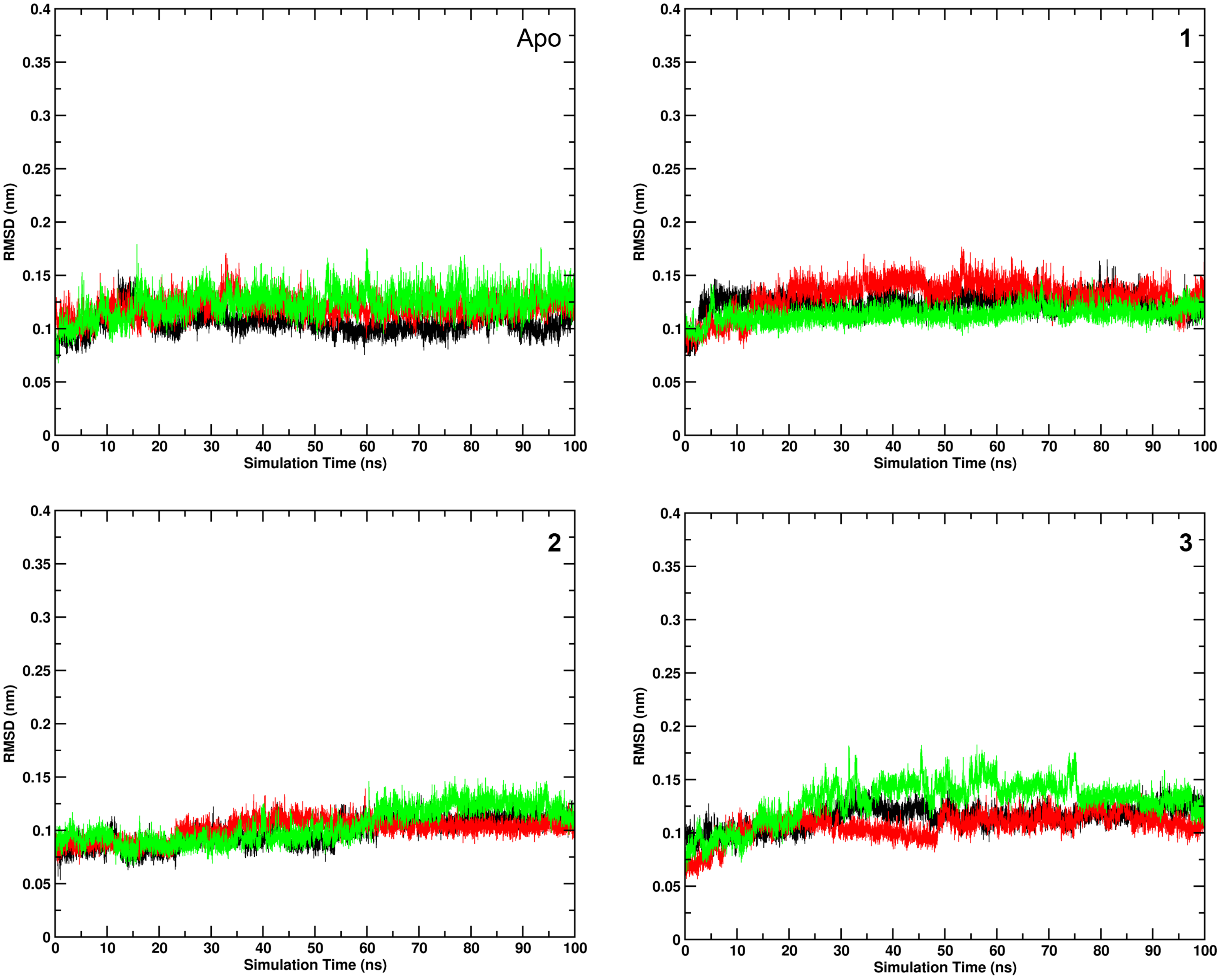

Supporting Information
Text S9

Root Mean Squared Deviation of Heavy Active Site Atoms of N2 Systems

All values relative to post-minimization structre (pre-equilibration)
common to the triplicate runs.

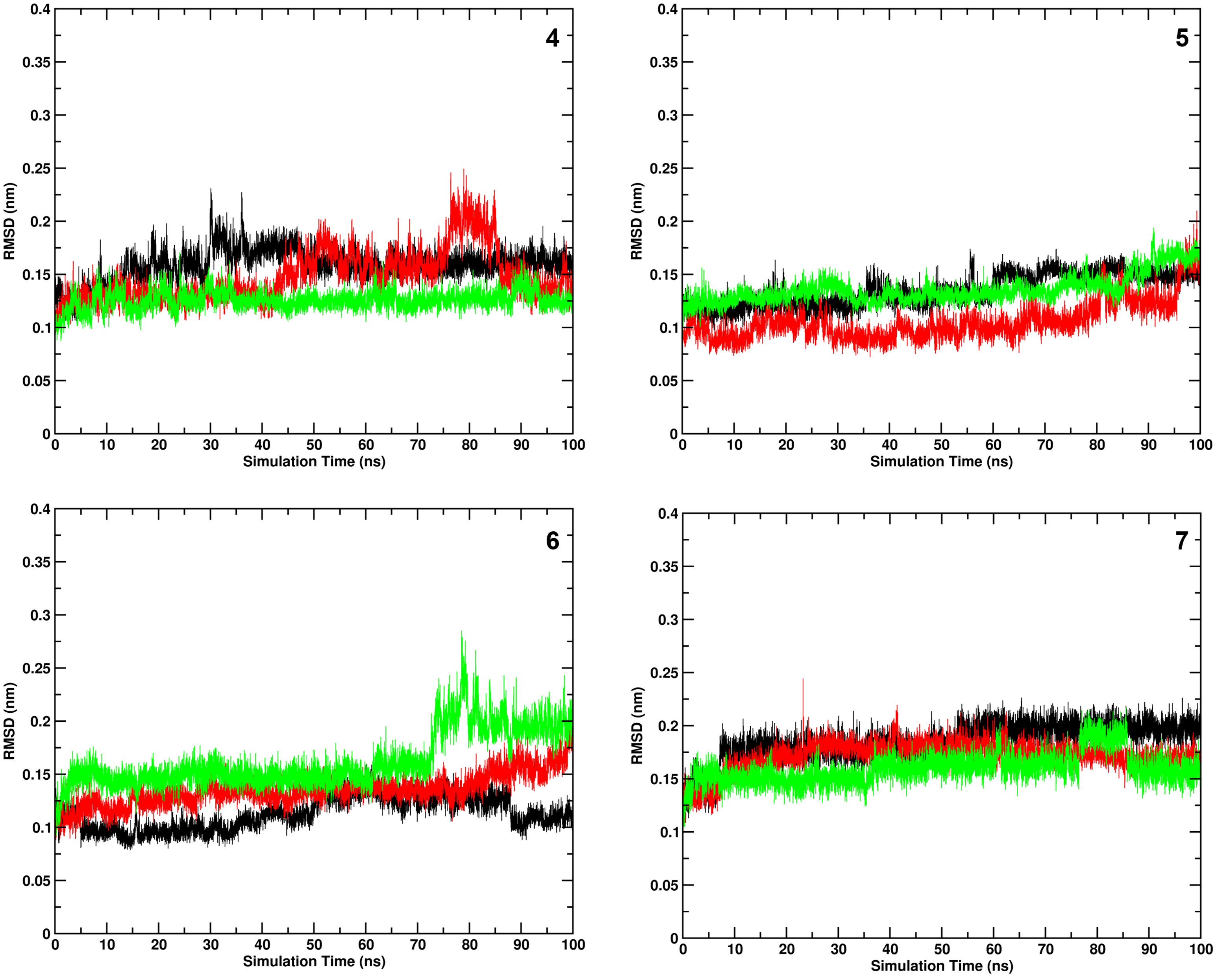

Supporting Information
Text S9

Root Mean Squared Deviation of Heavy Active Site Atoms for N1$_{09}$ Systems

All values relative to post-minimization structre (pre-equilibration) common to the triplicate runs.

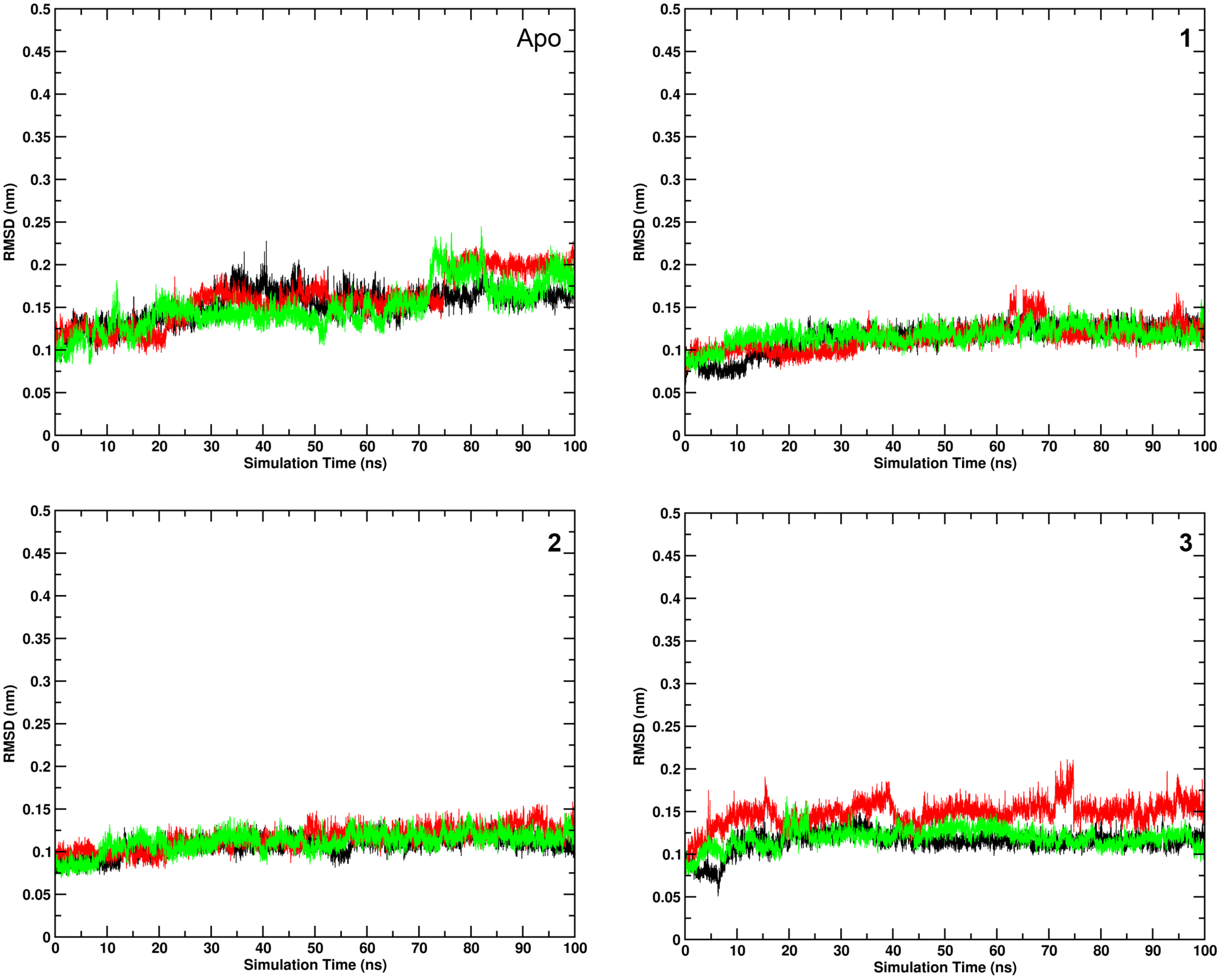

Supporting Information
Text S9

Root Mean Squared Deviation of Heavy Active Site Atoms for N109 Systems

All values relative to post-minimization structre (pre-equilibration)
common to the triplicate runs.

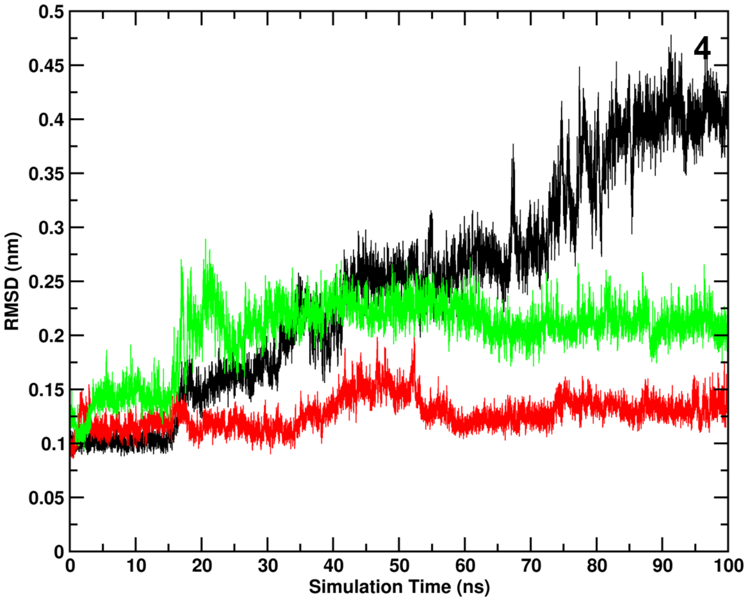

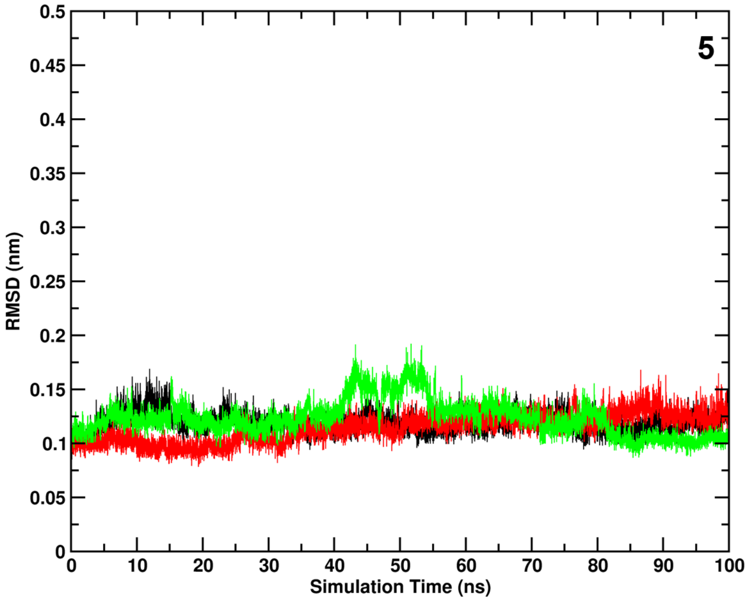

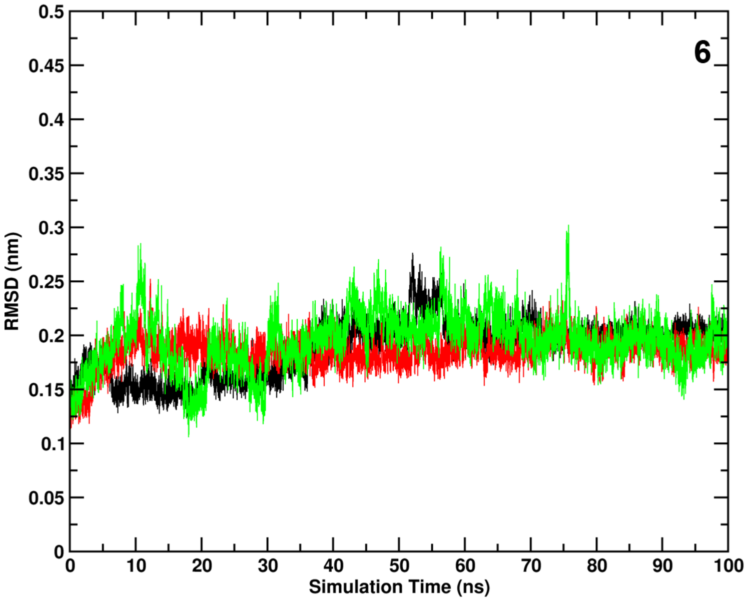

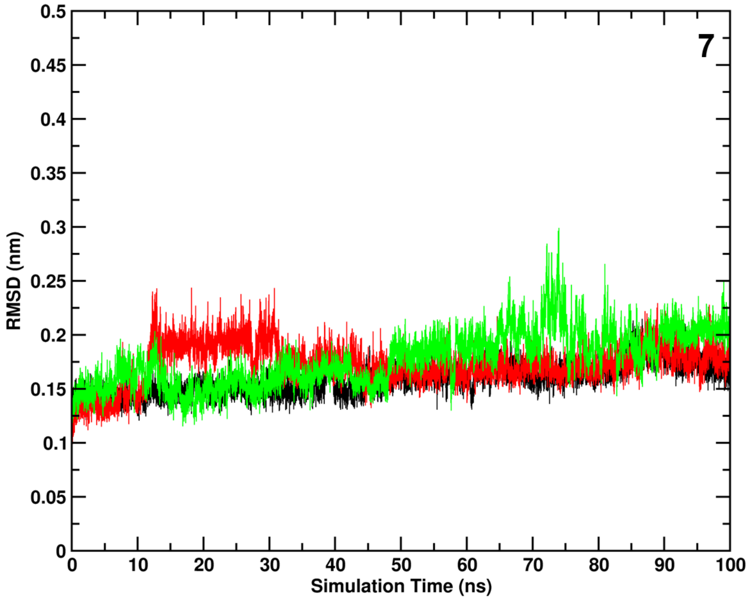

Supporting Information
Text S9

Root Mean Squared Deviation of Heavy Active Site Atoms for $N8_{closed}$ Systems

All values relative to post-minimization structre (pre-equilibration) common to the triplicate runs.

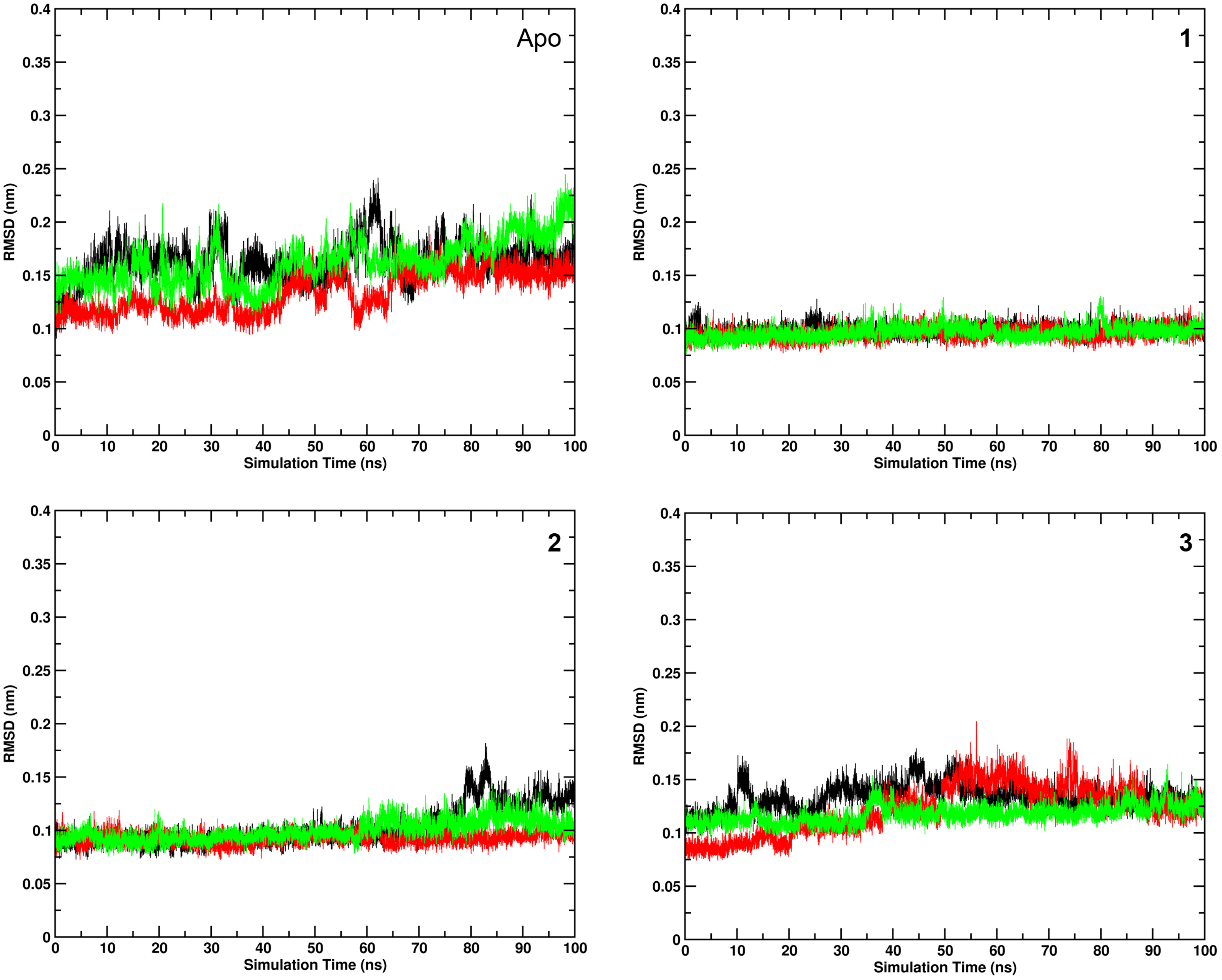

Supporting Information
Text S9

Root Mean Squared Deviation of Heavy Active Site Atoms for N8closed Systems

All values relative to post-minimization structre (pre-equilibration) common to the triplicate runs.

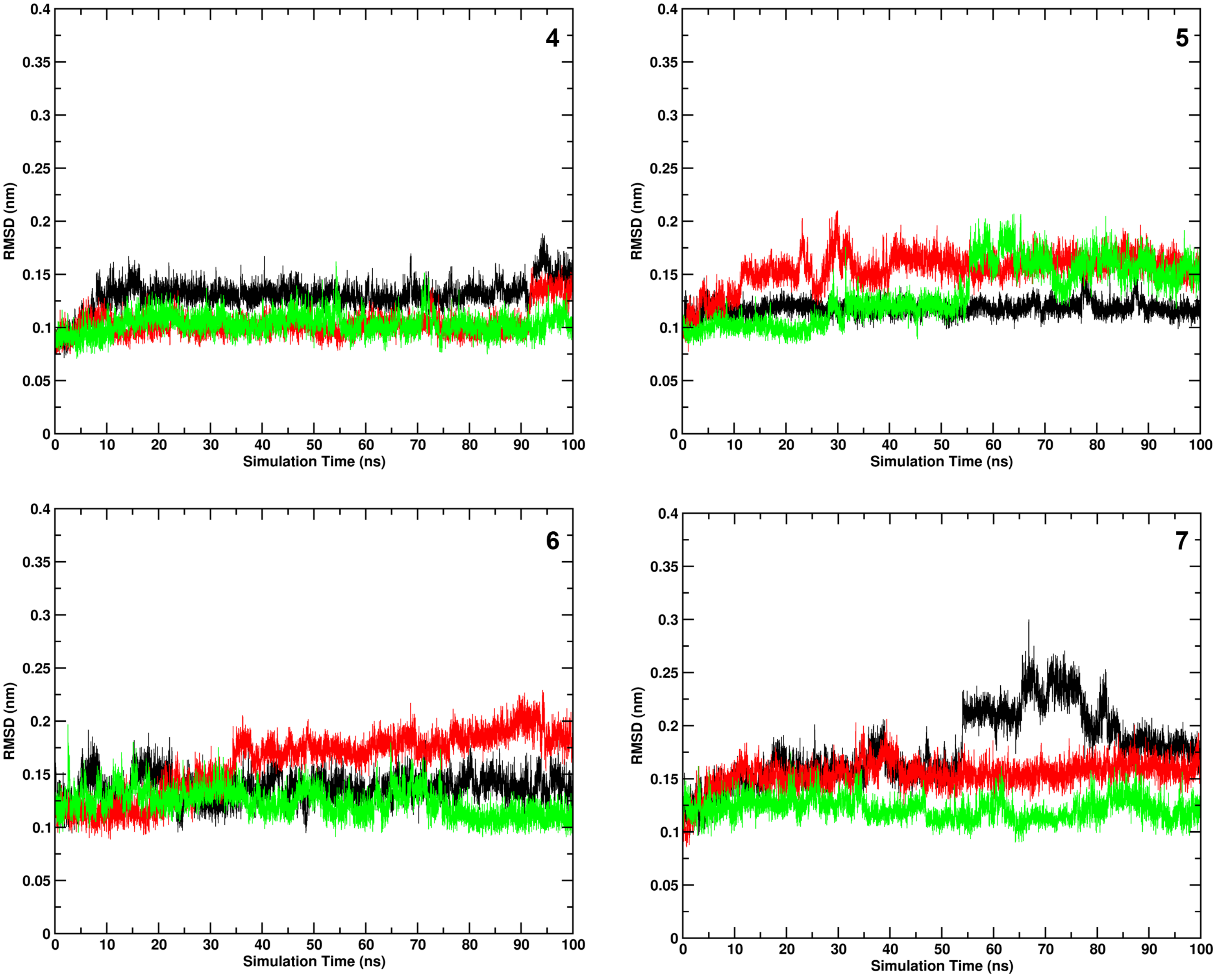

Supporting Information
Text S9

Root Mean Squared Deviation of Heavy Active Site Atoms for $N8_{open}$ Systems

All values relative to post-minimization structre (pre-equilibration)
common to the triplicate runs.

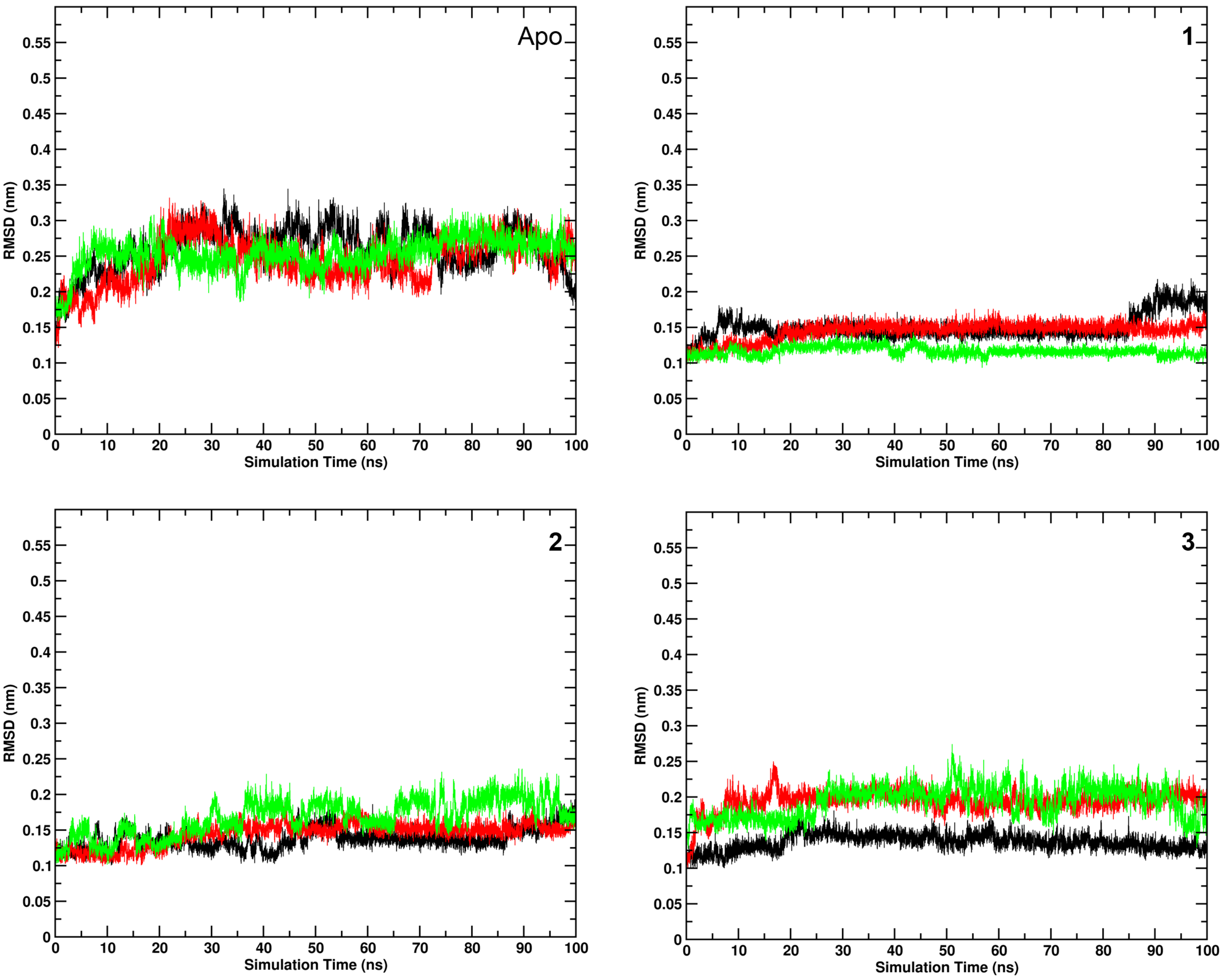

Supporting Information
Text S9

Root Mean Squared Deviation of Heavy Active Site Atoms for N8open Systems

All values relative to post-minimization structre (pre-equilibration) common to the triplicate runs.

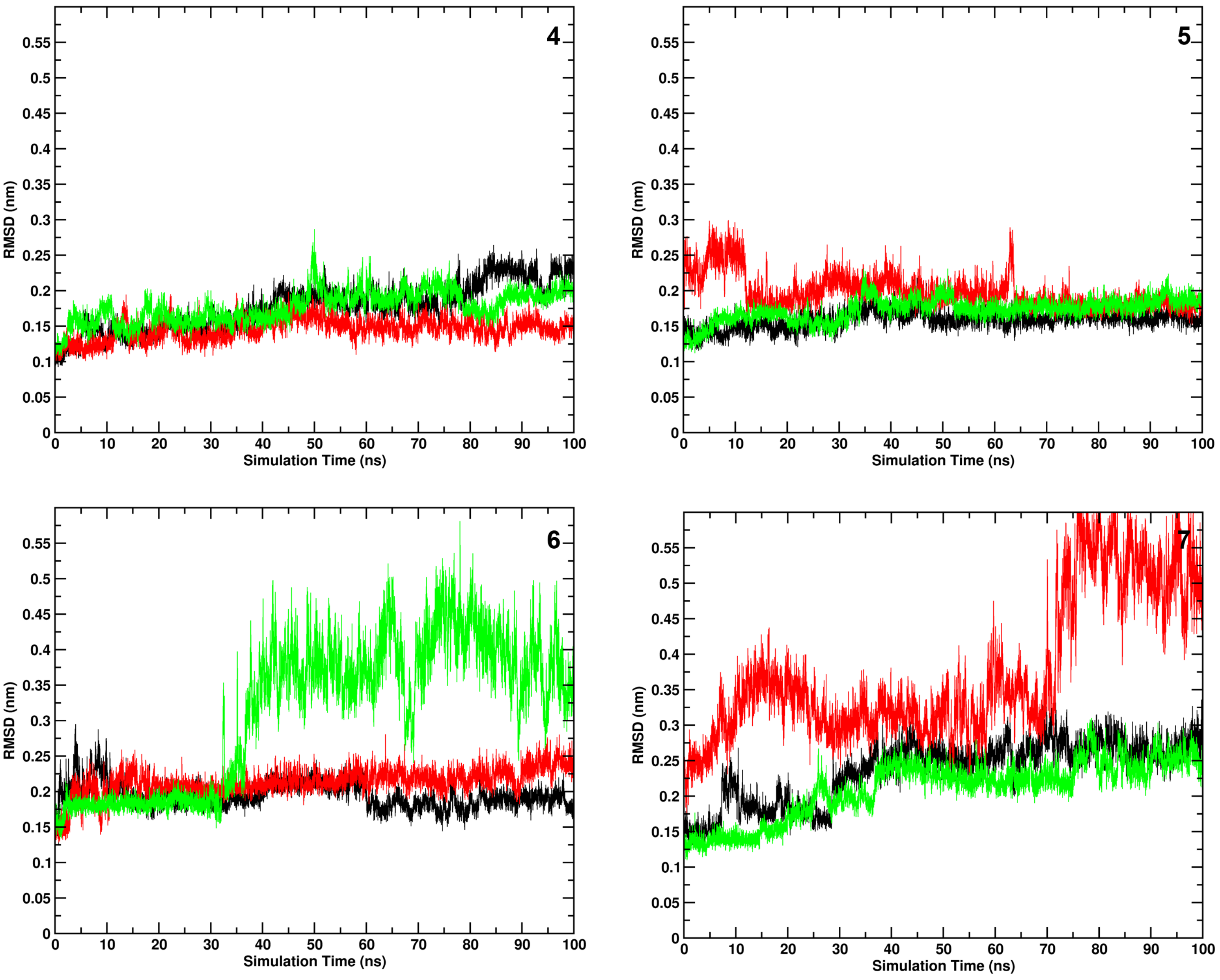

Supplement: Text S9 — Root mean squared deviation of heavy active site atoms for all simulations. (PDF) [file pone.0059873.s010.pdf]
